# Tubulin mRNA stability is sensitive to change in microtubule dynamics caused by multiple physiological and toxic cues

**DOI:** 10.1371/journal.pbio.3000225

**Published:** 2019-04-09

**Authors:** Ivana Gasic, Sarah A. Boswell, Timothy J. Mitchison

**Affiliations:** 1 Department of Systems Biology, Harvard Medical School, Boston, Massachusetts, United States of America; 2 Department of Systems Biology, Program in Therapeutic Sciences, Harvard Medical School, Boston, Massachusetts, United States of America; Utrecht University, NETHERLANDS

## Abstract

The localization, mass, and dynamics of microtubules are important in many processes. Cells may actively monitor the state of their microtubules and respond to perturbation, but how this occurs outside mitosis is poorly understood. We used gene-expression analysis in quiescent cells to analyze responses to subtle and strong perturbation of microtubules. Genes encoding α-, β, and γ-tubulins (TUBAs, TUBBs, and TUBGs), but not δ- or ε-tubulins (TUBDs or TUBEs), exhibited the strongest differential expression response to microtubule-stabilizing versus destabilizing drugs. Quantitative PCR of exon versus intron sequences confirmed that these changes were caused by regulation of tubulin mRNA stability and not transcription. Using tubulin mRNA stability as a signature to query the Gene Expression Omnibus (GEO) database, we find that tubulin genes respond to toxins known to damage microtubules. Importantly, we find many other experimental perturbations, including multiple signaling and metabolic inputs that trigger tubulin differential expression, suggesting their novel, to our knowledge, role in the regulation of the microtubule cytoskeleton. Mechanistic follow-up confirms that one important physiological signal, phosphatidylinositol-4,5-bisphosphate 3-kinase (PI3K) activity, indeed regulates tubulin mRNA stability via changes in microtubule dynamics. We propose that tubulin gene expression is regulated as part of many coordinated biological responses, with wide implications in physiology and toxicology. Furthermore, we present a new way to discover microtubule regulation using transcriptomics.

## Abstract 2

How do cells monitor the state of their microtubules? A genomic study reveals differential gene expression signatures characteristic to microtubule damage, and bioinformatic use of this signature identifies non-microtubule targeting conditions that influence the microtubule cytoskeleton.

## Introduction

α- and β-tubulin (TUBA and TUBB) proteins form obligate heterodimers that, through the nucleating activity of γ-tubulin (TUBG), polymerize into microtubules. Microtubules physically organize eukaryotic cells, serve as platforms for intracellular transport and signaling, and power cell division [1]. The localization, mass, and dynamics of microtubules must be precisely tuned for the microtubule cytoskeleton to execute its many functions. To ensure this, it seems likely that cells actively monitor the state of their microtubules and generate responses specific to perturbation. During mitosis, the Spindle Assembly Checkpoint (SAC) monitors the microtubules of the mitotic spindle and controls anaphase progression [2]. Efficacy of microtubule-targeting cancer chemotherapy has been associated with the activation of the SAC and mitotic arrest, which hinders proliferation and ultimately results in cell death [3]. However, many tumors have a low proliferation rate—known as “proliferation rate paradox” [4]—and it remains a puzzle how targeting mitosis only can eradicate such tumors. Evidence is growing that microtubule-damaging chemotherapy also triggers active signaling responses in nondividing cells, which may contribute to interphase cell death and tumor shrinkage [5]. Specifically, drug-induced microtubule damage in interphase has been associated with changes in cell-cycle progression [6–8], activation of mitogen-activated protein kinase (MAPK) signaling pathways [9–11], and activation of guanine nucleotide exchange factors for Rho-family GTPases [12]. These data show that interphase cells respond to microtubule damage through some signaling pathways, understanding of which will be critical for fundamental biology and pharmacology of microtubule-targeting therapy. However, a comprehensive view is missing, leaving a deliberate surveillance mechanism of the microtubule cytoskeleton in interphase cells speculative.

One route to discovering if, and how, interphase cells sense and respond to changes in the microtubule cytoskeleton is to measure differential gene expression (DGE) as a function of drug-induced microtubule damage. Naively, one might expect microtubule-stabilizing and destabilizing drugs to have opposite effects on the expression of genes involved in monitoring microtubule damage. Consistent with this hypothesis, microtubule destabilization suppresses, while stabilization activates, synthesis of TUBA and TUBB [13–15]. The differential biosynthesis of tubulins is a result of a post-transcriptional regulation of tubulin mRNA. This mechanism, known as tubulin autoregulation, is a negative feedback loop that involves indirect cotranslational regulation of the stability of mature spliced, but not unspliced, tubulin pre-mRNA by unpolymerized tubulin [13–15]. It remains unknown whether tubulin autoregulation is part of a larger DGE program. Several studies analyzed the effect of one or multiple microtubule-targeting drugs on gene expression using genome-wide methods [16–18]. One large-scale study found similar, rather than opposite, effects of microtubule-stabilizing versus destabilizing drugs [16]. A plausible explanation is that both stabilizers and destabilizers cause mitotic arrest, leading to common downstream effects on gene expression. The resulting changes in gene expression are thus likely to reflect cell-cycle regulation more than microtubule-specific signaling.

We sought to discover specific gene networks that respond differentially to microtubule-stabilizing versus destabilizing drugs. To avoid a mitotic arrest signature, we used quiescent cells. Our study revealed several DGE signatures that clearly exhibited opposite responses to microtubule destabilization versus stabilization. We found that TUBA, TUBB, and TUBG genes exhibit the strongest differential regulation. Using tubulin DGE as a query in bioinformatics analyses, we discovered multiple physiological inputs that changed tubulin gene expression, including the phosphatidylinositol-4,5-biphosphate (PI3K) signaling pathway. Follow-up mechanistic analysis confirmed that PI3K activity regulated tubulin gene expression via changes in microtubule stability, leading to autoregulation of tubulin mRNAs. Our study reveals a new role of tubulin gene-expression regulation as part of concerted responses of cells to multiple physiological or damaging inputs.

## Results

### Gene-expression profiles of microtubule perturbation in quiescent cells

**Fig 1:**
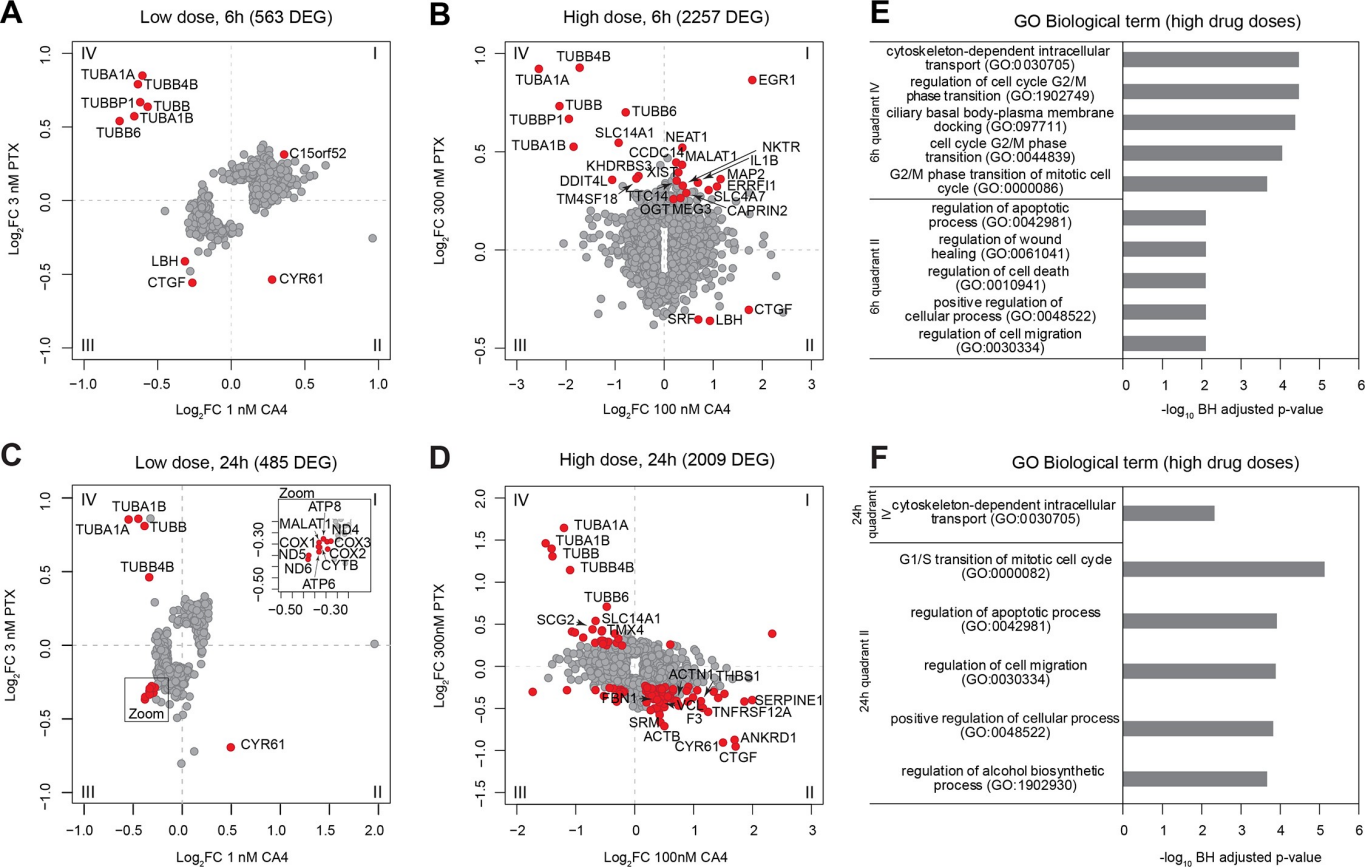
Microtubule damage triggers DGE. (A-D) DGE in RPE1 hTert cells treated with microtubule poisons for 6 (a and b) or 24 h (c and d). Data are represented as average Log_2_FCs from three independent biological replicates, relative to basal expression in DMSO-treated control cells. Plotted are expression profiles of genes that have *p*-value < 0.05 and >50 mRNA reads per million, in 1 nM CA4- and 3 nM PTX-treated cells (a and c) and 100 nM CA4- and 300 nM PTX-treated cells (b and d). In red are depicted genes with FDR < 0.02 for low drug doses and < 0.001 for high drug doses, of which names are printed for top 20 genes. Plot quadrants are labeled from I–IV. (E and F) GSEA based on expression profiles of genes from quadrants II and IV, from cells treated with high drug doses for 6 (b) or 24 h (d). Plotted are −Log_10_ BH-adjusted *p*-values of the top five enriched GO terms in the category Biological Process, with BH-adjusted *p*-value < 0.01. ACTB, β-actin; ACTN, actinin; ANKRD, Ankyrin Repeat Domain; BH, Benjamini–Hochberg; CA4, combretastatin A-4; CCDC, Coiled-coil domain containing; COX, Cytochrome C Oxidase; CTGF, Connective Tissue Growth Factor; CYR, Cytochrome Reductase; CYTB, Cytochrome B; DEG, differentially expressed gene; DGE, differential gene expression; EGR, Early Growth Response; ERRF, Endoplasmatic Reticulum-Related Factor; FDR, false discovery rate; GO, gene ontology; GSEA, gene set enrichment analysis; hTert, human telomerase reverse transcriptase; KHDRBS, KH RNA Binding Domain Containing, Signal Transduction Associated; LBH, Limb Bud And Heart Development; Log_2_FC, Log_2_ Fold Change; MALAT, Metastasis-Associated Lung Adenocarcinoma Transcript; MAP, microtubule-associated protein; MEG, Maternally Expressed Gene; ND, NADH Dehydrogenase; NEAT, Nuclear Paraspeckle Assembly Transcript; NKTR, Natural Killer Cell Triggering Receptor; OGT, O-Linked N-Acetylglucosamine Transferase; PTX, paclitaxel; RPE1, retinal pigment epithelial 1; SCG, Secretogranin; SLC, Solute Carrier Family; SRF, Serum Response Factor; SRM, Spermidine Synthase; THBS, Thrombospondin; TMX, Thioredoxin Related Transmembrane Protein; TM4SF, Transmembrane 4 L Six Family Member; TNFRSF, Tumor Necrosis Factor Receptor Superfamily Member; TTC, Tetratricopeptide Repeat Domain; TUBA, α-tubulin; TUBB, β-tubulin; XIST, X Inactive Specific Transcript.

To identify interphase-specific responses to microtubule damage, we first established conditions of healthy quiescence in a reference cell type for cell biology studies. Retinal pigment epithelial 1 (RPE1) cells, immortalized by the expression of human telomerase reverse transcriptase (hTert), were grown to and maintained as confluent cultures for 5 days prior to treatment with microtubule-destabilizing and stabilizing drugs. Cell-cycle profiling showed strong G1 arrest and no difference in cell-cycle state between control and drug treated cells after 24 h (S1A Fig). Combretastatin A-4 (CA4), which binds tubulin at the colchicine site, was chosen as a reference destabilizer, and paclitaxel (PTX) as a reference stabilizer [19] (S1B–S1E Fig). We chose drug concentrations based on tubulin partitioning between polymer and dimer and counting growing microtubule plus-tips labeled with end-binding protein 1 (EB1) [20]. Low doses (1 nM CA4, 3 nM PTX) were chosen to cause barely detectable effects, and high doses (100 nM CA4, 300 nM PTX) to cause complete loss of polymer (destabilizer) or complete loss of soluble dimer (stabilizer) (S1B–S1E Fig). We then compared gene-expression responses by RNA sequencing (RNA-seq) of polyadenylated (polyA+) mRNAs at 6 and 24 h post-drug treatment. These data revealed differential expression of multiple genes under all conditions relative to basal expression (Benjamini–Hochberg corrected *p*-value < 0.05, and >50 mRNA counts per million reads, Fig 1A–1D).

DGE relative to vehicle treated cells (Fig 1A–1D) is presented to reveal genes that respond similarly to both drugs (quadrants I and III) versus differentially (quadrants II and IV, Fig 1A–1D). As expected, high drug doses caused extensive gene-expression changes. Surprisingly, subthreshold doses also caused many significant changes, showing that quiescent cells can detect even mild perturbation of microtubule dynamics. Based on high-drug-dose–expression profiles, we performed gene set enrichment analysis (GSEA), finding significantly enriched gene ontology (GO) terms (Fig 1E and 1F). The most prominently enriched are genes involved in G1/S transition of the mitotic cell cycle, which were up-regulated in CA4-treated cells and down-regulated in PTX-treated cells (Fig 1F). These data are consistent with previous reports that microtubule destabilization promotes DNA replication and cell-cycle re-entry, while stabilization inhibits it [21–23]. We further found highly enriched genes involved in apoptosis, wound healing, and cell migration, which were also up-regulated upon microtubule destabilization and down-regulated upon stabilization (Fig 1E and 1F). Surprisingly, we found only one cluster of microtubule-related genes involved in cytoskeleton-based transport (6 h and 24 h post-treatment) that were differentially regulated by microtubule stabilization versus destabilization (Fig 1E and 1F). We conclude that cells mount both differential and common responses to microtubule stabilization versus destabilization. Furthermore, they clearly detect damage that is subthreshold by biochemical tubulin-partitioning criteria. Notably, we did not observe changes in mitosis-related genes in quadrants I and III, consistent with the lack of mitotic arrest in our quiescent cultures. Some common DGE signatures were suggestive of general stress responses, such as apoptosis, and these deserve further analysis.

Tubulin genes stood out as the most differentially regulated genes in quadrant IV in all four plots, particularly in the low-dose perturbations (Fig 1A–1D). This observation was statistically confirmed by GSEA analysis, in which tubulin differential expression drove enrichment of GO terms such as “cytoskeleton-dependent intracellular transport” (Fig 1E and 1F). We conclude that coordinated change in multiple tubulin mRNAs was the strongest response to drug-induced microtubule damage, with destabilizing drugs decreasing tubulin mRNA concentrations and stabilizing drugs increasing them.

### Differential regulation of most tubulin isoforms upon microtubule damage

**Fig 2:**
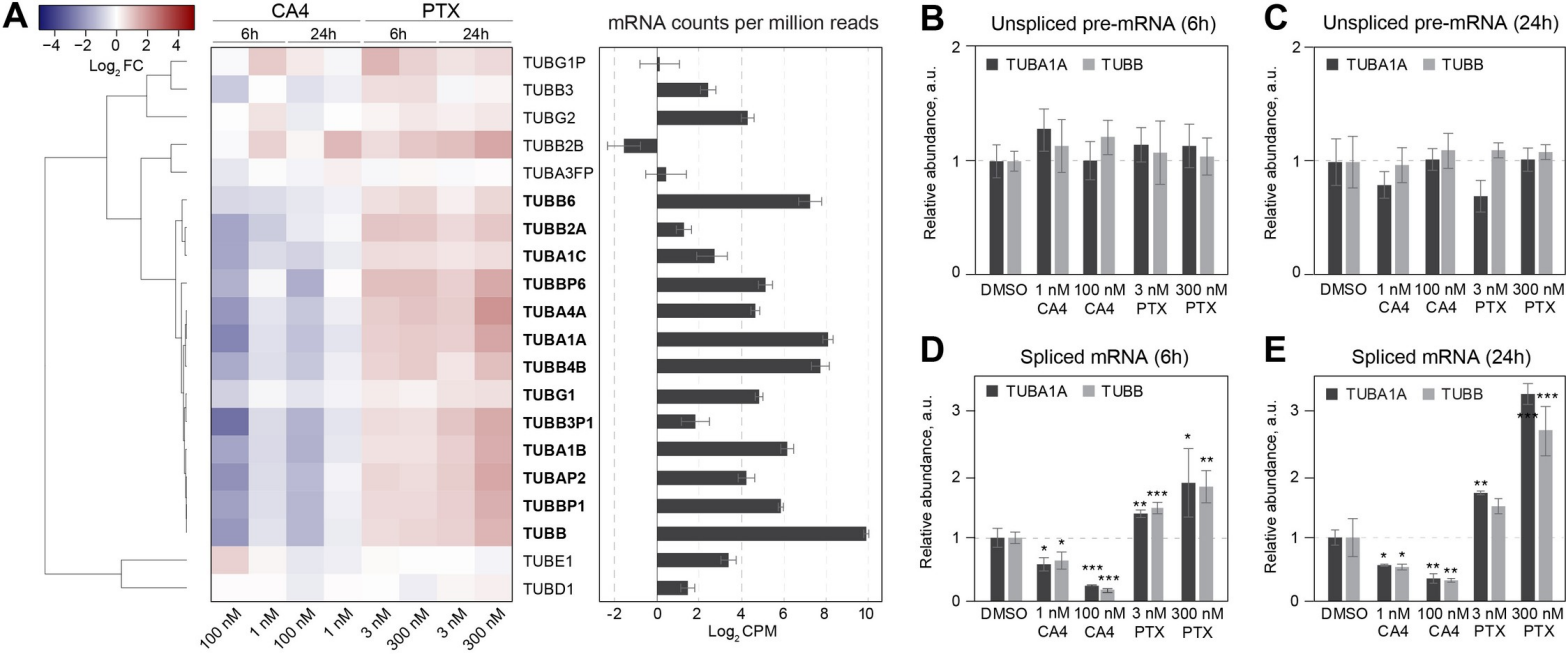
Microtubule damage triggers differential expression of all TUBA and TUBB isoforms. (A, left panel) Expression profiles of all detected TUBA and TUBB isoforms in our DGE data set. Dendrogram on the left represents Pearson distance between expression profiles. Each column of the heatmap represents DGE in one treatment, labeled on the *x*-axis above and below the heatmap, relative to DMSO control. Each row represents a gene, labeled on the *y*-axis. In bold are differentially expressed genes that have *p*-value < 0.05. Color key is depicted in upper left corner. Data are represented as Log_2_FC relative to DMSO control. (A, right panel) Average Log_2_ mRNA CPMs in DMSO-treated control samples are presented as a bar chart. (B and C) Relative abundance of unspliced TUBA1A and TUBB pre-mRNA in control (DMSO) and cells treated with microtubule poisons (*x*-axis) for 6 (B) or 24 h (C). (D and E) Relative abundance of spliced TUBA1A and TUBB mRNA in control (DMSO) and cells treated with microtubule poisons (*x*-axis) for 6 (D) or 24 h (E). All the expression profiles are normalized to a reference gene (GAPDH or RPL19) and to DMSO control. Error bars in all panels represent standard deviation from three independent biological replicates. *p-value < 0.05, **p-value < 0.01, ***p-value < 0.001 in paired Student *t* test compared to DMSO control. CA4, combretastatin A-4; CPM, count for each gene per million detected reads; DGE, differential gene expression; GAPDH, Glyceraldehyde 3-phosphate dehydrogenase; Log_2_FC, Log_2_ Fold Change; PTX, paclitaxel; RPL19, ribosomal protein L19; TUBA, α-tubulin; TUBB, β-tubulin; TUBD, δ-tubulin; TUBE, ε-tubulin; TUBG, γ-tubulin.

To test if all tubulin mRNAs undergo similar regulation, we extracted the expression profiles of all detected tubulin genes in our data set, and their total mRNA counts (Fig 2A). We observed differential regulation of all highly expressed TUBA and TUBB mRNAs, but not centrosomal δ- (TUBD1) and ε-tubulins (TUBE1) (Fig 2A). Moreover, we found clear differential expression of centrosomal TUBG isoform 1 (TUBG1) and borderline regulation of isoform 2 (TUBG2) (Fig 2A).

To generalize this finding, we reanalyzed two large, high-quality data sets deposited in the Gene Expression Omnibus (GEO) database that profiled DGE response to microtubule damage. In an extensive study that compared PTX with eribulin (ERB, a microtubule destabilizer) treatment of many breast, ovarian, and endometrial cancer cell lines [16], we confirmed differential regulation of all expressed TUBAs and TUBBs and TUBG1 (S2A Fig). Importantly, reanalyzing a study that compared the effect of microtubule destabilizers colchicine, vinblastine, and vincristine on rat heart endothelial cells [24], we show for the first time differential regulation of tubulin genes in vivo (GEO GSE19290, S2B Fig). We conclude that cells differentially regulate all the expressed TUBA and TUBB isoforms and TUBG1 upon microtubule damage, both ex vivo and in vivo.

The microtubule-damage–induced changes in tubulin mRNA concentrations that we observed were strongly suggestive of tubulin autoregulation, a post-translational gene-expression regulation mechanism [25]. RNA-seq of polyA+ mRNA does not distinguish between transcriptional and post-transcriptional regulatory mechanisms because the sample is enriched for spliced mRNA. Similarly, most microarray assays target exclusively the exonic sequences of mRNAs, making it impossible to distinguish the regulation of unspliced and spliced mRNA and draw conclusions about transcriptional versus post-transcriptional gene-expression regulation. To make this determination, we established a reverse-transcription quantitative PCR-based assay (RT-qPCR) to specifically measure transcriptional regulation through the expression levels of unspliced pre-mRNA and post-transcriptional regulation through the expression levels of spliced mRNA (S2C Fig). Using this approach, we measured two highly expressed tubulin genes, TUBA1A and TUBB, and two control housekeeping genes, Glyceraldehyde 3-phosphate dehydrogenase (GAPDH) and ribosomal protein L19 (RPL19). We found no significant change in unspliced TUBA1A and TUBB pre-mRNA concentration in cells treated with CA4 or PTX (Fig 2B and 2C), showing that microtubule damage did not change the rate of tubulin gene transcription. However, levels of mature, spliced TUBA1A and TUBB mRNAs significantly diminished in CA4-treated cells and increased in PTX-treated cells (Fig 2D and 2E), consistent with our RNA-seq data. We conclude that post-transcriptional regulation of tubulin mRNA stability is the most prominent gene-expression response to microtubule damage. Importantly, we did not observe coregulation of any microtubule-interacting proteins, such as microtubule-associated, motor, or plus-tip–binding proteins. Thus, altered stability of microtubules only regulates the expression of tubulins, but not the other components of functional microtubules.

### Bioinformatic analysis of the autoregulation signature reveals new microtubule biology

**Fig 3:**
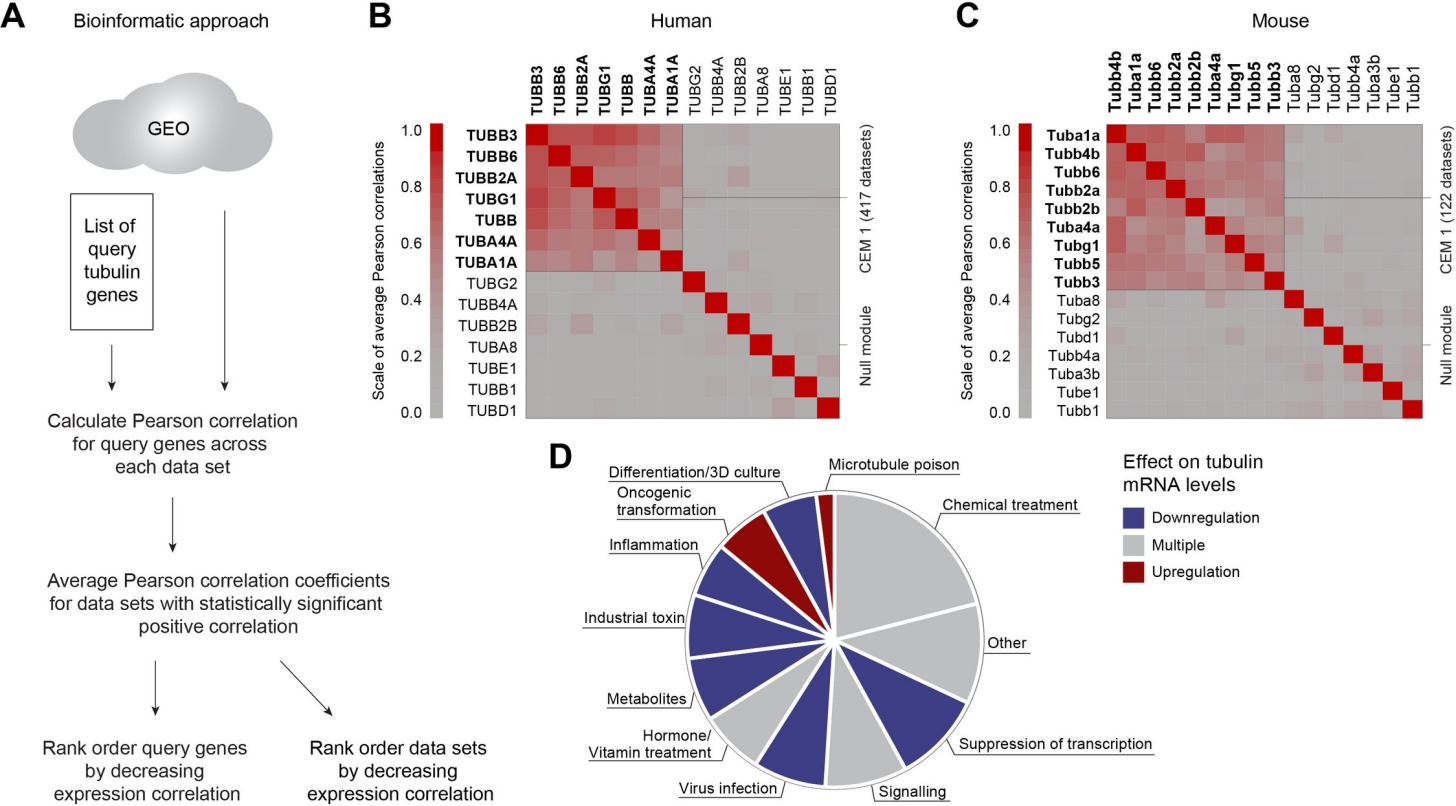
Cells coordinate expression of TUBA and TUBB isoforms. (A) Scheme of the bioinformatic approach. (B and C) Pearson expression correlation coefficients for a subset of abundantly expressed TUBA and TUBB isoforms across 417 human (B) and 122 mouse (C) publicly available Affymetrix chip data sets. In dark red are genes that show strong expression correlation (Pearson correlation coefficient = 1), and in gray are genes that show no expression correlation (Pearson correlation coefficient = 0). Color keys are represented to the left of each heatmap. High expression correlation for a subset of tubulin genes is marked as cluster CEM1. Tubulin genes that do not correlate in expression are marked as Null module. (D) Frequency diagram of groups of perturbations among the top 100 data sets with the highest tubulin gene-expression correlation from the human platform. CEM, Co-Expressed Modules; GEO, Gene Expression Omnibus; TUBA, α-tubulin; TUBB, β-tubulin; TUBD, δ-tubulin; TUBE, ε-tubulin; TUBG, γ-tubulin.

We next sought to investigate whether tubulin DGE is a general response to altered microtubule dynamics in conditions other than microtubule-targeted poisoning. The differential tubulin gene expression triggered by microtubule damage comprises a strong and specific signature that can be used to query publicly available DGE datasets in an unbiased manner and with the expectation of finding novel conditions that regulate microtubules. To test this approach, we used CLustering by Inferred Co-expression [26] (CLIC, https://gene-clic.org, Fig 3A)—a bioinformatic tool that mines approximately 3,500 publicly available human and mouse microarray studies deposited in the GEO database. Importantly, most of these studies are not designed to research cellular response to microtubule damage, providing an unbiased approach that can potentially reveal new microtubule biology.

We first investigated whether tubulin genes were coordinately expressed across many sample and perturbation types. We found high Pearson expression correlation coefficients for the most broadly expressed tubulin genes in 417 human (Fig 3B) and 122 mouse studies (Fig 3C). More specialized and low-abundance tubulin isoforms displayed lower expression correlation coefficients. The obtained expression correlations confirm that cells coregulate the mRNAs for multiple TUBA and TUBB isoforms and TUBG1 in many cell and tissue types across a total of 539 different perturbations. These perturbations are candidates for novel conditions that regulate the microtubule cytoskeleton.

To understand what kinds of experimental perturbations induce coregulation of tubulin genes and potentially regulate the microtubule cytoskeleton, we rank-ordered the human data sets by descending Pearson correlation coefficient (S2 Table) and manually annotated the top 100 (Fig 3D and Table 1). Among high-ranked studies were multiple investigations of established microtubule drugs, such as PTX, or toxins known to perturb microtubules, such as bisphenol-A (Table 1). These served as positive controls that our bioinformatic analysis returned studies in which microtubules were perturbed. We also discovered many novel, to our knowledge, conditions, including virus infection, metabolite deprivation, exposure to industrial toxins, inflammation, and cell differentiation, which all tended to cause coordinated down-regulation of tubulin mRNAs (Fig 3D and Table 1). Surprisingly, only one process, oncogenic transformation, consistently up-regulated tubulin mRNAs (Fig 3D and Table 1). Hormone, vitamin, and chemical treatment, as well as perturbations of signaling, had molecule-specific effects on tubulin gene expression (Fig 3D, Table 1). In some studies, inspection of the CLIC report showed that coregulation of tubulin was not induced by the experimental perturbation in the title of the study, but rather by some other controlled and annotated experimental variable. For example, Steroid Receptor Coactivator-1 (SRC1) RNAi in A549 lung cancer cells did not change tubulin gene expression compared to control RNAi, but glucose withdrawal, which was included as a variable in the same study, suppressed it [27] (S3A Fig).

**Table 1:** The top 100 data sets with highest tubulin-gene–expression Pearson correlation from the human platform. Annotated are studies rank-ordered by decreasing tubulin-gene–expression Pearson correlation, with their associated GEO identifiers. Extracted from the CLIC report is differential tubulin gene expression. Unless NA, down-regulated (down) or up-regulated (up) tubulin mRNA levels are annotated. Based on published literature, perturbations in the listed studies are annotated into 11 groups, and their effect on microtubule cytoskeleton is marked as known (yes) or unknown (no).

GSE	Title of the Study	Average Pearson Correlation Score	DGE of Tubulin, Up/Down/NA	Annotation	Known Microtubule Poison, Yes/No
GSE59931	Glutamine deprivation in U2OS cells	0.98	down	Metabolites	yes
GSE36529	Expression data from CtBP knockdown MCF-7 cells	0.98	up	Gene expression	no
GSE32158	Bisphenol A Regulates the Expression of DNA Repair Genes in Human Breast Epithelial Cells (expression data)	0.97	up	Industrial toxin	yes
GSE23952	Expression data from TGF-beta treated Panc-1 pancreatic adenocarcinoma cell line	0.97	up	Inflammation	no
GSE22522	Comparison of the transcriptome of K-LEC spheroids to control LEC spheroids	0.96	up	Chemical treatment	no
GSE56843	Steroid Receptor Coactivator 1 is an Integrator of Glucose and NAD(+)/NADH Homeostasis	0.96	down	Metabolites	no
GSE20719	Gene expression changes upon treatment of T47D breast cancer cells with the Pan-PI3 kinase inhibitor GDC-0941	0.96	down	Signaling	yes
GSE4217	Spheroid Formation and Recovery of Human Foreskin Fibroblasts at Ambient Temperature	0.95	down	Culture condition	no
GSE46708	CD24 targets	0.94	down	Inflammation	no
GSE35428	Transcriptional profiling of clinically relevant SERMs and SERM/estradiol complexes in a cellular model of breast cancer	0.94	NA	Hormone/vitamin treatment	yes
GSE7745	Mapping of HNF4 binding sites, acetylation of histone H3 and expression in Caco2 cells	0.92	down	Gene expression	no
GSE46924	27-Hydroxycholesterol links cholesterol and breast cancer pathophysiology.	0.91	up	Hormone/vitamin treatment	no
GSE58605	Expression data from A549 cells infected by adenovirus not carrying virus associated sequences in the genome	0.90	down	Virus infection	yes
GSE4218	Spheroid Formation and Recovery of Human T98G Glioma Cells at Ambient Temperature	0.89	down	Culture condition	no
GSE15499	HDAC5 is a repressor of angiogenesis and determines the angiogenic gene expression pattern of endothelial cells	0.89	NA	Signaling	no
GSE52659	Expression data from WEEV infected BE(2)-C/m cells	0.88	down	Virus infection	yes
GSE10444	gene expression levels in long-term cultures of human dental pulp stem cells	0.88	down	Differentiation/3D culture	no
GSE43700	Microarray analysis of IL-10 stimulated adherent peripheral blood mononuclear cells	0.88	up	Inflammation	no
GSE29625	Human embryonic stem cells derived from embryos at different stages of development share similar transcription profiles	0.87	NA	Differentiation/3D culture	yes
GSE12098	Comparison of the migration profile of MSCs	0.86	NA	Chemical treatment	no
GSE36085	Regulation of Autophagy by VEGF-C axis in cancer	0.86	NA	Signaling	no
GSE32161	Microarray analysis of genes associated with cell surface NIS protein levels in breast cancer	0.85	down	Metabolites	no
GSE42733	Gene expression profile of Nurse-Like Cells (NLC) derived from chronic lymphocytic leukemia	0.85	up	Transformation	no
GSE36176	Gene expression arrays on lung cancer cells exposed to Notch inhibitor	0.85	NA	Chemical treatment	no
GSE16070	Networking of differentially expressed genes in human MCF7 breast cancer cells resistant to methotrexate	0.84	up	Chemical treatment	no
GSE23399	Gene expression profling of human breast carcinoma-associated fibroblasts treated with paclitaxol or doxorubicin at therapeutically relevant doses	0.84	up	Microtubule poison	yes
GSE17368	Epiphyseal cartilage	0.84	NA	Other	no
GSE14001	PAX2: A Potential Biomarker for Low Malignant Potential Ovarian Tumors and Low-Grade Serous Ovarian Carcinomas	0.83	down	Gene expression	no
GSE16659	Expression data of HGF/cMET pathway in prostate cancer DU145 cell line	0.83	NA	Signaling	no
GSE19136	Gene expression response to implanted drug (paclitaxel)-eluting or bare metal stents in denuded human LIMA arteries	0.83	up	Microtubule poison	yes
GSE14773	Roles of EMT regulator in colon cancer	0.83	up	Transformation	no
GSE31641	Expression data from treatment of human melanocytes with phenolic compounds	0.82	down	Industrial toxin	no
GSE13142	HepG2/C3A cells cultured for 42 h in complete or leucine-devoid medium	0.81	down	Metabolites	no
GSE19495	Global Gene Expression of Human Hepatoma Cells After Amino Acid Limitation	0.81	down	Metabolites	no
GSE9649	Expression studies of HMEC exposed to lactic acidosis and hypoxia	0.81	multiple	Chemical treatment	no
GSE7345	Germline NRAS mutation causes a novel human autoimmune lymphoproliferative syndrome	0.81	NA	Signaling	no
GSE5838	Expression data from transplanted intestine bifor signs of rejection, and when their was signs of rejection	0.81	NA	Other	no
GSE42853	Distinct gene expression profiles associated with the susceptibility of pathogen-specific CD4 T cells to HIV-1 infection	0.80	NA	Virus infection	no
GSE9835	Gene Expression Changes in Response to Baculoviral Vector Transduction of Neuronal Cells In Vitro	0.80	down	Virus infection	yes
GSE34635	Defining a No Observable Transcriptional Effect Level (NOTEL) for low dose N-OH-PhIP exposures in human BEAS-2B bronchioepithelial cells	0.80	up	Industrial toxin	no
GSE12875	Impaired T-cell function in patients with novel ICOS	0.79	up	Inflammation	no
GSE8588	OH-PBDE-induced gene expression profiling in H295R adrenocortical carcinoma cells	0.79	down	Industrial toxin	no
GSE17044	Expression data from androgen treated LNCaP cells	0.79	NA	Hormone/vitamin treatment	no
GSE33143	Targeted disruption of the BCL9/beta-catenin complex in cancer	0.79	up	Signaling	no
GSE16089	Networking of differentially expressed genes in human Saos-2 osteosarcoma cells resistant to methotrexate	0.79	up	Chemical treatment	no
GSE51130	Using a rhabdomyosarcoma patient-derived xenograft to examine precision medicine approaches and model acquired resistance	0.79	up	Chemical treatment	no
GSE49085	Identification of bone morphogenetic protein (BMP)-7 as a key instructive factor for human epidermal Langerhans cell differentiation and proliferation	0.78	NA	Differentiation/3D culture	no
GSE53731	Expression data from hepatitis E virus inoculated PLC/PRF/5 cells	0.78	NA	Virus infection	yes
GSE44540	Gene expression in hTERT-RPE1 cells with overexpression of MFRP	0.78	up	Other	no
GSE15065	C/EBPbeta-2 regulation of gene expression in MCF10A cells	0.78	NA	Chemical treatment	no
GSE19510	Transcriptional response of normal human lung WI-38 fibroblasts to benzo[a]pyrene diol epoxide: a dose-response study	0.78	down	Industrial toxin	no
GSE16356	Lymphatic endothelial cells (LEC) treated with a MAF-targeted siRNA	0.78	down	Gene expression	no
GSE26884	Bisphenol A Induced the Expression of DNA Repair Genes in Human Breast Epithelial Cells	0.77	down	Industrial toxin	yes
GSE45636	eIF3a in Urinary Bladder Cancer, in vivo and in vitro insights	0.77	down	Gene expression	no
GSE9677	Gene expression profile in HUVECs before and after Angiopoietin stimulation	0.77	NA	Chemical treatment	no
GSE5110	48h Immobilization in human	0.77	down	Other	no
GSE23764	Expression data from actomyosin contractility regulated genes	0.77	up	Chemical treatment	no
GSE17785	Endogenous expression of an oncogenic PI3K mutation leads to activated PI3K pathway signaling and an invasive phenotype	0.77	up	Signaling	no
GSE34512	PBEF Knockdown in HMVEC-LBI	0.76	up	Metabolites	no
GSE40517	Selective Requirement for Mediator MED23 in Ras-active Lung Cancer	0.75	up	Signaling	no
GSE33606	Gene expression changes in human hepatocytes exposed to VX (O-ethyl S-[2-(diisopropylamino)ethyl] methylphosphonothiolate)	0.75	NA	Chemical treatment	no
GSE2328	Application of genome-wide expression analysis to human health & disease	0.75	NA	Transformation	no
GSE29384	Tetracycline-Inducible Cyr61 effect on LN229 glioma cells	0.75	NA	Chemical treatment	no
GSE45417	Expression data from knockdown of ZXDC1/2 in PMA-treated U937	0.75	down	Gene expression	no
GSE37648	Gene signatures of normal hTERT immortalized ovarian epithelium and fallopian tube epithelium (paired cultures from 2 donor patients)	0.74	NA	Differentiation/3D culture	no
GSE6400	Cultured A549 lung cancer cells treated with actinomycin D and sapphyrin PCI-2050	0.74	up	Chemical treatment	no
GSE13378	Exposure of squamous esophageal cell line HET-1A to deoxycholic acid (DCA)	0.74	down	Chemical treatment	no
GSE37474	Dexamethasone induced gene expression changes in the human trabecular meshwork	0.74	NA	Chemical treatment	no
GSE28339	Gene expression data following Cyclin T2 and Cyclin T1 depletion by shRNA in HeLa cells	0.73	NA	Signaling	no
GSE20125	Transcriptome analysis of human Wharton's jelly stem cells: meta-analysis	0.73	multiple	Other	no
GSE22533	Breast cancer cells resistant to hormone deprivation maintain an estrogen receptor alpha-dependent, E2F-directed transcriptional program	0.73	down	Hormone/vitamin treatment	no
GSE38517	Expression data from fibroblasts derived from human normal oral mucosa, oral dysplasia and oral squamous cell carcinoma	0.72	up	Transformation	no
GSE47874	The Heritage (HEalth, RIsk factors, exercise Training And GEnetics) family study	0.72	multiple	Other	no
GSE39999	Filarial nematode AsnRS interacts with interleukin 8 receptors in iDCs but causes different gene expression patterns compared to iDCs stimulated by interleukin 8	0.72	up	Inflammation	no
GSE26599	Gene expression profile in response to doxorubicin-rapamycin combined treatment of HER-2 overexpressing human mammary epithelial cell lines	0.72	multiple	Chemical treatment	no
GSE20037	cdr2 siRNA knockdown during passage through mitosis: HeLa cells, Rat1 wild type and c-myc null cells	0.72	up	Inflammation	no
GSE33243	Human acute myelogenous leukemia-initiating cells treated with fenretinide	0.71	down	Chemical treatment	no
GSE12274	Mesenchymal Stromal Cells of Different Donor Age	0.71	multiple	Other	no
GSE4824	Analysis of lung cancer cell lines	0.71	multiple	Transformation	no
GSE13054	Genes upregulated by HLX	0.71	NA	Gene expression	no
GSE16524	Expression data from skin fibroblasts derived from Setleis Syndrome patients and normal controls	0.71	NA	Gene expression	no
GSE6494	Expression data from human liver cell line induced by PCB153	0.70	multiple	Chemical treatment	no
GSE32892	A genome-wide and dose-dependent inhibition map of androgen receptor binding by small molecules reveals its regulatory program upon antagonism	0.69	down	Hormone/vitamin treatment	no
GSE4289	Host transcriptome changes associated with episome loss and selection of keratinocytes containing integrated HPV16	0.69	down	Virus infection	no
GSE24224	Analysis of genome-wide methylation and gene expression induced by decitabine treatment in HL60 leukemia cell line	0.69	up	Chemical treatment	no
GSE17090	Expression data from human adipose stem cells expanded in allogeneic human serum and fetal bovine serum	0.68	down	Metabolites	no
GSE40220	Intestinal filter for use in oesophageal cancer research	0.68	NA	Other	no
GSE15372	Expression data from A2780 (cisplatin-sensitive) and Round5 A2780 (cisplatin-resistant) cell lines	0.68	up	Chemical treatment	no
GSE16538	Genome-wide gene expression profile analysis in pulmonary sarcoidosis	0.67	down	Other	no
GSE11208	Chronic nicotine exposure (kuo-affy-human-232930)	0.67	NA	Chemical treatment	no
GSE14986	Antiestrogen-resistant subclones of MCF-7 human breast cancer cells are derived from a common clonal drug-resistant progenitor	0.67	multiple	Hormone/vitamin treatment	no
GSE11352	Timecourse of estradiol (10nM) exposure in MCF7 breast cancer cells	0.67	up	Hormone/vitamin treatment	no
GSE16054	Transient expression of misfolded surfactant protein C	0.67	down	Other	no
GSE35170	Expression data from U87-2M1 glioma cells transduced with baculoviral control decoy vector or baculoviral miR-10b decoy vector	0.66	down	Virus infection	no
GSE31472	Host cell gene expression in Influenza A/duck/Malaysia/F118/08/2004 (H5N2) infected A549 cells at 2, 4, 6, 8, and 10 hours post infection	0.66	down	Virus infection	yes
GSE17624	Expression data from human Ishikawa cells treated with Bisphenol A	0.65	down	Industrial toxin	yes
GSE20540	Gene expression profiles of myeloma cells interacting with bone marrow stromal cells in vitro	0.61	up	Other	no
GSE18182	Expression profile of lung adenocarcinoma, A549 cells following targeted depletion of non-metastatic 2 (NME2/NM23 H2)	0.59	down	Gene expression	no
GSE30494	Microarray analysis to identify downstream genes after treatment with siKDM3A	0.58	NA	Gene expression	no
GSE33146	Expression data from DKAT breast cancer cell line pre- and post-EMT	0.57	down	Transformation	no

**Abbreviations**: CLIC, CLustering by Inferred Co-expression; DGE, differential gene expression; GEO, Gene Expression Omnibus; GSE, gene set enrichment; NA, not applicable.

To investigate whether our bioinformatic analysis returned new microtubule biology, we selected several perturbations, starting with nutrient deprivation, for validation by RT-qPCR and performed subsequent biochemical and microscopy-based inspection of the microtubule cytoskeleton. We confirmed that glucose and glutamine deprivation lower tubulin mRNA levels in cancer cell lines, further finding that this occurred by a mixture of transcriptional and mRNA stability mechanisms (see S3 Fig and related text). In both scenarios, nutrient deprivation caused destabilization of the microtubule cytoskeleton (S3D and 3H Fig). We conclude that various physiological and damaging inputs change tubulin gene expression either transcriptionally, thus affecting the microtubule cytoskeleton, or post-transcriptionally through changes in microtubule dynamics.

### PI3K activity increases tubulin gene expression via microtubule stability and autoregulation

**Fig 4:**
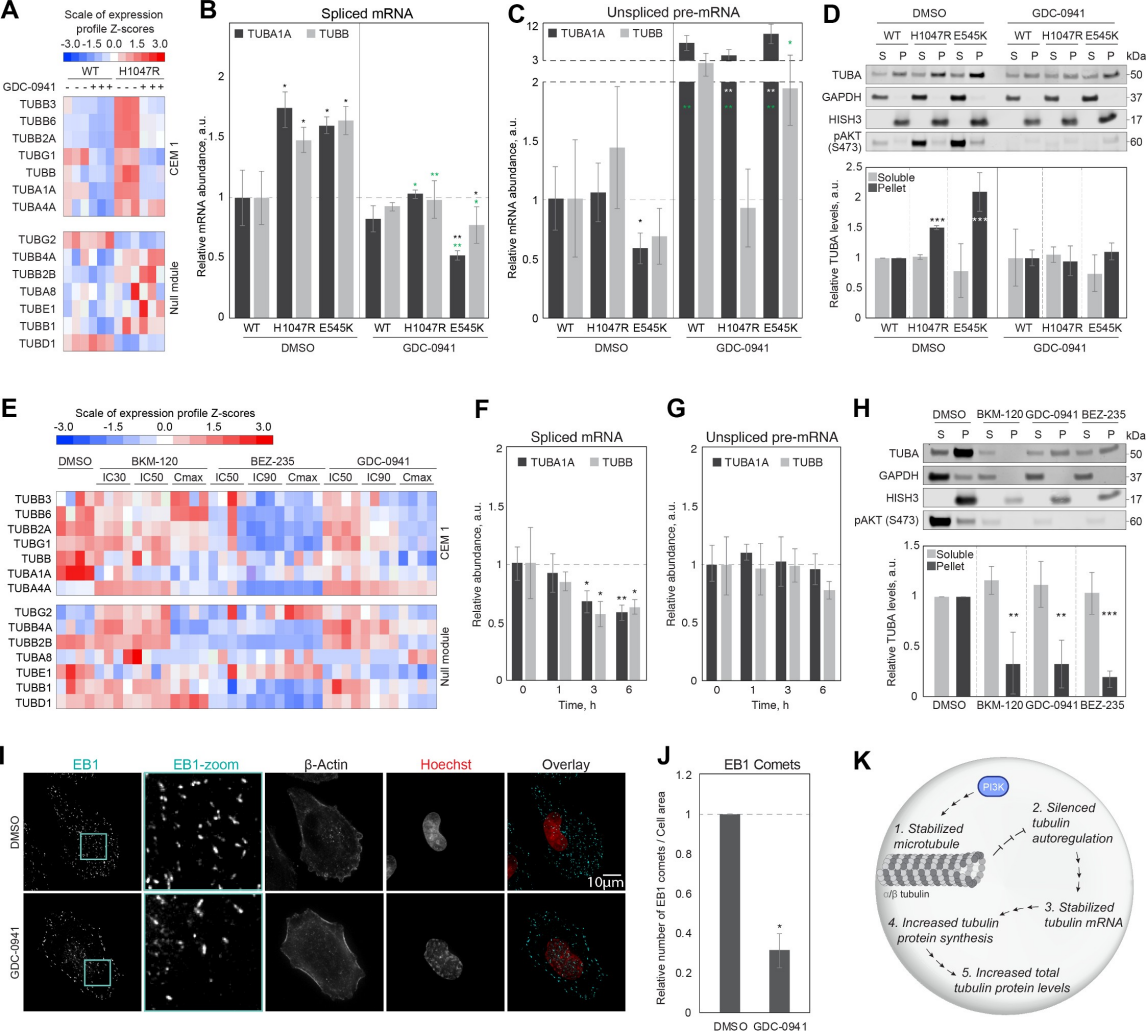
PI3K activity increases tubulin gene expression through autoregulation. (A) DGE in parental and cells expressing constitutively active PI3K H1047R mutant cells, treated with DMSO or indicated PI3K inhibitors for 4 h. (B and C) Relative expression of TUBA1A and TUBB unspliced pre-mRNA (B) and spliced TUBA1A and TUBB mRNA (C) in parental and PI3K mutant cell lines H1047R and E545K, treated with DMSO control or 1 μM GDC-0941 for 4 h. All the relative gene-expression data are normalized to housekeeping gene GAPDH or RPL19 and to DMSO-treated parental cells. (D) Tubulin partitioning to unpolymerized (soluble, S) and polymerized (P) normalized to loading control (GAPDH for S; HISH3 for P) and to DMSO-treated parental cells in parental and cells expressing constitutively active PI3K mutants H1047R and E545K, treated with DMSO or 1 μM GDC-0945 for 4 h. (E) DGE in A2058 cells treated with DMSO control or the indicated concentrations of PI3K inhibitors for 6 h (GEO series GSE66343 [28]). (F–G) RT-qPCR in A2058 cells treated with 1 μM GDC-0941 for indicated periods of time (*x*-axes). (H) Tubulin partitioning to unpolymerized (S) and polymerized (P) normalized to loading control (GAPDH for S; HISH3 for P) and to DMSO-treated cells in control and cells treated for 6 h with indicated 1 μM PI3K inhibitors. (I) Representative immunofluorescence images of control and cells treated with 1 μM GDC-0941 for 6 h and stained with anti-EB1 antibody (cyan), anti-β-actin, and Hoechst (red). (J) Relative number of detected EB1 comets per cell area in DMSO control and A2058 cells treated with 1 μM GDC-0941 for 6 h, normalized to DMSO-treated cells (100–200 cells). (K) A proposed model describes the mechanism through which PI3K signaling regulates tubulin gene expression through modification of tubulin autoregulation. Bar plots in all panels represent average values, and error bars standard deviations from three independent biological replicates. *p < 0.05, **p < 0.01, ***p < 0.001 in paired Student *t* test, in all panels. Black and white asterisk represent statistical significance relative to DMSO-treated parental cell line, green asterisks represent statistical significance relative to DMSO-treated control of the same cell line. a.u., arbitrary unit; DGE, differential gene expression; EB1, end-binding protein 1; GAPDH, Glyceraldehyde 3-phosphate dehydrogenase; GEO, Gene Expression Omnibus; HISH3, histone H3; pAKT, phospho-AKT; PI3K, phosphatidylinositol-4,5-biphosphate 3-kinase; RPL19, ribosomal protein L19; RT-qPCR, reverse-transcription quantitative PCR; TUBA, α-tubulin; TUBB, β-tubulin; TUBD, δ-tubulin; TUBE, ε-tubulin; TUBG, γ-tubulin; WT, wild type.

Multiple investigations of PI3K signaling scored highly in our bioinformatics search, including studies in which genetics were used to up-regulate the pathway activity and small-molecule inhibitors to down-regulate it. In each case, PI3K pathway activation correlated with an increase in tubulin mRNA levels (GEO GSE17785) and pathway inhibition with a decrease [28] (Table 1, S1 Table). One CLIC report suggested that MCF10A cells that expressed constitutively active PI3K with a mutation in the kinase domain [29] (H1047R) had increased tubulin mRNA levels (GEO GSE17785, Fig 4A). PI3K inhibition with GDC-0941 prevented this up-regulation of tubulin mRNA levels, suggesting involvement of PI3K in concerted regulation of tubulin expression (Fig 4A). Using RT-qPCR, we found that the levels of spliced TUBA1A and TUBB mRNAs (Fig 4B) were significantly higher in H1047R cells, suggesting post-transcriptional regulation of tubulin mRNA. In support of this result, transcription rate of TUBA1A and TUBB in H1047R cells was unchanged compared to the parental cell line (Fig 4C). To directly test whether PI3K was involved in the regulation of tubulin mRNA stability, we measured tubulin mRNA levels in parental and H1047R-mutant cells treated with the PI3K inhibitor GDC-0941 [28]. Upon PI3K inhibition in parental cell line, the stability of tubulin mRNAs remained unchanged (Fig 4B) despite the increased transcription rate (Fig 4C). Importantly, PI3K inhibition restored normal levels of spliced TUBA1A and TUBB mRNA in H1047R-mutant cell line, comparable to the parental cell line (Fig 4B), while it increased the transcription of TUBA1A, but not TUBB mRNA (Fig 4C). To test whether these results were more general, we measured TUBA1A and TUBB gene expression in another cell line carrying an activating mutation in the helical domain of PI3K (E545K [29]). We found that the levels of spliced TUBA1A and TUBB mRNA were significantly higher in E545K compared to parental cells (Fig 4B), while the rate of TUBA1A and TUBB transcription remained unchanged (Fig 4C), again consistent with regulation by mRNA stability. Treatment of E545K cells with GDC-0941 PI3K inhibitor restored normal levels of spliced TUBA1A and TUBB mRNA (Fig 4B), suggesting that PI3K activity regulated tubulin mRNA stability. As in parental and H1047R cells, transcription of TUBA1A and TUBB was higher in E545K mutant cells treated with GDC-0941 compared to control-treated cells, suggesting transcriptional activation triggered by PI3K inhibition (Fig 4C). We conclude that, despite activating transcription, PI3K inhibition post-transcriptionally suppresses tubulin mRNA levels, presumably through autoregulation.

We next sought to determine whether PI3K activity regulated the stability of tubulin mRNAs via changes in microtubule stability and altered ratio of soluble tubulin dimer and polymer. To test whether PI3K activation causes a decrease in the concentration of soluble tubulin dimer, we performed biochemical tubulin partitioning on parental, H1047R, and E545K cells. We found that both H1047R and E545K cell lines had less soluble tubulin dimer (Fig 4D; note the differences in fractions “S” across the different cell lines) and more microtubule polymer than the parental cell line (Fig 4D; note the differences in fractions “P” across the different cell lines). PI3K inhibition in these cells increased levels of soluble tubulin dimer and decreased microtubule polymer, comparable to the levels observed in parental cells (Fig 4D; note the differences in fractions “S” and “P” between cells treated with DMSO and GDC-0941). These data are consistent with a model in which PI3K activity increases microtubule stability, leading to a decrease in soluble tubulin and an increase in tubulin mRNA stability via the autoregulation pathway. PI3K inhibition with a small molecule reverses these changes. While these data do not reveal the mechanism by which PI3K regulates microtubule stability, they are consistent with published models [30].

To generalize our findings, we investigated a second cell line, A2058, in which our bioinformatic analysis revealed down-regulation of tubulin mRNAs upon PI3K inhibition. PI3K is constitutively active in these cells (Fig 4H). We treated A2058 cells with the PI3K inhibitors BKM-120 [31] (inhibitor for p110α/β/δ/γ), BEZ-235 [32] (a dual PI3K and mammalian Target Of Rapamycin (mTOR) inhibitor for p110α/γ/δ/β), or GDC-0941 [33] (inhibitor for PI3Kα/δ). In each case, drug treatment decreased the levels of tubulin mRNA (Fig 4E). Performing RT-qPCR on cells treated with 1 μM GDC-0941 (IC90 [28]), we found that PI3K inhibition post-transcriptionally down-regulated spliced tubulin mRNAs (Fig 4F) while leaving intact the transcription rate of tubulin genes (Fig 4G), consistent with regulation through mRNA stability. Biochemical partitioning of tubulin revealed that treatment with 1 μM BKM-120, BEZ-235, or GDC-0941 indeed caused an increase in levels of soluble tubulin dimer and a reduction of microtubule polymer (Fig 4H). To further confirm microtubule destabilization upon PI3K inhibition, we used immunofluorescence-based microscopy. We fixed and stained control and PI3K-inhibited cells using anti-EB1 antibody and counted the number of EB1-positive microtubule plus-tips per cell area as a readout for growing microtubules. Our data showed significant reduction in the number of growing microtubules in cells treated with 1 μM GDC-0941 compared to control-treated cells (Fig 4I and 4J).

Several studies have identified off-target destabilization of microtubules by kinase inhibitors, so it was important to test for this possible artifact. In RPE1 hTert cells, with low basal PI3K activity, we did not observe changes in the number of EB1-positive microtubule plus-tips upon PI3K inhibition with GDC-0941 or BEZ-235, suggesting the absence of off-target activity on tubulin (S4A and S4B Fig). Consistent with previous reports of off-target effects on microtubules [34], high dose of PI3K inhibitor BKM-120 caused a reduction in the number of EB1-positive microtubule plus-tips (S4A and S4B Fig).

Taken together, we conclude that PI3K activity positively regulates tubulin levels in cancer cells via microtubule stabilization, which lowers the concentration of soluble tubulin dimer, thus increasing tubulin mRNA stability through the autoregulation pathway. Our findings show for the first time, to our knowledge, that tubulin autoregulation via mRNA stability mediates changes in total tubulin concentration triggered by an important signaling pathway. PI3K inhibition in cell lines with low constitutive kinase activity did not perturb microtubules (Fig 4D, S4A and S4B Fig), suggesting that this regulation is selective for cancer cells and normal physiological states in which PI3K is strongly activated.

## Conclusions

Our data show that quiescent cells can detect both subtle and strong perturbation of microtubule dynamics and mount robust gene-expression responses. Moreover, our study is the first to tease apart the opposite effects of microtubule destabilization versus stabilization on gene expression from their similar effects on mitotic arrest. Our findings begin to validate the idea that an interphase “Microtubule Integrity Response” (MIR) pathway exists. Tubulin genes were the most responsive, especially to mild microtubule perturbation. Our RT-qPCR data confirm that this regulation involves a previously reported pathway, tubulin autoregulation, in which control occurs at the level of mRNA stability, not transcription. The molecular mechanism of tubulin autoregulation is not known, but it is thought to involve cotranslational degradation of tubulin mRNAs by a pathway that is sensitive to the concentration of soluble tubulin dimer [14,15].

Because of limitations in available technology at the time of discovery, early studies of tubulin autoregulation could only measure total TUBA and TUBB mRNA regulation and not the regulation of specific isoforms [13–15]. Our study is the first to show that autoregulation extends to all the expressed TUBA and TUBB genes and TUBGs. Curiously, we reveal that autoregulation controls tubulin pseudogenes despite their lost functionality. In recent years, pseudogenes have come in the spotlight as potential regulators of their functional counterparts [35]. It is possible that tubulin pseudogenes may still have retained some functionality that must be tightly controlled. Understanding the molecular mechanism and physiological relevance of tubulin autoregulation will be important for the discovery and characterization of the hypothetical MIR.

Prior work on tubulin autoregulation was confined to microtubule-drug–induced effects in a few cell types in culture [14,36,37]. Using bioinformatics to query public data sets, we provide evidence that coregulation of expressed TUBAs, TUBBs, and TUBGs is observed in response to stimuli other than microtubule-targeting drugs, in tissues, and in cycling and quiescent cell cultures. Moreover, we show for the first time that this pathway is part of concerted cellular responses to many different stimuli. In the case of PI3K signaling, we find that activation of the kinase stabilizes microtubules, thus decreasing the pool of soluble tubulin dimer and stabilizing tubulin mRNA. This is consistent with the proposed mechanism of tubulin autoregulation. Our findings, therefore, demonstrate the ubiquity and importance of autoregulation in controlling tubulin gene expression and very likely microtubule biology in general.

### Autoregulation suggests functions of tubulin beyond building microtubules

A striking feature of our DGE data in quiescent RPE1 hTert cells, which was also seen in two large GEO studies that we reanalyzed, is a lack of coregulation of other microtubule components with tubulin: tubulin autoregulation signature extends to all expressed tubulin but not to microtubule-associated, plus-tip–binding, or motor proteins, nor are these components coregulated on the transcriptional level in response to microtubule damage. This finding is quite different from coordinated gene expression in other biological responses; for example, many of the genes required to build lysosomes [38] or mitochondria [39] are coordinately regulated. The specific regulation of all expressed tubulins, but not other microtubule proteins, suggests that cells care more about the concentration of tubulin than other components of the microtubule cytoskeleton. Perhaps this simply reflects the central role of tubulin in building microtubules. A related puzzle is that autoregulation is counterhomeostatic from the perspective of microtubule mass, at least in response to microtubule drug perturbation. CA4 treatment reduces microtubule mass, yet cells respond by destabilizing tubulin mRNA and reducing protein levels, which would seem to exacerbate the problem, not correct it. The converse is true for PTX. An intriguing possible explanation for both the apparent paradoxes is that soluble tubulin dimer has some function other than building microtubules that necessitates tight control of its concentration. For example, soluble tubulin was proposed to gate Voltage-Dependent Anion Channels (VDACs) in mitochondria [40], and it is conceivable that autoregulation evolved to regulate this function, not microtubule assembly. Another possibility is that autoregulation evolved to balance the synthesis of TUBA and TUBB subunits, in which case other components of the microtubule cytoskeleton are irrelevant. But if this is the case, why is TUBG1 also coregulated? These are speculations, but they point out that we truly do not understand the adaptive benefit of tubulin autoregulation despite its frequent occurrence in cellular physiology, as revealed by our bioinformatics analysis.

### Discovery of physiological processes that change microtubule stability from transcriptomic data

Research in the microtubule field has been driven largely by microscopy and biochemistry, while transcriptomic approaches have been neglected. Our CLIC search shows that bioinformatic mining of public data sets can be used as a starting point for discovery. As a positive control, this approach returned multiple studies of microtubule-perturbing agents, such as PTX and bisphenol-A. Unexpectedly, we uncovered many more previously unreported conditions that perturb tubulin gene expression that remain to be investigated.

Our mechanistic follow-up study showed that PI3K activity promotes stabilization of tubulin mRNA. This effect is achieved through the microtubule-stabilizing activity of PI3K signaling and tubulin autoregulation. Notably, the effect of autoregulation on basal tubulin levels, and on response to PI3K inhibitors, was stronger in cells carrying an activating mutation in PI3K. The inhibition of PI3K in wild-type and H1047R and E545K mutant MCF10A cells dramatically increased the transcription of TUBA1A and TUBB through mechanisms that remain to be explored. Suppression of TUBA1A- and TUBB-spliced mRNA stability through tubulin autoregulation counteracts the increased transcription rate, restoring normal levels of tubulin biosynthesis. Activating mutations in PI3K cause many changes in growth and metabolism in cancer cells [41]. Our data add change in tubulin levels to that picture, which could be significant for successful mitosis, response to microtubule drugs, and metastasis pathways regulated by microtubules.

Our genome-scale data analysis and PI3K investigation point to the importance of tubulin autoregulation in normal biology and cancer pathophysiology. Tubulin autoregulation was a central concern in the microtubule field in the 1980s, but it was abandoned as the field moved towards biophysical directions in the 2000s. We feel it is now important to investigate the full molecular basis of the autoregulation pathway and extend the pioneering work on the role of the N-terminal peptide of nascent TUBB [15].

## Materials and methods

### Cell culture and drug treatments

All the cell lines used in this study were grown at 37°C with 5% CO_2_ in a humidified incubator. hTert-RPE1-eGFP-EB3 (a kind gift from D. Pellman, HMS, Boston, MA, USA), and hTert-RPE1 (ATCC, Manassas, VA, USA) cell lines were grown in Dulbecco's modified medium (nutrient mixture F12, DMEM/F12) supplemented with 10% FBS and 1% (vol/vol) penicillin/streptomycin (pen/strep). Parental and PI3 kinase mutant MCF10A cell lines, H1047R and E545K (a gift from J. Brugge, HMS), were grown as previously described [29]. Prior to drug treatment, parental and PI3K mutant MCF10A cell lines, H1047R and E545K, were washed 2 times with 1× PBS and grown in medium lacking EGR [29]. Cells were incubated overnight. A2058 cells (a kind gift from P. Sorger, HMS), U2OS cells, and A549 cells (ATCC) were grown in DMEM supplemented with 10% FBS and 1% (vol/vol) pen/strep. Microtubule drugs were used at 1 nM or 100 nM for CA4 or 3 nM or 300 nM for PTX for 6 and 24 h. PI3K inhibitors BKM-120, BEZ-235, and GDC-0941 (a gift from Nathanael Gray, HMS) were administered at 1 μM. All the microtubule poisons and PI3K inhibitors were dissolved in DMSO, and 0.01% DMSO was used as vehicle control. D-glucose–free medium was prepared using D-glucose–free DMEM (Thermo Fisher Scientific, Waltham, MA, USA) supplemented with 10% dialyzed FBS (Thermo Fisher Scientific), and 1% vol/vol pen/strep (Thermo Fisher Scientific). Media with D-glucose were prepared by adding D-glucose (Sigma Aldrich, St. Louis, MO, USA) to the glucose-free medium at 4.5 g/l final concentration. L-glutamine–free medium was prepared using L-glutamine–and sodium-pyruvate–free DMEM (Corning, Corning, NY, USA) supplemented with 10% dialyzed FBS, 1% sodium pyruvate (Corning), and 1% vol/vol pen/strep. Media with L-glutamine were prepared by adding L-glutamine (Corning) to the L-glutamine–free medium at 2 mM final concentration. For D-glucose and L-glutamine deprivation, standard growth medium was removed, cells were washed 3 times with 1× PBS, and media with (control) or without D-glucose or L-glutamine were added to cells.

### Flow cytometry cell-cycle profiling

In the flow cytometry validation experiments, RPE1 hTert cells were grown per treatment condition on 10-cm petri dishes for 1 day for cycling culture and 5 days after reaching full confluency for quiescent cultures. Cells were then treated with DMSO control, 100 nM CA4, or 300 nM PTX for 6 h. For each condition, cells were detached from the dish in 0.05% Trypsin solution, resuspended in 1× PBS supplemented with 1% FBS and spun down by centrifugation (400 × *g* for 5 min), followed by dispersion of the pellet into a single-cell suspension in 1× PBS. Cells were then fixed in 4% PFA for 20 min on ice, stained with DAPI (#D1306; Invitrogen, Carlsbad, CA, USA), and analyzed on a BD LSRII flow cytometer (BD Biosciences, Franklin Lakes, NJ, USA). The dye was excited with a 405-nm laser, and emitted fluorescence was detected with a 450/50 bandpass filter. For data analysis, FlowJo v.X.0.7 and the built-in cell-cycle quantification platform were used (the univariate model without any adjustments).

### RNA-seq and data analysis

Total RNA was collected and purified using the PureLink RNA Mini Kit (Invitrogen, Thermo Fisher Scientific). RNA concentration and quality were determined using NanoDrop and Bioanalyzer, respectively, and 500 ng of purified RNA was used as input for the Illumina TruSeq Stranded mRNA Library Prep Kit (Illumina, San Diego, CA, USA). Barcoded libraries were pooled and sequenced by The Bauer Core Facility at Harvard University, where the pooled library was quantitated using KAPA and single-end sequenced on an Illumina NextSeq (Illumina). RNA-seq reads were mapped using STAR [42] (version 2.1.0j) and processed using HTSeq-count [43] (version 0.6.1). GRCh38 reference genome and transcript annotations were used for gene mapping; Entrez Gene identifiers and the org.Hs.eg.db database were used for genome-wide annotation [44]. DGE and statistical analysis were performed using the edgeR package [45]. Genes with >50 CPM and a fold change significantly different from zero in Wilcoxon signed-rank test (*p* < 0.05) were marked as differentially expressed genes, based on three biological replicates. EnrichR was used for GSEA [46,47], and gene sets with Benjamini–Hochberg adjusted *p*-values *p* < 0.01 were considered statistically significant.

### Microarray data analysis

Publicly available microarray data were read, normalized, and analyzed using linear models and empirical Bayes methods for assessing differential expression (limma, [48]). The microarray probes that hybridize with tubulin genes were extracted, and heatmaps of differential expression were plotted using R.

### Bioinformatic analysis

We used the CLIC-gene online tool for bioinformatic analysis of tubulin expression correlation [26]. Querying a list of all tubulin genes against human and mouse data set platforms, the algorithm calculated average Pearson expression correlation for each data set within one platform separately and using expression correlation coefficients obtained from the top-ranked 417 human and 122 mouse DGE data sets. The 417 human data sets with high tubulin expression correlation were then manually annotated.

### RT-qPCR

Reverse transcription was performed from 500 ng of purified total RNA, using SuperScript IV (Invitrogen) and random hexamer primers, according to the manufacturer’s protocol. RT-qPCR reaction was performed using 5 ng of cDNA and 2× SYBRGreen master mix (Thermo Fisher Scientific) on a BioRad thermocycler (BioRad, Hercules, CA, USA). For each reference and gene of interest, two sets of primers were designed: one set of primers amplified specifically unspliced pre-mRNA, while the other set of primers amplified specifically spliced mRNA. Primer sequences are listed in S1 Table. All primer pairs were validated by PCR, followed by gel electrophoresis. PCR products were further submitted to Sanger sequencing and subsequently mapped against the genome, confirming correct product amplification. RT-qPCR data analysis was performed using the ddCt method [49]. Statistical significance was determined using two-tailed paired Student *t* test and *p* < 0.05.

### Western blotting and tubulin partitioning

Whole-cell extracts for immunoblot analysis were prepared by cell lysis in 2× SDS sample buffer containing 50 mM Tris-HCl (pH 6.8), 2% SDS, 10% glycerol, 1% β-mercaptoethanol, 12.5 mM EDTA, and 0.02% bromophenol blue in water. Samples were denatured at 100°C for 10 min prior to SDS-PAGE gel electrophoresis on 1.5 mm NuPAGE Novex 4%–12% Bis-Tris Protein Gels (Thermo Fisher Scientific), using the Mini Trans-Blot Cell system (BioRad).

For tubulin partitioning, cells were grown in 12-well dishes to 70%–80% confluence. Prior to lysis, cells were washed 1× with 1× PBS prewarmed to 37°C, and PBS was aspirated. Unpolymerized tubulin was extracted in 300 μl of tubulin extraction buffer containing 60 mM PIPES (pH 6.8), 25 mM HEPES (pH 7.2), 10 mM EGTA (pH 7–8), 2 mM MgCl_2_, 0.5% TritonX-100, 10 μM PTX (Sigma Aldrich), and a protease inhibitor tablet (Roche, Branchburg, NJ, USA) for 2 min at room temperature. Extraction buffer with solubilized protein was collected and mixed with 100 μl 4× SDS sample buffer. The remaining material, containing polymerized tubulin, was then lysed with 400 μl 1× SDS sample buffer. Samples were denatured at 100°C for 10 min. Prior to SDS-PAGE gel electrophoresis, equal aliquots of samples were concentrated 2× by evaporation.

For all immunoblots, Precision Plus Protein Dual Color Standard ladder was used (BioRad). Dilutions and primary antibodies used: 1:15,000 GAPDH (#2118; Cell Signaling Technology, Danvers, MA, USA), 1:10,000 TUBA (DM1alpha, #05–829; MilliporeSigma, Burlington, MA, USA), 1:10,000 Histone H3 (#4499; Cell Signaling Technology), and 1:500 phospho-Akt S437 (#9271; Cell Signaling Technology). The following secondary antibodies were used: 1:15,000 conjugated goat anti-mouse DyLight 680 (#35518; Thermo Fisher Scientific) and 1:15,000 conjugated goat anti-rabbit DyLight 800 (#35571; Thermo Fisher Scientific). All the blots were imaged and analyzed using The Odyssey Infra-Red Imaging System (LI-COR, Lincoln, NE, USA) equipped with Image Studio software. Statistical analysis and data plotting were performed in Microsoft Excel.

### Quantitative immunofluorescence

For quantitative immunofluorescence, cells were seeded and grown on coverslips overnight, prior to drug treatment. For immunostaining of EB1, cells were washed once with 1× PBS at 37°C and fixed in cold methanol at ^l20°C for 20 min. Cells were then permeabilized in 4% paraformaldehyde in PBS at room temperature for 20 min. After blocking in 2% bovine serum albumin in PBS, primary antibodies were incubated at room temperatures for 1 h. The following primary antibodies and dilutions were used: 1:500 mouse anti-EB1 (#2164; Cell SignalingTechnology), 1:1,000 rabbit anti-β-actin (#4970; Cell Signaling Technology), 1:15,000 Hoechst 3342 (#H3570; Thermo Fisher Scientific). Cells were then stained with secondary antibodies at room temperature for 30 min. The following secondary antibodies and dilutions were used: 1:400 Alexa Fluor goat anti-rabbit IgG (#11008; Life Technologies, Carlsbad, CA, USA), and 1:400 Alexa Fluor goat anti-mouse IgG (#11001; Life Technologies). Three-dimensional image stacks of interphase cells were acquired in 0.2-μm steps using a 60× NA 1.42 objective on an Olympus DeltaVision microscope (GE Healthcare, Chicago, IL, USA) equipped with a DAPI/FITC/TRITC/CY5 filter set (Chroma, Bellows Falls, VT, USA), and an sCMOS 5.5 camera (PCO, Kelheim, Germany). EB1 comet counting and cell area measurements were performed on maximum-intensity z-stack projections using a custom-made ImageJ macro, which is available upon request. Statistical analysis and data plotting were performed in R.

### Statistical analysis

For gene-expression profiling, statistical analysis was supplied as part of the edgeR package and performed according to the user manual. For tubulin gene-expression correlation, statistical analysis was performed using the online CLIC-gene tool. All the Student *t* test analyses were performed considering two-tailed distribution and two degrees of freedom.

## Supporting information

S1 Fig Subthreshold and saturating effect of microtubule drugs on microtubules in quiescent RPE1 hTert cells. (A) Cell-cycle profiles of cycling and quiescent RPE1 hTert cells treated with DMSO or microtubule drugs (*x*-axis). (B and C) Biochemical partitioning of tubulin into soluble (S) and polymerized (P) in quiescent RPE1 hTert cells treated with DMSO control and microtubule drugs at indicated concentrations for 6 (B) and 24 h (C). Each soluble tubulin fraction is normalized to the housekeeping gene GAPDH, and each polymerized fraction to the housekeeping gene HISH3. All data are normalized to DMSO-treated control cells. Plotted are average values from 3 biological replicates. (D) Average number of EB1-positive microtubule plus-tips per cell area in cells treated with microtubule drugs at indicated concentrations (*x*-axis) and normalized to DMSO-treated control cells (>100 cells per condition). (E) Representative immunofluorescence images of control and cells treated with indicated microtubule drugs for 24 h and stained with anti-EB1 antibody (cyan), anti-β-actin, and Hoechst (red). Bar plots in all panels represent average values and error bars standard deviations from three independent biological replicates. *p-value < 0.05, **p-value < 0.01, ***p-value < 0.001 in paired Student *t* test compared to DMSO control. EB1, end-binding protein 1; GAPDH, Glyceraldehyde 3-phosphate dehydrogenase; HISH3, histone H3; hTert, human telomerase reverse transcriptase; RPE1, retinal pigment epithelial 1
(TIF)

S2 Fig Microtubule damage triggers differential tubulin gene expression across many cancer cell types and in vivo. (A) Expression profiles of all detected TUBA and TUBB isoforms across a panel of breast, ovarian, and endometrial cancer cell lines treated with microtubule poisons for 24 h. This data set is available on the GEO database (GEO series GSE50811, GSE50830, and GSE50831 [16]). Dendrogram on the left represents Pearson distance between expression profiles. Each column of the heatmap represents DGE in one cell line treated with the indicated microtubule drug, marked above the heatmap. Each row represents a gene, labeled on the *y*-axis. Color key is depicted in upper left corner. Data are represented as Log_2_FC relative to DMSO control. (B) Expression profiles of all detected TUBA and TUBB isoforms in heart endothelial cells isolated from control and rats treated with microtubule poisons VIN, VCR, or COL at indicated doses (*x*-axis) for 6 and 24 h. This data set is available on the GEO database (GEO series GSE19290). Dendrogram on the left represents Pearson distance between expression profiles. Each column of the heatmap represents DGE in one condition, labeled on the *x*-axis. Each row represents a gene, labeled on the *y*-axis. Color key is depicted in upper left corner. Data are represented as Log_2_FCs relative to DMSO control. (C) Experimental strategy and primer design for RT-qPCR. COL, colchicine; DGE, differential gene expression; GEO, Gene Expression Omnibus; GSE, gene set enrichment; Log_2_FC, Log_2_ Fold Change; RT-qPCR, reverse-transcription quantitative PCR; TUBA, α-tubulin; TUBB, β-tubulin; VCR, vincristine; VIN, vinblastine
(TIF)

S3 Fig Nutrient deprivation regulates the expression of TUBA and TUBB isoforms transcriptionally and post-transcriptionally. (A and E) DGE in control and cells deprived of D-glucose (A, A549 cell line, GEO series GSE56843 [27]), or L-glutamine (E, U2OS cell line, GEO series GSE59931). Each column represents DGE in one replicate of control or nutrient-deprived cells (*x*-axis). Each row represents one gene (*y*-axes). CEM1 consists of tubulin genes with high Pearson expression correlation. Null module consists of tubulin genes with low Pearson expression correlation. Scales of expression profile z-scores are depicted above each heatmap. (B and F) Relative expression of TUBA1A and TUBB unspliced pre-mRNA in control and A549 cells deprived of D-glucose (B) or control and U2OS cells deprived of L-glutamine for indicated periods of time (F, *x*-axes). (C and G) Relative expression of TUBA1A and TUBB mRNA in control and A549 cells deprived of D-glucose (C), or control and U2OS cells deprived of L-glutamine for indicated periods of time (G, *x*-axis). All the relative gene-expression data are normalized to housekeeping gene GAPDH or RPL19 and to time point 0 h (control). (D and H) Tubulin partitioning to unpolymerized (soluble) and polymerized (pellet) and total tubulin, normalized to loading controls: GAPDH for soluble and total and HISH3 for pellet in control and nutrient-deprived cells for 24 h. All data are normalized to DMSO control. Bar plots in all panels represent average values and error bars standard deviations from three independent biological replicates. *p < 0.05, **p < 0.01, ***p < 0.001 in paired Student *t* test compared to control treatment. CEM, coexpression module; DGE, differential gene expression; GAPDH, Glyceraldehyde 3-phosphate dehydrogenase; GEO, Gene Expression Omnibus; GSE, gene set enrichment; RPL19, ribosomal protein L19; TUBA, α-tubulin; TUBB, β-tubulin.
(TIF)

S4 Fig PI3K inhibitor BKM-120, but not BEZ-235 and GDC-1941, displays off-target effect on microtubules. (A) Quantification of the number of EB-positive microtubule plus-tips per cell area in RPE1 hTert cells treated with DMSO or indicated concentrations (*x*-axis) of PI3K inhibitors. Data are normalized to DMSO-treated control cells. Lines represent average number of EB signals per cell area, and shaded area represents standard error of the mean over three independent experiments and >100 cells per condition. (B) Representative immunofluorescence images of control and cells treated with indicated 1 μM PI3K inhibitors for 6 h, and stained with anti-EB1 antibody (cyan), anti-β-actin, and Hoechst (red). EB1, end-binding protein 1; hTert, human telomerase reverse transcriptase; PI3K, phosphatidylinositol-4,5-biphosphate 3-kinase; RPE1, retinal pigment epithelial 1
(TIF)

S1 Table RT-qPCR primer sequences. Listed are target mRNA species, sequences, and orientation of all the primers used for RT-qPCR in this study. RT-qPCR, reverse-transcription quantitative PCR
(DOCX)

S2 Table Perturbations that trigger tubulin differential expression. Listed are rank based on descending Pearson expression correlation coefficients, GEO identifiers, titles of studies, and observed Pearson expression correlation coefficients for a subset of tubulin genes across the 417 data sets from human platform in the CLIC report. CLIC, CLustering by Inferred Co-expression; GEO, Gene Expression Omnibus
(DOCX)

S1 Data Raw data used to create figures presented throughout the manuscript. Each spread sheet contains data from one or more panels of a figure, annotated in its name. Given are the measurements from all biological replicates performed.
(XLSX)

## Abbreviations

a.u.: arbitrary unit
CA4: combretastatin A-4
CEM: co-expressed modules
CLIC: CLustering by Inferred Co-expression
CPM: counts for each gene per million detected reads
DGE: differential gene expression
DMEM: Dulbecco’s modified medium
EB1: end-binding protein 1
ERB: eribulin
FDR: false discovery rate
GAPDH: Glyceraldehyde 3-phosphate dehydrogenase
GEO: Gene Expression Omnibus
GO: gene ontology
GSEA: gene set enrichment analysis
HISH3: histone H3
hTert: human telomerase reverse transcriptase
Log_2_FC: Log_2_ Fold Change
MAPK: mitogen-activated protein kinase
MIR: Microtubule Integrity Response
mTOR: mammalian Target Of Rapamycin
pAKT: phospho-AKT
pen/strep: penicillin/streptomycin
PI3K: phosphatidylinositol-4,5-biphosphate 3-kinase
polyA+: polyadenylated
PTX: paclitaxel
RNA-seq: RNA sequencing
RPE1: retinal pigment epithelial 1
RPL19: ribosomal protein L19
RT-qPCR: reverse-transcription quantitative PCR
SAC: Spindle Assembly Checkpoint
SRC1: Steroid Receptor Coactivator-1
TUBA: α-tubulin
TUBB: β-tubulin
TUBD: δ-tubulin
TUBE: ε-tubulin
TUBG: γ-tubulin
VDAC: Voltage-Dependent Anion Channels
