# Supplementary figures and images for: Tubulin mRNA stability is sensitive to change in microtubule dynamics caused by multiple physiological and toxic cues
Source: PLoS Biol. 2019 Apr 9;17(4):e3000225. doi: 10.1371/journal.pbio.3000225 (PMC6474637; doi:10.1371/journal.pbio.3000225)

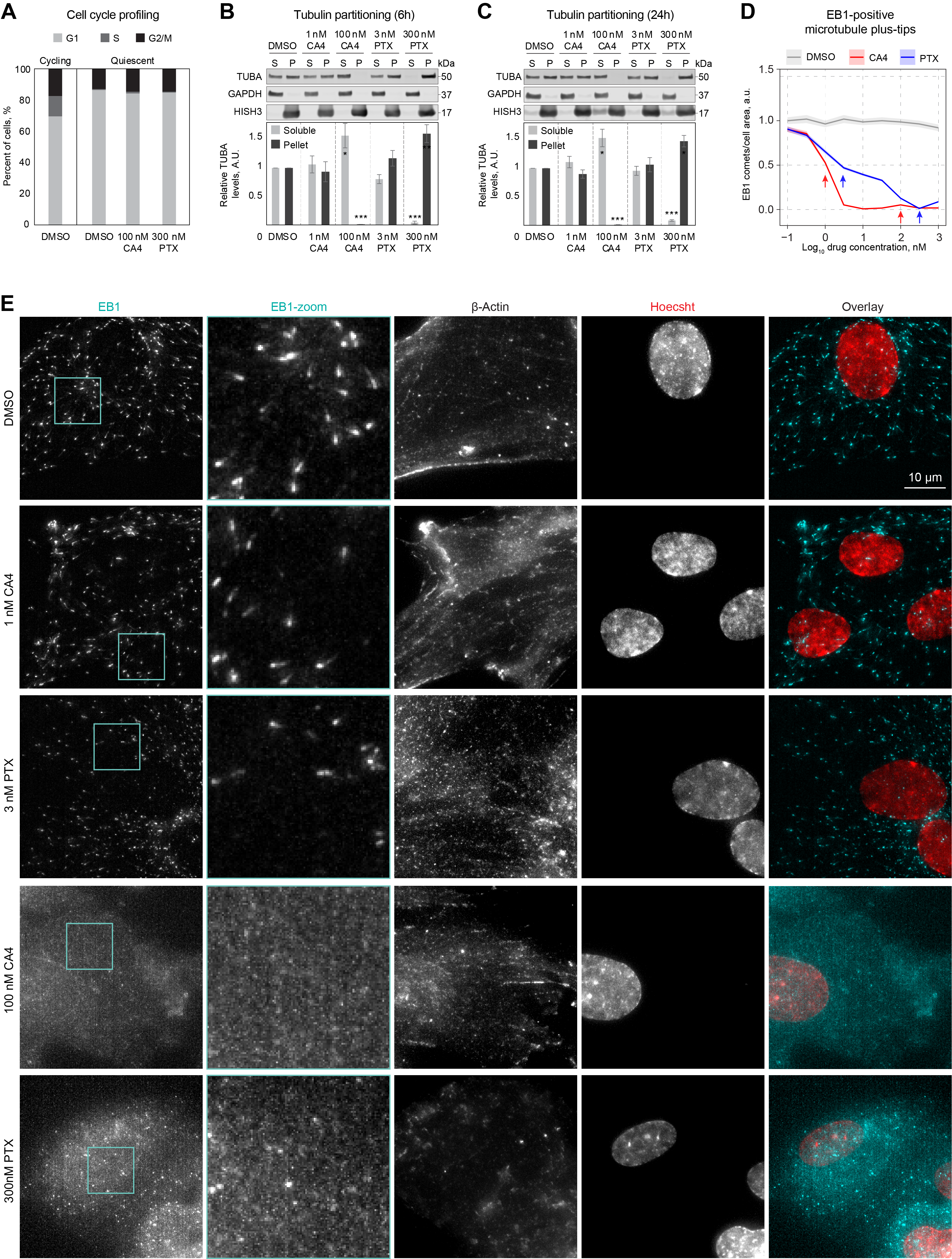

Supplement: S1 Fig — (A) Cell-cycle profiles of cycling and quiescent RPE1 hTert cells treated with DMSO or microtubule drugs (x-axis). (B and C) Biochemical partitioning of tubulin into soluble (S) and polymerized (P) in quiescent RPE1 hTert cells treated with DMSO control and microtubule drugs at indicated concentrations for 6 (B) and 24 h (C). Each soluble tubulin fraction is normalized to the housekeeping gene GAPDH, and each polymerized fraction to the housekeeping gene HISH3. All data are normalized to DMSO-treated control cells. Plotted are average values from 3 biological replicates. (D) Average number of EB1-positive microtubule plus-tips per cell area in cells treated with microtubule drugs at indicated concentrations (x-axis) and normalized to DMSO-treated control cells (>100 cells per condition). (E) Representative immunofluorescence images of control and cells treated with indicated microtubule drugs for 24 h and stained with anti-EB1 antibody (cyan), anti-β-actin, and Hoechst (red). Bar plots in all panels represent average values and error bars standard deviations from three independent biological replicates. *p-value < 0.05, **p-value < 0.01, ***p-value < 0.001 in paired Student t test compared to DMSO control. EB1, end-binding protein 1; GAPDH, Glyceraldehyde 3-phosphate dehydrogenase; HISH3, histone H3; hTert, human telomerase reverse transcriptase; RPE1, retinal pigment epithelial 1 (TIF) [file pbio.3000225.s001.tif]

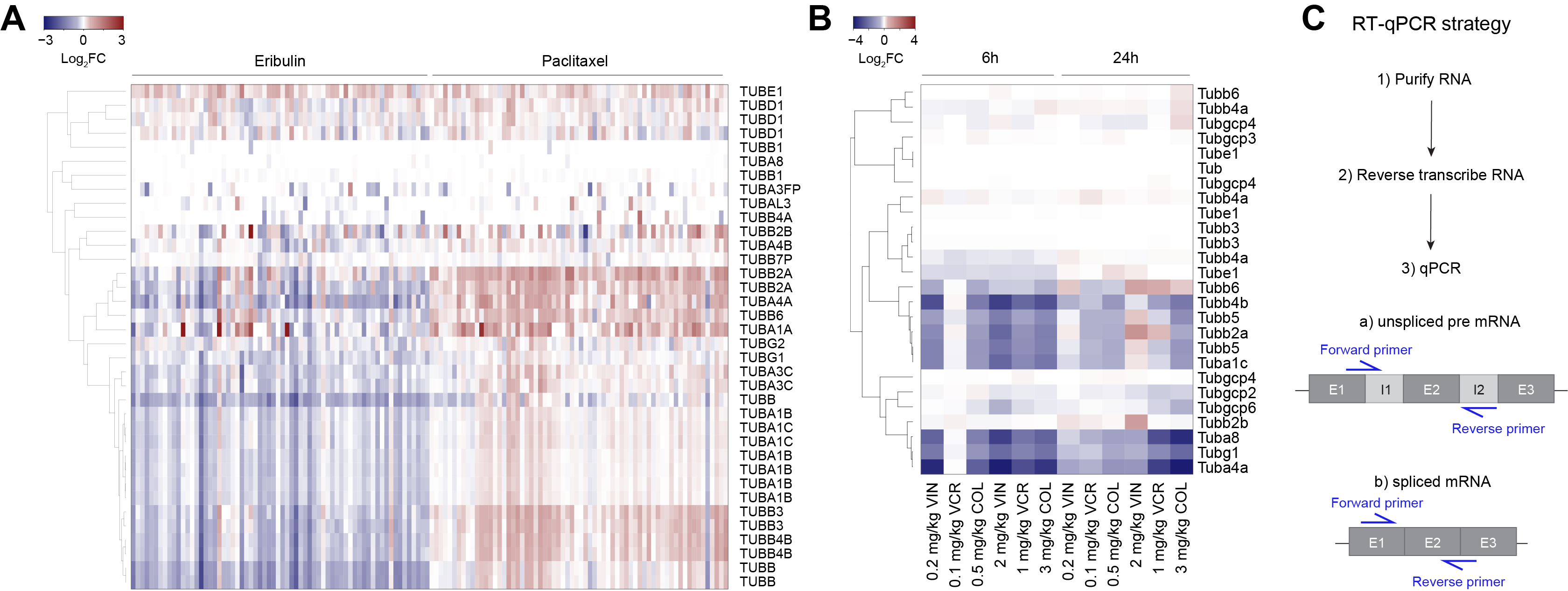

Supplement: S2 Fig — (A) Expression profiles of all detected TUBA and TUBB isoforms across a panel of breast, ovarian, and endometrial cancer cell lines treated with microtubule poisons for 24 h. This data set is available on the GEO database (GEO series GSE50811, GSE50830, and GSE50831 [16]). Dendrogram on the left represents Pearson distance between expression profiles. Each column of the heatmap represents DGE in one cell line treated with the indicated microtubule drug, marked above the heatmap. Each row represents a gene, labeled on the y-axis. Color key is depicted in upper left corner. Data are represented as Log2FC relative to DMSO control. (B) Expression profiles of all detected TUBA and TUBB isoforms in heart endothelial cells isolated from control and rats treated with microtubule poisons VIN, VCR, or COL at indicated doses (x-axis) for 6 and 24 h. This data set is available on the GEO database (GEO series GSE19290). Dendrogram on the left represents Pearson distance between expression profiles. Each column of the heatmap represents DGE in one condition, labeled on the x-axis. Each row represents a gene, labeled on the y-axis. Color key is depicted in upper left corner. Data are represented as Log2FCs relative to DMSO control. (C) Experimental strategy and primer design for RT-qPCR. COL, colchicine; DGE, differential gene expression; GEO, Gene Expression Omnibus; GSE, gene set enrichment; Log2FC, Log2 Fold Change; RT-qPCR, reverse-transcription quantitative PCR; TUBA, α-tubulin; TUBB, β-tubulin; VCR, vincristine; VIN, vinblastine (TIF) [file pbio.3000225.s002.tif]

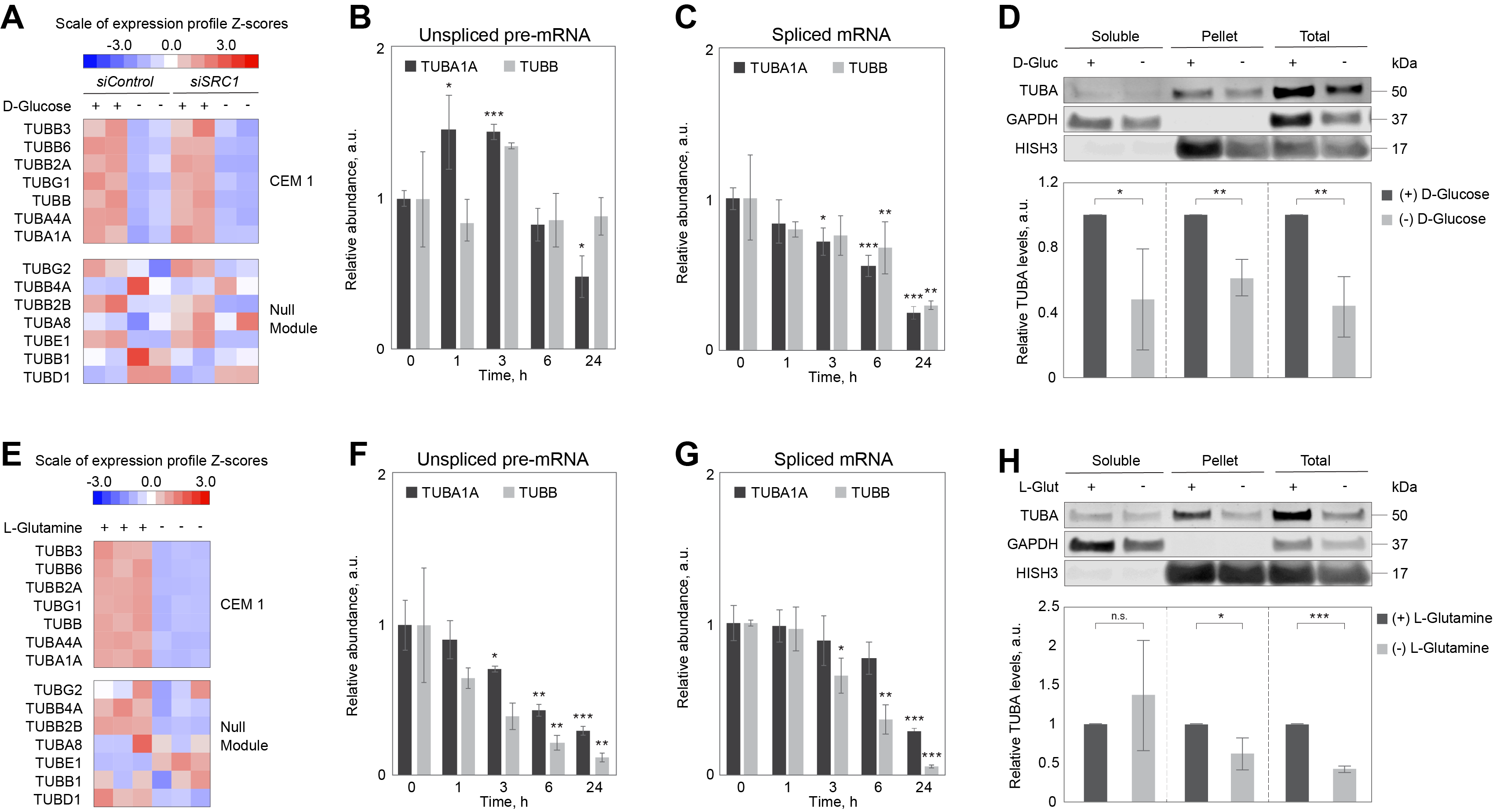

Supplement: S3 Fig — (A and E) DGE in control and cells deprived of D-glucose (A, A549 cell line, GEO series GSE56843 [27]), or L-glutamine (E, U2OS cell line, GEO series GSE59931). Each column represents DGE in one replicate of control or nutrient-deprived cells (x-axis). Each row represents one gene (y-axes). CEM1 consists of tubulin genes with high Pearson expression correlation. Null module consists of tubulin genes with low Pearson expression correlation. Scales of expression profile z-scores are depicted above each heatmap. (B and F) Relative expression of TUBA1A and TUBB unspliced pre-mRNA in control and A549 cells deprived of D-glucose (B) or control and U2OS cells deprived of L-glutamine for indicated periods of time (F, x-axes). (C and G) Relative expression of TUBA1A and TUBB mRNA in control and A549 cells deprived of D-glucose (C), or control and U2OS cells deprived of L-glutamine for indicated periods of time (G, x-axis). All the relative gene-expression data are normalized to housekeeping gene GAPDH or RPL19 and to time point 0 h (control). (D and H) Tubulin partitioning to unpolymerized (soluble) and polymerized (pellet) and total tubulin, normalized to loading controls: GAPDH for soluble and total and HISH3 for pellet in control and nutrient-deprived cells for 24 h. All data are normalized to DMSO control. Bar plots in all panels represent average values and error bars standard deviations from three independent biological replicates. *p < 0.05, **p < 0.01, ***p < 0.001 in paired Student t test compared to control treatment. CEM, coexpression module; DGE, differential gene expression; GAPDH, Glyceraldehyde 3-phosphate dehydrogenase; GEO, Gene Expression Omnibus; GSE, gene set enrichment; RPL19, ribosomal protein L19; TUBA, α-tubulin; TUBB, β-tubulin. (TIF) [file pbio.3000225.s003.tif]

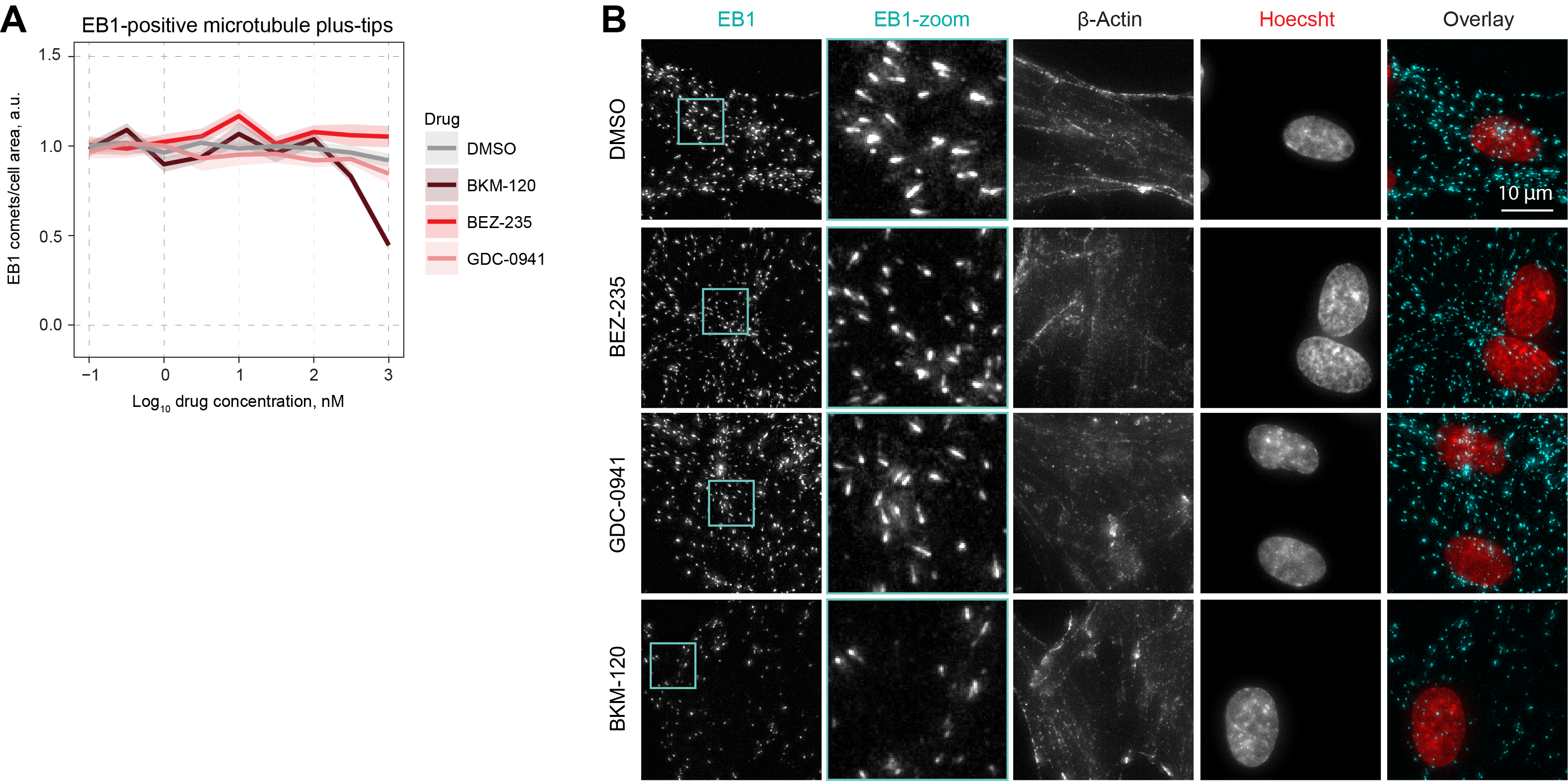

Supplement: S4 Fig — (A) Quantification of the number of EB-positive microtubule plus-tips per cell area in RPE1 hTert cells treated with DMSO or indicated concentrations (x-axis) of PI3K inhibitors. Data are normalized to DMSO-treated control cells. Lines represent average number of EB signals per cell area, and shaded area represents standard error of the mean over three independent experiments and >100 cells per condition. (B) Representative immunofluorescence images of control and cells treated with indicated 1 μM PI3K inhibitors for 6 h, and stained with anti-EB1 antibody (cyan), anti-β-actin, and Hoechst (red). EB1, end-binding protein 1; hTert, human telomerase reverse transcriptase; PI3K, phosphatidylinositol-4,5-biphosphate 3-kinase; RPE1, retinal pigment epithelial 1 (TIF) [file pbio.3000225.s004.tif]
