# Supplementary material for: Tubulin mRNA stability is sensitive to change in microtubule dynamics caused by multiple physiological and toxic cues
Source: PLoS Biol. 2019 Apr 9;17(4):e3000225. doi: 10.1371/journal.pbio.3000225 (PMC6474637; doi:10.1371/journal.pbio.3000225)
Supplement: S1 Table — Listed are target mRNA species, sequences, and orientation of all the primers used for RT-qPCR in this study. RT-qPCR, reverse-transcription quantitative PCR (DOCX) [file pbio.3000225.s005.docx]

| **Gene** | **RNA species** | **Orientation** | **Sequence, 5'-3'** |
| --- | --- | --- | --- |
| TUBA1A | pre-mRNA | forward | GCAGCATTTGTAGCAGGTGA |
|  |  | reverse | GCATTGCCAATCTGGACAC |
|  | mRNA | forward | CCACAGTCATTGATGAAGTTCG |
|  |  | reverse | GCTGTGGAAAACCAAGAAGC |
| TUBB | pre-mRNA | forward | CTGGACCGCATCTCTGTGTA |
|  |  | reverse | GGTTCACGAAAGGGACAAAA |
|  | mRNA | forward | GAAGCCACAGGTGGCAAATA |
|  |  | reverse | CGTACCACATCCAGGACAGA |
| GAPDH | pre-mRNA | forward | GGGAGGTAGAGGGGTGATGT |
|  |  | reverse | GAGGCAGGGATGATGTTCTG |
|  | mRNA | forward | AGCTCATTTCCTGGTATGACA |
|  |  | reverse | AGGGGAGATTCAGTGTGGTG |
| RPL19 | pre-mRNA | forward | TCCGAGAGGTGAAGGCATAG |
|  |  | reverse | GCCTCTTCTGAAGCCTGAGC |
|  | mRNA | forward | ATCGCCACATGTATCACAGC |
|  |  | reverse | TTGGTCTCTTCCTCCTTGGAT |
