# Supplementary material for: Tubulin mRNA stability is sensitive to change in microtubule dynamics caused by multiple physiological and toxic cues
Source: PLoS Biol. 2019 Apr 9;17(4):e3000225. doi: 10.1371/journal.pbio.3000225 (PMC6474637; doi:10.1371/journal.pbio.3000225)
Supplement: S2 Table — Listed are rank based on descending Pearson expression correlation coefficients, GEO identifiers, titles of studies, and observed Pearson expression correlation coefficients for a subset of tubulin genes across the 417 data sets from human platform in the CLIC report. CLIC, CLustering by Inferred Co-expression; GEO, Gene Expression Omnibus (DOCX) [file pbio.3000225.s006.docx]

|  |  |  | **Pearson Correlation Coefficient** | | | | | | | | |
| --- | --- | --- | --- | --- | --- | --- | --- | --- | --- | --- | --- |
| **Rank** | **GSE** | **Title of the study** | **TUBB3** | **TUBB6** | **TUBB2A** | **TUBG1** | **TUBB** | **TUBA4A** | **TUBA1A** | **TUBB4B** | **TUBA1C** |
| 1 | GSE59931 | Glutamine deprivation in U2OS cells | 0.98 | 0.99 | 0.99 | 0.99 | 0.99 | 0.98 | 0.99 | 0.99 | 0.91 |
| 2 | GSE36529 | Expression data from CtBP knockdown MCF-7 cells | 0.98 | 0.96 | 0.98 | 0.98 | 0.99 | 0.98 | 0.98 | 0.98 | 0.97 |
| 3 | GSE32158 | Bisphenol A Regulates the Expression of DNA Repair Genes in Human Breast Epithelial Cells (expression data) | 0.98 | 0.98 | 0.98 | 0.96 | 0.97 | 0.98 | 0.95 | 0.97 | 0.97 |
| 4 | GSE23952 | Expression data from TGF-beta treated Panc-1 pancreatic adenocarcinoma cell line | 0.98 | 0.98 | 0.97 | 0.97 | 0.92 | 0.98 | 0.98 | 0.98 | 0.98 |
| 5 | GSE22522 | Comparison of the transcriptome of K-LEC spheroids to control LEC spheroids | 0.98 | 0.96 | 0.96 | 0.98 | 0.97 | 0.92 | 0.97 | 0.98 | 0.96 |
| 6 | GSE56843 | Steroid Receptor Coactivator 1 is an Integrator of Glucose and NAD(+)/NADH Homeostasis | 0.93 | 0.97 | 0.97 | 0.96 | 0.97 | 0.98 | 0.95 | 0.97 | 0.96 |
| 7 | GSE20719 | Gene expression changes upon treatment of T47D breast cancer cells with the Pan-PI3 kinase inhibitor GDC-0941 | 0.95 | 0.96 | 0.97 | 0.98 | 0.94 | 0.96 | 0.93 | 0.98 | 0.93 |
| 8 | GSE4217 | Spheroid Formation and Recovery of Human Foreskin Fibroblasts at Ambient Temperature | 0.97 | 0.96 | 0.96 | 0.92 | 0.90 | 0.94 | 0.94 | 0.97 | 0.96 |
| 9 | GSE58605 | Expression data from A549 cells infected by adenovirus not carrying virus associated sequences in the genome. | 0.94 | 0.92 | 0.94 | 0.94 | 0.93 | 0.95 | 0.96 | 0.96 | 0.57 |
| 10 | GSE46708 | CD24 targets | 0.96 | 0.96 | 0.95 | 0.90 | 0.91 | 0.93 | 0.92 | 0.96 | 0.95 |
| 11 | GSE35428 | Transcriptional profiling of clinically relevant SERMs and SERM/estradiol complexes in a cellular model of breast cancer | 0.95 | 0.95 | 0.92 | 0.95 | 0.92 | 0.93 | 0.91 | 0.96 | 0.95 |
| 12 | GSE7745 | Mapping of HNF4Œ± binding sites, acetylation of histone H3 and expression in Caco2 cells | 0.94 | 0.94 | 0.94 | 0.87 | 0.94 | 0.88 | 0.88 | 0.94 | 0.95 |
| 13 | GSE46924 | 27-Hydroxycholesterol links cholesterol and breast cancer pathophysiology. | 0.90 | 0.93 | 0.92 | 0.95 | 0.93 | 0.90 | 0.84 | 0.93 | 0.88 |
| 14 | GSE16659 | Expression data of HGF/cMET pathway in prostate cancer DU145 cell line | 0.94 | 0.94 | 0.80 | 0.92 | 0.92 | 0.92 | 0.88 | 0.84 | 0.31 |
| 15 | GSE52659 | Expression data from WEEV infected BE(2)-C/m cells | 0.92 | 0.88 | 0.94 | 0.91 | 0.94 | 0.87 | 0.79 | 0.83 | 0.87 |
| 16 | GSE10444 | gene expression levels in long-term cultures of human dental pulp stem cells | 0.83 | 0.88 | 0.90 | 0.92 | 0.93 | 0.89 | 0.86 | 0.94 | 0.81 |
| 17 | GSE4218 | Spheroid Formation and Recovery of Human T98G Glioma Cells at Ambient Temperature | 0.92 | 0.93 | 0.87 | 0.84 | 0.81 | 0.93 | 0.88 | 0.94 | 0.91 |
| 18 | GSE15499 | HDAC5 is a repressor of angiogenesis and determines the angiogenic gene expression pattern of endothelial cells | 0.90 | 0.84 | 0.86 | 0.93 | 0.94 | 0.82 | 0.91 | 0.92 | 0.86 |
| 19 | GSE33143 | Targeted disruption of the BCL9/beta-catenin complex in cancer | 0.91 | 0.87 | 0.89 | 0.89 | 0.87 | 0.90 | 0.81 | 0.83 | 0.16 |
| 20 | GSE42733 | Gene expression profile of Nurse-Like Cells (NLC) derived from chronic lymphocytic leukemia | 0.81 | 0.90 | 0.89 | 0.90 | 0.88 | 0.88 | 0.84 | 0.68 | 0.91 |
| 21 | GSE36085 | Regulation of Autophagy by VEGF-C axis in cancer | 0.91 | 0.87 | 0.90 | 0.90 | 0.86 | 0.92 | 0.72 | 0.91 | 0.78 |
| 22 | GSE43700 | Microarray analysis of IL-10 stimulated adherent peripheral blood mononuclear cells | 0.90 | 0.88 | 0.87 | 0.74 | 0.81 | 0.92 | 0.92 | 0.93 | 0.91 |
| 23 | GSE29625 | Human embryonic stem cells derived from embryos at different stages of development share similar transcription profiles | 0.90 | 0.86 | 0.91 | 0.83 | 0.88 | 0.89 | 0.78 | 0.93 | 0.83 |
| 24 | GSE12098 | Comparison of the migration profile of MSCs | 0.88 | 0.70 | 0.88 | 0.89 | 0.90 | 0.89 | 0.81 | 0.93 | 0.89 |
| 25 | GSE13378 | Exposure of squamous esophageal cell line HET-1A to deoxycholic acid (DCA) | 0.85 | 0.74 | 0.90 | 0.87 | 0.85 | 0.87 | 0.87 | 0.89 | -0.19 |
| 26 | GSE32161 | Microarray analysis of genes associated with cell surface NIS protein levels in breast cancer | 0.88 | 0.81 | 0.91 | 0.90 | 0.79 | 0.79 | 0.87 | 0.88 | 0.87 |
| 27 | GSE16070 | Networking of differentially expressed genes in human MCF7 breast cancer cells resistant to methotrexate | 0.90 | 0.90 | 0.79 | 0.77 | 0.83 | 0.84 | 0.90 | 0.92 | 0.73 |
| 28 | GSE36176 | Gene expression arrays on lung cancer cells exposed to Notch inhibitor | 0.88 | 0.85 | 0.90 | 0.90 | 0.71 | 0.90 | 0.71 | 0.92 | 0.87 |
| 29 | GSE23399 | Gene expression profling of human breast carcinoma-associated fibroblasts treated with paclitaxol or doxorubicin at therapeutically relevant doses | 0.85 | 0.85 | 0.85 | 0.81 | 0.86 | 0.77 | 0.86 | 0.90 | 0.82 |
| 30 | GSE14001 | PAX2: A Potential Biomarker for Low Malignant Potential Ovarian Tumors and Low-Grade Serous Ovarian Carcinomas | 0.89 | 0.87 | 0.88 | 0.82 | 0.80 | 0.69 | 0.87 | 0.84 | 0.83 |
| 31 | GSE17368 | Epiphyseal cartilage | 0.89 | 0.87 | 0.75 | 0.82 | 0.78 | 0.87 | 0.82 | 0.86 | 0.89 |
| 32 | GSE19495 | Global Gene Expression of Human Hepatoma Cells After Amino Acid Limitation | 0.60 | 0.83 | 0.87 | 0.88 | 0.81 | 0.89 | 0.86 | 0.74 | 0.82 |
| 33 | GSE14773 | Roles of EMT regulator in colon cancer | 0.88 | 0.89 | 0.74 | 0.77 | 0.79 | 0.81 | 0.81 | 0.85 | 0.90 |
| 34 | GSE19136 | Gene expression response to implanted drug (paclitaxel)-eluting or bare metal stents in denuded human LIMA arteries | 0.85 | 0.84 | 0.86 | 0.77 | 0.74 | 0.79 | 0.84 | 0.90 | 0.87 |
| 35 | GSE16356 | Lymphatic endothelial cells (LEC) treated with a MAF-targeted siRNA | 0.84 | 0.78 | 0.81 | 0.75 | 0.85 | 0.77 | 0.87 | 0.84 | 0.48 |
| 36 | GSE9835 | Gene Expression Changes in Response to Baculoviral Vector Transduction of Neuronal Cells In Vitro | 0.62 | 0.87 | 0.82 | 0.79 | 0.89 | 0.87 | 0.76 | 0.87 | 0.68 |
| 37 | GSE17044 | Expression data from androgen treated LNCaP cells | 0.89 | 0.80 | 0.88 | 0.69 | 0.85 | 0.73 | 0.77 | 0.89 | 0.62 |
| 38 | GSE9649 | Expression studies of HMEC exposed to lactic acidosis and hypoxia | 0.86 | 0.74 | 0.85 | 0.78 | 0.81 | 0.86 | 0.72 | 0.87 | 0.80 |
| 39 | GSE31641 | Expression data from treatment of human melanocytes with phenolic compounds | 0.85 | 0.79 | 0.83 | 0.70 | 0.84 | 0.75 | 0.83 | 0.86 | 0.90 |
| 40 | GSE13142 | HepG2/C3A cells cultured for 42 h in complete or leucine-devoid medium | 0.89 | 0.48 | 0.84 | 0.88 | 0.87 | 0.85 | 0.75 | 0.89 | 0.88 |
| 41 | GSE7345 | Germline NRAS mutation causes a novel human autoimmune lymphoproliferative syndrome | 0.86 | 0.66 | 0.73 | 0.83 | 0.86 | 0.83 | 0.75 | 0.88 | 0.87 |
| 42 | GSE34635 | Defining a No Observable Transcriptional Effect Level (NOTEL) for low dose N-OH-PhIP exposures in human BEAS-2B bronchioepithelial cells | 0.84 | 0.82 | 0.82 | 0.79 | 0.50 | 0.86 | 0.86 | 0.86 | 0.81 |
| 43 | GSE5838 | Expression data from transplanted intestine bifor signs of rejection, and when their was signs of rejection | 0.86 | 0.58 | 0.72 | 0.86 | 0.80 | 0.87 | 0.79 | 0.88 | 0.89 |
| 44 | GSE51130 | Using a rhabdomyosarcoma patient-derived xenograft to examine precision medicine approaches and model acquired resistance | 0.88 | 0.57 | 0.85 | 0.76 | 0.80 | 0.84 | 0.79 | 0.89 | 0.76 |
| 45 | GSE34512 | PBEF Knockdown in HMVEC-LBI | 0.87 | 0.88 | 0.84 | 0.84 | 0.84 | 0.80 | 0.39 | 0.89 | 0.46 |
| 46 | GSE42853 | Distinct gene expression profiles associated with the susceptibility of pathogen-specific CD4 T cells to HIV-1 infection | 0.86 | 0.65 | 0.83 | 0.81 | 0.86 | 0.58 | 0.87 | 0.89 | 0.89 |
| 47 | GSE26884 | Bisphenol A Induced the Expression of DNA Repair Genes in Human Breast Epithelial Cells | 0.80 | 0.84 | 0.80 | 0.62 | 0.83 | 0.88 | 0.67 | 0.76 | 0.76 |
| 48 | GSE53731 | Expression data from hepatitis E virus inoculated PLC/PRF/5 cells | 0.77 | 0.85 | 0.75 | 0.85 | 0.73 | 0.65 | 0.83 | 0.78 | 0.82 |
| 49 | GSE8588 | OH-PBDE-induced gene expression profiling in H295R adrenocortical carcinoma cells | 0.87 | 0.52 | 0.85 | 0.69 | 0.83 | 0.82 | 0.85 | 0.88 | 0.82 |
| 50 | GSE12875 | Impaired T-cell function in patients with novel ICOS | 0.86 | 0.52 | 0.87 | 0.83 | 0.82 | 0.77 | 0.75 | 0.88 | 0.83 |
| 51 | GSE45636 | eIF3a in Urinary Bladder Cancer ‚Äì in vivo and in vitro insights | 0.87 | 0.84 | 0.81 | 0.54 | 0.86 | 0.81 | 0.68 | 0.83 | 0.72 |
| 52 | GSE49085 | Identification of bone morphogenetic protein (BMP)-7 as a key instructive factor for human epidermal Langerhans cell differentiation and proliferation | 0.84 | 0.82 | 0.84 | 0.86 | 0.76 | 0.60 | 0.68 | 0.85 | 0.81 |
| 53 | GSE31472 | Host cell gene expression in Influenza A/duck/Malaysia/F118/08/2004 (H5N2) infected A549 cells at 2, 4, 6, 8, and 10 hours post infection | 0.80 | 0.84 | 0.84 | 0.56 | 0.86 | 0.80 | 0.69 | 0.88 | -0.37 |
| 54 | GSE9677 | Gene expression profile in HUVECs before and after Angiopoietin stimulation | 0.83 | 0.86 | 0.85 | 0.85 | 0.73 | 0.83 | 0.44 | 0.87 | 0.69 |
| 55 | GSE6400 | Cultured A549 lung cancer cells treated with actinomycin D and sapphyrin PCI-2050 | 0.83 | 0.81 | 0.85 | 0.78 | 0.58 | 0.79 | 0.71 | 0.77 | 0.53 |
| 56 | GSE16089 | Networking of differentially expressed genes in human Saos-2 osteosarcoma cells resistant to methotrexate | 0.85 | 0.73 | 0.36 | 0.85 | 0.86 | 0.86 | 0.85 | 0.89 | 0.89 |
| 57 | GSE15065 | C/EBPbeta-2 regulation of gene expression in MCF10A cells | 0.86 | 0.82 | 0.78 | 0.74 | 0.80 | 0.83 | 0.49 | 0.86 | 0.82 |
| 58 | GSE5110 | 48h Immobilization in human | 0.77 | 0.73 | 0.73 | 0.84 | 0.61 | 0.81 | 0.82 | 0.87 | 0.76 |
| 59 | GSE44540 | Gene expression in hTERT-RPE1 cells with overexpression of MFRP | 0.85 | 0.84 | 0.79 | 0.86 | 0.39 | 0.75 | 0.84 | 0.89 | 0.83 |
| 60 | GSE23764 | Expression data from actomyosin contractility regulated genes | 0.80 | 0.69 | 0.82 | 0.77 | 0.72 | 0.80 | 0.70 | 0.86 | 0.72 |
| 61 | GSE20037 | cdr2 siRNA knockdown during passage through mitosis: HeLa cells, Rat1 wild type and c-myc null cells | 0.80 | 0.46 | 0.82 | 0.79 | 0.79 | 0.82 | 0.80 | 0.70 | 0.47 |
| 62 | GSE40517 | Selective Requirement for Mediator MED23 in Ras-active Lung Cancer | 0.79 | 0.75 | 0.84 | 0.65 | 0.82 | 0.77 | 0.65 | 0.71 | 0.79 |
| 63 | GSE19510 | Transcriptional response of normal human lung WI-38 fibroblasts to benzo[a]pyrene diol epoxide: a dose-response study | 0.82 | 0.86 | 0.66 | 0.64 | 0.79 | 0.73 | 0.77 | 0.85 | 0.88 |
| 64 | GSE17785 | Endogenous expression of an oncogenic PI3K mutation leads to activated PI3K pathway signaling and an invasive phenotype | 0.83 | 0.82 | 0.81 | 0.51 | 0.78 | 0.81 | 0.65 | 0.86 | 0.80 |
| 65 | GSE29384 | Tetracycline-Inducible Cyr61 effect on LN229 glioma cells | 0.79 | 0.83 | 0.84 | 0.83 | 0.83 | 0.25 | 0.83 | 0.86 | 0.68 |
| 66 | GSE28339 | Gene expression data following Cyclin T2 and Cyclin T1 depletion by shRNA in HeLa cells | 0.83 | 0.81 | 0.53 | 0.54 | 0.83 | 0.83 | 0.76 | 0.83 | 0.65 |
| 67 | GSE2328 | Application of genome-wide expression analysis to human health & disease | 0.77 | 0.78 | 0.76 | 0.36 | 0.83 | 0.80 | 0.81 | 0.85 | 0.78 |
| 68 | GSE33606 | Gene expression changes in human hepatocytes exposed to VX (O-ethyl S-[2-(diisopropylamino)ethyl] methylphosphonothiolate) | 0.82 | 0.77 | 0.81 | 0.53 | 0.84 | 0.75 | 0.54 | 0.86 | 0.84 |
| 69 | GSE37648 | Gene signatures of normal hTERT immortalized ovarian epithelium and fallopian tube epithelium (paired cultures from 2 donor patients) | 0.84 | 0.77 | 0.71 | 0.74 | 0.75 | 0.77 | 0.50 | 0.79 | 0.82 |
| 70 | GSE45417 | Expression data from knockdown of ZXDC1/2 in PMA-treated U937 | 0.79 | 0.74 | 0.75 | 0.79 | 0.74 | 0.81 | 0.45 | 0.80 | 0.85 |
| 71 | GSE37474 | Dexamethasone induced gene expression changes in the human trabecular meshwork | 0.79 | 0.78 | 0.74 | 0.72 | 0.74 | 0.57 | 0.69 | 0.83 | 0.77 |
| 72 | GSE24224 | Analysis of genome-wide methylation and gene expression induced by decitabine treatment in HL60 leukemia cell line | 0.70 | 0.80 | 0.81 | 0.62 | 0.51 | 0.78 | 0.82 | 0.63 | 0.51 |
| 73 | GSE26599 | Gene expression profile in response to doxorubicin-rapamycin combined treatment of HER-2 overexpressing human mammary epithelial cell lines | 0.82 | 0.81 | 0.81 | 0.78 | 0.79 | 0.55 | 0.47 | 0.81 | 0.62 |
| 74 | GSE20125 | Transcriptome analysis of human Wharton‚Äôs jelly stem cells: meta-analysis | 0.75 | 0.73 | 0.70 | 0.80 | 0.77 | 0.48 | 0.72 | 0.84 | 0.81 |
| 75 | GSE47874 | The Heritage (HEalth, RIsk factors, exercise Training And GEnetics) family study | 0.75 | 0.64 | 0.77 | 0.74 | 0.77 | 0.63 | 0.66 | 0.78 | 0.79 |
| 76 | GSE6494 | Expression data from human liver cell line induced by PCB153 | 0.80 | 0.75 | 0.63 | 0.57 | 0.75 | 0.65 | 0.78 | 0.75 | 0.59 |
| 77 | GSE38517 | Expression data from fibroblasts derived from human normal oral mucosa, oral dysplasia and oral squamous cell carcinoma | 0.82 | 0.82 | 0.73 | 0.75 | 0.73 | 0.64 | 0.41 | 0.82 | 0.81 |
| 78 | GSE33243 | Human acute myelogenous leukemia-initiating cells treated with fenretinide | 0.78 | 0.46 | 0.66 | 0.73 | 0.68 | 0.76 | 0.81 | 0.80 | 0.73 |
| 79 | GSE22533 | Breast cancer cells resistant to hormone deprivation maintain an estrogen receptor alpha-dependent, E2F-directed transcriptional program | 0.81 | 0.83 | 0.78 | 0.82 | 0.81 | 0.41 | 0.40 | 0.85 | 0.85 |
| 80 | GSE30494 | Expression data in cancer cell lines using Affymetrix GeneChip | 0.63 | 0.79 | 0.80 | 0.79 | 0.36 | 0.69 | 0.76 | 0.79 | -0.37 |
| 81 | GSE4824 | Analysis of lung cancer cell lines | 0.77 | 0.80 | 0.69 | 0.45 | 0.80 | 0.60 | 0.70 | 0.78 | 0.81 |
| 82 | GSE15372 | Expression data from A2780 (cisplatin-sensitive) and Round5 A2780 (cisplatin-resistant) cell lines. | 0.53 | 0.78 | 0.82 | 0.76 | 0.79 | 0.48 | 0.64 | 0.81 | 0.47 |
| 83 | GSE16524 | Expression data from skin fibroblasts derived from Setleis Syndrome patients and normal controls | 0.81 | 0.68 | 0.70 | 0.73 | 0.72 | 0.66 | 0.50 | 0.84 | 0.72 |
| 84 | GSE13054 | Genes upregulated by HLX | 0.82 | 0.37 | 0.70 | 0.69 | 0.78 | 0.75 | 0.68 | 0.80 | 0.80 |
| 85 | GSE12274 | Mesenchymal Stromal Cells of Different Donor Age | 0.80 | 0.82 | 0.53 | 0.71 | 0.77 | 0.60 | 0.56 | 0.84 | 0.79 |
| 86 | GSE35170 | Expression data from U87-2M1 glioma cells transduced with baculoviral control decoy vector or baculoviral miR-10b decoy vector | 0.78 | 0.71 | 0.68 | 0.38 | 0.72 | 0.73 | 0.77 | 0.80 | 0.41 |
| 87 | GSE20540 | Gene expression profiles of myeloma cells interacting with bone marrow stromal cells in vitro | 0.72 | 0.68 | 0.73 | 0.75 | 0.76 | 0.34 | 0.76 | 0.36 | 0.37 |
| 88 | GSE4289 | Host transcriptome changes associated with episome loss and selection of keratinocytes containing integrated HPV16 | 0.77 | 0.80 | 0.80 | 0.62 | 0.57 | 0.78 | 0.39 | 0.71 | 0.79 |
| 89 | GSE11352 | Timecourse of estradiol (10nM) exposure in MCF7 breast cancer cells. | 0.73 | 0.66 | 0.77 | 0.69 | 0.61 | 0.58 | 0.67 | 0.77 | 0.56 |
| 90 | GSE39999 | Filarial nematode AsnRS interacts with interleukin 8 receptors in iDCs but causes different gene expression patterns compared to iDCs stimulated by interleukin 8. | 0.72 | 0.75 | 0.74 | 0.72 | 0.73 | 0.58 | 0.66 | 0.80 | 0.81 |
| 91 | GSE11208 | Chronic nicotine exposure (kuo-affy-human-232930) | 0.70 | 0.72 | 0.47 | 0.75 | 0.72 | 0.67 | 0.65 | 0.68 | 0.70 |
| 92 | GSE14986 | Antiestrogen-resistant subclones of MCF-7 human breast cancer cells are derived from a common clonal drug-resistant progenitor | 0.70 | 0.57 | 0.66 | 0.62 | 0.74 | 0.73 | 0.67 | 0.74 | 0.62 |
| 93 | GSE16538 | Genome-wide gene expression profile analysis in pulmonary sarcoidosis | 0.75 | 0.67 | 0.71 | 0.70 | 0.73 | 0.68 | 0.42 | 0.70 | 0.71 |
| 94 | GSE18182 | Expression profile of lung adenocarcinoma, A549 cells following targeted depletion of non metastatic 2 (NME2/NM23 H2) | 0.74 | 0.62 | 0.46 | 0.70 | 0.81 | 0.71 | 0.61 | 0.75 | -0.05 |
| 95 | GSE32892 | A genome-wide and dose-dependent inhibition map of androgen receptor binding by small molecules reveals its regulatory program upon antagonism | 0.74 | 0.78 | 0.76 | 0.81 | 0.76 | 0.68 | 0.11 | 0.82 | 0.78 |
| 96 | GSE40220 | INTESTINAL FILTER FOR USE IN OESOPHAGEAL CANCER RESEARCH | 0.73 | 0.80 | 0.74 | 0.75 | 0.67 | 0.80 | 0.14 | 0.74 | 0.75 |
| 97 | GSE33146 | Expression data from DKAT breast cancer cell line pre- and post-EMT | 0.78 | 0.72 | 0.74 | 0.74 | 0.52 | 0.69 | 0.45 | 0.63 | -0.09 |
| 98 | GSE17624 | Expression data from human Ishikawa cells treated with Bisphenol A | 0.73 | 0.78 | 0.60 | 0.61 | 0.75 | 0.72 | 0.43 | 0.80 | 0.39 |
| 99 | GSE17090 | Expression data from human adipose stem cells expanded in allogeneic human serum and fetal bovine serum | 0.77 | 0.72 | 0.75 | 0.72 | 0.50 | 0.59 | 0.52 | 0.78 | 0.80 |
| 100 | GSE16054 | Transient expression of misfolded surfactant protein C | 0.56 | 0.56 | 0.79 | 0.76 | 0.75 | 0.70 | 0.41 | 0.76 | 0.69 |
| 101 | GSE16715 | Expression profiling in Williams-Beuren Syndrome patient fibroblast cell lines | 0.76 | 0.61 | 0.62 | 0.52 | 0.73 | 0.58 | 0.70 | 0.78 | 0.73 |
| 102 | GSE9593 | Cellular Aging of Mesenchymal Stem Cells | 0.75 | 0.77 | 0.40 | 0.63 | 0.78 | 0.74 | 0.43 | 0.79 | 0.73 |
| 103 | GSE7846 | Differentially expressed genes in HEECs of eutopic endometrium of patients with endometriosis compared with control | 0.72 | 0.69 | 0.77 | 0.62 | 0.64 | 0.35 | 0.67 | 0.80 | 0.70 |
| 104 | GSE13671 | Expression data from mammary epithelial cells from BRCA1 mutation carriers and non BRCA1 mutation carriers | 0.75 | 0.70 | 0.54 | 0.61 | 0.51 | 0.72 | 0.65 | 0.67 | 0.75 |
| 105 | GSE8045 | Gene Expression Profiling in A549 Lung Cancer Cell Line Following siRNA Mediated Knock-down of ALDH1A1 and ALDH3A1 | 0.78 | 0.75 | 0.70 | 0.72 | 0.67 | 0.16 | 0.70 | 0.80 | 0.81 |
| 106 | GSE11550 | Hs 294T Cells Treated with Elesclomol Alone or in Combination with Paclitaxel Compared to DMSO Treated | 0.70 | 0.48 | 0.76 | 0.49 | 0.74 | 0.65 | 0.66 | 0.81 | 0.54 |
| 107 | GSE6869 | Expression data from human liver cell line induced by PCB77 | 0.72 | 0.76 | 0.51 | 0.66 | 0.70 | 0.66 | 0.45 | 0.75 | 0.64 |
| 108 | GSE51549 | All trans-retinoic acid (ATRA) re-differentiate early transformed breast epithelial cells to normal. | 0.79 | 0.69 | 0.73 | 0.40 | 0.46 | 0.70 | 0.67 | 0.80 | 0.66 |
| 109 | GSE32939 | CD4 on human monocytes | 0.80 | 0.42 | 0.59 | 0.69 | 0.41 | 0.78 | 0.77 | 0.83 | 0.73 |
| 110 | GSE16547 | KSHV Manipulates Notch Signaling by Upregulating Dll4 and JAG1 to Alter Cell Cycle Gene Expression in LECs | 0.75 | 0.70 | 0.74 | 0.71 | 0.73 | 0.75 | 0.06 | 0.77 | 0.75 |
| 111 | GSE14807 | Investigation of over-expressing Annexin receptor cell line with and without agonists | 0.57 | 0.75 | 0.69 | 0.75 | 0.60 | 0.66 | 0.76 | 0.59 | 0.37 |
| 112 | GSE18161 | Washing scaling of microarray expression | 0.63 | 0.74 | 0.66 | 0.42 | 0.71 | 0.53 | 0.69 | 0.72 | 0.71 |
| 113 | GSE9361 | Functional interaction between a PIP2 novel polyA polymerase and type 1 PIPKIalpha | 0.71 | 0.73 | 0.62 | 0.73 | 0.08 | 0.75 | 0.75 | 0.78 | 0.71 |
| 114 | GSE33455 | Expression data from docetaxel-resistant prostate cancer cell lines | 0.52 | 0.77 | 0.68 | 0.76 | 0.78 | 0.66 | 0.18 | 0.70 | -0.16 |
| 115 | GSE32984 | Gene expression profiling of Human Umbilical Vein Endothelial Cells (HUVEC) after treatment with Erg or control antisense (GeneBloc) | 0.66 | 0.72 | 0.63 | 0.47 | 0.75 | 0.70 | 0.42 | 0.75 | 0.65 |
| 116 | GSE30038 | Programming human pluripotent stem cells into adipocytes [Affymetrix] | 0.68 | 0.75 | 0.74 | 0.39 | 0.75 | 0.35 | 0.74 | 0.69 | 0.62 |
| 117 | GSE35957 | Effects of Cellular Senescence on Human Mesenchymal Stem Cells | 0.63 | 0.62 | 0.64 | 0.73 | 0.70 | 0.50 | 0.49 | 0.77 | 0.76 |
| 118 | GSE10934 | Human sclera | 0.71 | 0.74 | 0.69 | 0.75 | 0.73 | 0.02 | 0.68 | 0.79 | 0.76 |
| 119 | GSE18892 | Silencing of AEBP1 in U87MG glial cells and Chip-chIP with AEBP1 antibody | 0.78 | 0.71 | 0.70 | 0.58 | 0.45 | 0.65 | 0.57 | 0.78 | 0.75 |
| 120 | GSE47873 | Gene expression profiles MCF-10A cells overexpressing MBD2 | 0.79 | 0.76 | 0.39 | 0.74 | 0.69 | 0.75 | 0.17 | 0.78 | 0.80 |
| 121 | GSE31469 | Host cell gene expression in Influenza A/WSN/33 (H1N1) infected A549 cells at 2, 4 and 6 hours post infection | 0.71 | 0.32 | 0.64 | 0.52 | 0.67 | 0.72 | 0.71 | 0.77 | 0.25 |
| 122 | GSE28448 | Antagonistic regulation of EMT by TIF1Œ≥ and Smad4 in mammary epithelial cells | 0.56 | 0.74 | 0.73 | 0.59 | 0.65 | 0.73 | 0.30 | 0.66 | 0.71 |
| 123 | GSE18866 | Expression data from doxycylin-inducible miR-15a/16-1 and empty vector (EV) expression in a 13q14-\- cell line | 0.68 | 0.71 | 0.44 | 0.72 | 0.30 | 0.74 | 0.70 | 0.76 | 0.28 |
| 124 | GSE28829 | Gene Expression in early and advanced atherosclerotic plaque from human carotid | 0.71 | 0.72 | 0.68 | 0.66 | 0.45 | 0.44 | 0.59 | 0.76 | 0.65 |
| 125 | GSE9768 | Identification of genes modulated by acid and bile in a Barrett's oesophagus cell line | 0.75 | 0.69 | 0.11 | 0.74 | 0.75 | 0.71 | 0.52 | 0.76 | 0.47 |
| 126 | GSE7700 | Genes regulated by YAP in normal breast cell line and breast cancer cell lines | 0.76 | 0.74 | 0.74 | 0.76 | 0.73 | -0.24 | 0.76 | 0.79 | 0.80 |
| 127 | GSE16962 | Effect of mir-210 overexpression or down-modulation on human umbilical vein cells | 0.72 | 0.70 | 0.75 | 0.72 | 0.31 | 0.75 | 0.31 | 0.73 | 0.72 |
| 128 | GSE21570 | Frizzled 4 regulates stemness and invasiveness of migrating glioma cells established by serial in vivo intracranial transplantation | 0.66 | 0.77 | 0.75 | 0.63 | 0.67 | 0.72 | 0.36 | 0.65 | 0.71 |
| 129 | GSE25746 | Integrated, genome-wide screening for hypomethylated oncogenes in salivary gland adenoid cystic carcinoma | 0.72 | 0.73 | 0.12 | 0.69 | 0.63 | 0.57 | 0.76 | 0.75 | 0.76 |
| 130 | GSE27200 | Expression data from Sotos syndrome patients and controls | 0.73 | 0.66 | 0.54 | 0.58 | 0.63 | 0.53 | 0.54 | 0.74 | 0.78 |
| 131 | GSE61352 | Expression data from normal urothelial cells with exogenous expression of mutant FGFR3 | 0.32 | 0.75 | 0.78 | 0.70 | 0.63 | 0.78 | 0.61 | 0.48 | 0.72 |
| 132 | GSE7824 | Zonal Heterogeneity for Gene Expression in Human Pancreatic Carcinoma Growing in the Pancreas of Nude Mice | 0.69 | 0.68 | 0.62 | 0.62 | 0.54 | 0.61 | 0.45 | 0.68 | 0.62 |
| 133 | GSE17466 | Gene expression in iPrEC cell line transfected with wild-type USP2A, mutant USP2A, or empty vector. | 0.73 | 0.62 | 0.39 | 0.68 | 0.67 | 0.69 | 0.43 | 0.76 | 0.67 |
| 134 | GSE29265 | Sporadic vs. Post-Chernobyl Papillary vs. Anaplastic Thyroid Cancers | 0.65 | 0.66 | 0.64 | 0.57 | 0.64 | 0.44 | 0.59 | 0.48 | 0.74 |
| 135 | GSE42762 | FOXO3a Is A Major Target Of Inactivation By PI3K/AKT Signaling In Aggressive Neuroblastoma | 0.71 | 0.68 | 0.67 | 0.75 | 0.72 | 0.57 | 0.09 | 0.79 | 0.53 |
| 136 | GSE30336 | Expression analysis of 52 glioma clinical samples (36 CIMP+ and 16 CIMP-) and 6 cell line samples | 0.68 | 0.56 | 0.79 | 0.76 | 0.63 | 0.40 | 0.62 | 0.66 | 0.42 |
| 137 | GSE33168 | The amniotic fluid transcriptome: a source of novel information about human fetal development | 0.48 | 0.66 | 0.72 | 0.53 | 0.65 | 0.47 | 0.67 | 0.71 | 0.18 |
| 138 | GSE6460 | Human mesenchymal stem cells | 0.67 | 0.67 | 0.74 | 0.65 | 0.73 | 0.59 | 0.12 | 0.68 | 0.49 |
| 139 | GSE57441 | Genes expression of cervical squamous cell carcinoma, CaSki cells, treated with or without recombinant TGF-b1 (2 ng/mL) | 0.66 | 0.73 | 0.70 | -0.02 | 0.71 | 0.67 | 0.70 | 0.23 | -0.28 |
| 140 | GSE40611 | Gene expression data of parotid tissue from Primary Sjogren‚Äôs Syndrome and controls | 0.67 | 0.55 | 0.56 | 0.58 | 0.61 | 0.70 | 0.47 | 0.74 | 0.73 |
| 141 | GSE6679 | Staufen1 regulates a variety of mammalian transcripts | 0.68 | 0.76 | 0.60 | 0.73 | 0.30 | 0.77 | 0.53 | 0.38 | -0.11 |
| 142 | GSE36970 | KDM4B- and KDM6B-regulated genes in human mesenchymal stem cell osteogenic differentiation | 0.71 | 0.67 | 0.74 | 0.28 | 0.49 | 0.53 | 0.67 | 0.79 | 0.79 |
| 143 | GSE30391 | Expression data from human Wharton's jelly stem cells | 0.65 | 0.69 | 0.64 | 0.47 | 0.70 | 0.43 | 0.48 | 0.73 | 0.66 |
| 144 | GSE7807 | Leukotriene D4 induces gene expression in human monocytes through cysteinyl leukotriene type I receptor. | 0.69 | 0.64 | 0.74 | 0.43 | 0.71 | 0.34 | 0.59 | 0.65 | 0.57 |
| 145 | GSE35382 | Comparison of gene expression profiles of HT29 cells treated with Instant Caffeinated Coffee or Caffeic Acid versus control. | 0.62 | 0.54 | 0.52 | 0.73 | 0.65 | 0.68 | 0.46 | 0.65 | 0.55 |
| 146 | GSE48616 | Expression data from hBMSCs cultured on PLLA nanofibers | 0.73 | 0.74 | 0.51 | 0.73 | 0.65 | -0.10 | 0.72 | 0.78 | 0.77 |
| 147 | GSE8597 | Gene expression analysis of hormone treated MCF7 breast cancer cells in the presence or absence of cycloheximide | 0.72 | 0.70 | -0.29 | 0.73 | 0.72 | 0.72 | 0.66 | 0.76 | 0.76 |
| 148 | GSE47920 | Expression data from T lymphocytes derived from T-iPS and peripheral blood | 0.70 | 0.66 | 0.25 | 0.70 | 0.74 | 0.33 | 0.58 | 0.73 | 0.67 |
| 149 | GSE9212 | Overexpression of lung developmental transcription factors TTF-1, NKX2-8 and PAX9 | 0.70 | 0.69 | 0.18 | 0.53 | 0.61 | 0.63 | 0.58 | 0.69 | 0.48 |
| 150 | GSE19123 | Lactic acidosis triggers starvation response with distinct metabolic profiles | 0.66 | 0.63 | 0.53 | 0.63 | 0.62 | 0.36 | 0.48 | 0.67 | 0.68 |
| 151 | GSE25148 | Changes in gene expression in HEK-TLR2 cells in response to Helicobacter pylori lipopolysaccharide | 0.68 | 0.66 | 0.59 | 0.59 | 0.55 | 0.52 | 0.44 | 0.70 | 0.61 |
| 152 | GSE13330 | Senescent Stromal-Derived Osteopontin Promotes Preneoplastic Cell Growth | 0.59 | 0.61 | 0.70 | 0.57 | 0.04 | 0.70 | 0.68 | 0.75 | 0.75 |
| 153 | GSE57634 | Molecular effects of EtOH and Nicotine on normal human oral keratinocytes | 0.61 | 0.28 | 0.68 | 0.60 | 0.58 | 0.53 | 0.60 | 0.66 | 0.23 |
| 154 | GSE26704 | p38alfa and ATF2 act differentially depending on DUSP1 expression in NCSCL in response to cisplatin | 0.70 | 0.66 | 0.52 | 0.51 | 0.54 | 0.61 | 0.45 | 0.73 | 0.68 |
| 155 | GSE37136 | Gene expression profiling of enforced HOXA1 expression in melanoma cell line | 0.73 | 0.72 | 0.73 | 0.74 | 0.74 | 0.74 | -0.55 | 0.77 | 0.69 |
| 156 | GSE27316 | Effects of long dsRNA expression in HeLa and HEK293 cells | 0.73 | 0.72 | 0.32 | 0.71 | 0.71 | 0.73 | -0.09 | 0.77 | 0.69 |
| 157 | GSE60771 | Testing gene expression changes in VCaP upon depletion of the mutated ETS transcription factor ERG | 0.66 | 0.50 | 0.62 | 0.67 | 0.71 | 0.67 | -0.02 | 0.71 | 0.67 |
| 158 | GSE11418 | Passage dependent gene expression in normal human dermal fibroblasts | 0.66 | 0.61 | 0.28 | 0.54 | 0.63 | 0.43 | 0.68 | 0.75 | 0.67 |
| 159 | GSE31534 | Gene expression profile in A375 melanoma cells after 45 functionally important molecules were knocked down using siRNA | 0.67 | 0.67 | 0.58 | 0.66 | 0.67 | 0.56 | -0.04 | 0.71 | 0.67 |
| 160 | GSE7268 | Cryptosporidium infection of human intestinal tissues causes increased expression of Osteoprotegerin | 0.66 | 0.61 | 0.49 | 0.61 | 0.45 | 0.60 | 0.47 | 0.59 | 0.55 |
| 161 | GSE14334 | Transcriptomic analysis of human lung development | 0.60 | 0.65 | 0.63 | 0.61 | 0.65 | -0.09 | 0.65 | 0.68 | 0.71 |
| 162 | GSE13491 | Therapeutic efficacy of human umbilical cord blood-derived mesenchymal stem cells in myocardial repair after infarction | 0.71 | 0.63 | 0.66 | 0.66 | 0.69 | 0.63 | -0.29 | 0.75 | 0.72 |
| 163 | GSE42924 | Expression data from immature dendritic cells (iDC) expressing HIV-1 Tat alleles and mutants | 0.58 | 0.67 | 0.49 | 0.28 | 0.61 | 0.50 | 0.55 | 0.71 | 0.61 |
| 164 | GSE12198 | Primary NKcells vs. NKAES-derived NK cells vs. NKcells stimulated by low/high dose IL2 after 7days of culture | 0.67 | 0.29 | 0.33 | 0.68 | 0.67 | 0.50 | 0.54 | 0.74 | 0.73 |
| 165 | GSE20538 | Gene expression profiles of fibroblasts from MCT8 patients | 0.69 | 0.63 | 0.36 | 0.68 | 0.62 | 0.43 | 0.25 | 0.73 | 0.71 |
| 166 | GSE47746 | Gene expression data of fibroblasts transduced with LacZ or p63+KLF4. | 0.71 | 0.71 | 0.53 | 0.68 | 0.64 | -0.06 | 0.45 | 0.76 | 0.64 |
| 167 | GSE23586 | Altered gene expressions of leukocyte transendothelial migration and cell communication pathways in periodontitis-affected gingival tissues | 0.67 | 0.31 | 0.69 | 0.70 | 0.52 | 0.72 | 0.45 | 0.79 | 0.64 |
| 168 | GSE11407 | SCFA-hexosamine scaffold | 0.50 | 0.61 | 0.66 | 0.44 | 0.50 | 0.46 | 0.48 | 0.72 | 0.65 |
| 169 | GSE4984 | Monocyte Derived Dendritic Cell Maturation | 0.59 | 0.56 | 0.17 | 0.65 | 0.64 | 0.49 | 0.59 | 0.52 | 0.38 |
| 170 | GSE45426 | Transcriptional events in human skeletal muscle at the outset of concentric resistance exercise training | 0.66 | 0.57 | 0.65 | 0.36 | 0.62 | 0.19 | 0.59 | 0.68 | 0.73 |
| 171 | GSE20433 | Effects of T350 phospho- on gene repression activity of EZH2 | 0.70 | 0.59 | 0.65 | 0.66 | -0.23 | 0.68 | 0.58 | 0.76 | 0.58 |
| 172 | GSE39762 | Genome Wide Profiling of p53 Response to Differentiation or DNA Damage of Human Embryonic Stem Cells | 0.71 | 0.66 | 0.59 | -0.22 | 0.53 | 0.65 | 0.70 | 0.73 | 0.65 |
| 173 | GSE55609 | Human meningioma culture | 0.69 | 0.55 | 0.63 | 0.56 | 0.39 | 0.56 | 0.24 | 0.70 | 0.70 |
| 174 | GSE7888 | Expression data from human mesenchymal stem cells (six batches) | 0.63 | 0.60 | 0.42 | 0.50 | 0.49 | 0.52 | 0.46 | 0.67 | 0.64 |
| 175 | GSE34112 | Effect of NO-sulindac treatment on hypoxic prostate cancer cells | 0.71 | 0.62 | 0.40 | -0.04 | 0.57 | 0.70 | 0.61 | 0.71 | 0.63 |
| 176 | GSE11869 | The genomic response of a human uterine endometrial adenocarcinoma cell line to 17alpha-ethynyl estradiol. | 0.51 | 0.60 | 0.59 | 0.42 | 0.34 | 0.60 | 0.46 | 0.45 | 0.12 |
| 177 | GSE7224 | Gene expression in tonsil and oral epithelia | 0.65 | 0.36 | 0.69 | 0.64 | -0.05 | 0.68 | 0.56 | 0.67 | 0.71 |
| 178 | GSE53059 | Human Subperitoneal Fibroblasts and Cancer Cell Interaction Creates Microenvironment Enhancing Tumor Progression and Metastasis | 0.66 | 0.65 | 0.68 | 0.65 | 0.44 | -0.20 | 0.62 | 0.72 | 0.70 |
| 179 | GSE27041 | OXPHOS complex I deficiency leads to transcriptional changes of the Nrf2-Keap1 pathway and selenoproteins. | 0.58 | 0.59 | 0.29 | 0.63 | 0.55 | 0.34 | 0.49 | 0.64 | 0.51 |
| 180 | GSE4587 | Whole-genome expression profiling of melanoma progression. | 0.69 | 0.67 | 0.39 | 0.66 | 0.65 | 0.51 | -0.09 | 0.72 | 0.69 |
| 181 | GSE11622 | Molecular Analysis of the Vaginal Response to Estrogens in the Ovariectomized Rat and Postmenopausal Woman | 0.68 | 0.61 | 0.45 | 0.34 | 0.22 | 0.66 | 0.51 | 0.73 | 0.66 |
| 182 | GSE42253 | Gene expression data from T cells and NK cells with and without treatment with Hsp90 inhibitor (Geldanamycin) | 0.71 | 0.70 | 0.61 | -0.02 | 0.63 | 0.68 | 0.19 | 0.75 | 0.72 |
| 183 | GSE4237 | Hussaini-2R01NS035122-06A1 | 0.60 | 0.57 | 0.61 | 0.63 | 0.60 | 0.54 | -0.11 | 0.72 | 0.43 |
| 184 | GSE24235 | Skeletal muscle gene expression in response to resistance exercise: sex specific regulation | 0.56 | 0.46 | 0.55 | 0.41 | 0.65 | 0.36 | 0.42 | 0.56 | 0.53 |
| 185 | GSE30806 | Expression profiling in VCP-associated myopathy | 0.59 | 0.52 | 0.50 | 0.25 | 0.48 | 0.47 | 0.64 | 0.55 | 0.61 |
| 186 | GSE15543 | Meta analysis of gene expression in human islets after in vitro expansion. | 0.68 | 0.50 | 0.57 | 0.55 | 0.52 | 0.16 | 0.39 | 0.70 | 0.64 |
| 187 | GSE33643 | Comparison of gene expression alterations induced by distinct PI3K inhibitors | 0.46 | 0.54 | 0.66 | 0.62 | 0.33 | 0.33 | 0.41 | 0.64 | 0.61 |
| 188 | GSE46914 | IDENTIFICATION OF BIOMARKERS OF RESPONSE TO IFNG DURING ENDOTOXIN TOLERANCE: APPLICATION TO SEPTIC SHOCK | 0.53 | 0.57 | 0.64 | 0.48 | 0.42 | 0.09 | 0.62 | 0.52 | 0.54 |
| 189 | GSE36895 | Molecular Genetic Classification of clear-cell Renal Cell Carcinoma (ccRCC) based on the Gene Expression Profiling of Tumors and Tumorgrafts deficient for BAP1 or PBRM1 | 0.65 | 0.55 | 0.41 | 0.46 | 0.50 | 0.47 | 0.41 | 0.58 | 0.59 |
| 190 | GSE44596 | The effect of Sparstolonin B (SsnB) on gene expression in HCAECs | 0.67 | 0.65 | 0.63 | 0.65 | 0.65 | 0.68 | -0.61 | 0.71 | 0.68 |
| 191 | GSE57463 | SOX9 overexpression in melanoma | 0.66 | 0.68 | -0.54 | 0.68 | 0.59 | 0.62 | 0.61 | 0.72 | 0.67 |
| 192 | GSE19315 | Global transcriptional response of macrophage-like THP-1 cells | 0.65 | 0.66 | -0.36 | 0.69 | 0.57 | 0.63 | 0.51 | 0.69 | 0.73 |
| 193 | GSE16354 | Infection of Lymphatic and Blood Vessel Endothelial Cells (LEC and BEC) with KSHV | 0.64 | 0.47 | 0.33 | 0.56 | 0.57 | 0.36 | 0.38 | 0.64 | 0.69 |
| 194 | GSE13487 | Antitumor efficacy of RAF inhibitor GDC-0879 involving BRAFV600E mutational status and ERK/MAPK pathway suppression | 0.61 | 0.42 | 0.59 | 0.45 | 0.58 | 0.55 | 0.09 | 0.58 | 0.18 |
| 195 | GSE23994 | A molecular signature of normal breast epithelial and stromal cells from Li-Fraumeni syndrome mutation carriers | 0.66 | 0.65 | 0.62 | 0.57 | 0.65 | -0.50 | 0.63 | 0.73 | 0.70 |
| 196 | GSE36807 | Genome-wide analysis of Crohn's disease and ulcerative colitis biopsy samples. | 0.36 | 0.52 | 0.43 | 0.56 | 0.49 | 0.64 | 0.42 | 0.23 | 0.59 |
| 197 | GSE23849 | The ERAD inhibitor Eeyarestatin I is a bifunctional compound with an ER localizing domain and a p97/VCP inhibitory group | 0.68 | 0.68 | 0.39 | -0.08 | 0.50 | 0.49 | 0.65 | 0.72 | 0.63 |
| 198 | GSE33301 | Expression data from control and COUP-TFII siRNA treated HUVEC cells | 0.74 | 0.51 | 0.73 | 0.52 | 0.62 | 0.69 | 0.12 | 0.77 | 0.69 |
| 199 | GSE14897 | Highly Efficient generation of Human Hepatic Cells from Induced Pluripotent Stem Cells. | 0.63 | -0.08 | 0.63 | 0.59 | 0.58 | 0.62 | 0.27 | 0.70 | 0.66 |
| 200 | GSE43489 | Expression profile from PCE-Epi and derived cell lines | 0.82 | 0.67 | 0.79 | 0.81 | 0.80 | 0.60 | 0.49 | 0.75 | 0.79 |
| 201 | GSE46268 | Gene expression profile of human monocytes stimulated with all-trans retinoic acid (ATRA) or 1,25a-dihydroxyvitamin D3 (1,25D3) | 0.61 | 0.51 | -0.21 | 0.60 | 0.59 | 0.63 | 0.47 | 0.66 | 0.57 |
| 202 | GSE13376 | Exposure of Barrett's associated adenocarcinoma cell lines SKGT4 to deoxycholic acid (DCA) | 0.64 | 0.57 | -0.49 | 0.65 | 0.63 | 0.62 | 0.58 | 0.61 | -0.15 |
| 203 | GSE27850 | HAV acute infection in chimpanzees | 0.60 | 0.35 | 0.52 | 0.27 | 0.51 | 0.43 | 0.52 | 0.60 | 0.60 |
| 204 | GSE59412 | A Systems Biology Approach identified different regulatory networks targeted by KSHV miR-K12-11 in B cells and endothelial cells | 0.64 | 0.56 | 0.57 | 0.66 | 0.64 | -0.45 | 0.57 | 0.67 | 0.64 |
| 205 | GSE3397 | RSV gene expression | 0.65 | 0.63 | 0.39 | 0.14 | 0.16 | 0.65 | 0.57 | 0.70 | 0.22 |
| 206 | GSE52674 | expression data of miR-21 knockdown in MCF-7 | 0.51 | 0.66 | 0.65 | 0.65 | 0.58 | 0.63 | -0.51 | 0.59 | 0.64 |
| 207 | GSE49967 | Variability in functional p53 reactivation by Prima-1Met/APR-246 in Ewing sarcoma | 0.64 | 0.58 | 0.67 | 0.44 | 0.72 | 0.48 | 0.49 | 0.58 | 0.69 |
| 208 | GSE55859 | Gene expression profile of TRAIL-sensitive and -resistant H460 cells | 0.67 | 0.66 | 0.66 | 0.67 | 0.67 | 0.63 | -0.84 | 0.71 | -0.18 |
| 209 | GSE13637 | Influenza virus infected HUVEC | 0.39 | 0.59 | 0.40 | 0.15 | 0.58 | 0.55 | 0.46 | 0.60 | 0.37 |
| 210 | GSE31980 | Transcriptome profile in the human synovial MSC-aggregates | 0.66 | 0.64 | 0.60 | 0.63 | 0.66 | -0.71 | 0.62 | 0.70 | 0.71 |
| 211 | GSE45512 | Human Alopecia Areata Skin Profiling | 0.61 | 0.57 | 0.65 | 0.62 | -0.45 | 0.55 | 0.56 | 0.66 | 0.70 |
| 212 | GSE20141 | Expression analysis of laser-dissected SNpc neurons in Parkinson's disease | 0.61 | -0.22 | 0.58 | 0.60 | 0.54 | 0.61 | 0.38 | 0.67 | 0.61 |
| 213 | GSE17032 | Expression data from human fibroblasts | 0.56 | 0.58 | 0.37 | 0.53 | 0.51 | 0.22 | 0.31 | 0.64 | 0.47 |
| 214 | GSE41364 | Expression data for HT29 cells treated with 5-aza-deoxy-cytidine [Affymetrix] | 0.57 | 0.53 | 0.63 | 0.53 | -0.09 | 0.66 | 0.49 | 0.46 | 0.28 |
| 215 | GSE16870 | HeLa cells treated with V-ATPase inhibitors or with desoxyferramine compared to HeLa in DMSO or medium with low LDL | 0.51 | 0.64 | 0.59 | 0.44 | 0.58 | 0.61 | -0.32 | 0.62 | 0.58 |
| 216 | GSE48311 | Time-dependent changes in gene expression after endotoxinic challenge followed by LR12-scrambled or LR12 treatment. | 0.59 | 0.49 | 0.17 | 0.48 | 0.38 | 0.37 | 0.55 | 0.62 | 0.35 |
| 217 | GSE11142 | Nicotine effect on CEM model T cell line (kuo-affy-human-232861) | 0.61 | 0.38 | -0.07 | 0.59 | 0.56 | 0.32 | 0.63 | 0.60 | 0.62 |
| 218 | GSE49953 | Expression data from two breast cancer cell lines | 0.66 | 0.63 | 0.66 | 0.63 | 0.66 | 0.65 | -0.89 | 0.70 | 0.68 |
| 219 | GSE33630 | Normal thyrocytes vs papillary vs anaplastic thyroid carcinomas | 0.63 | 0.58 | 0.59 | 0.55 | 0.58 | 0.26 | 0.61 | 0.44 | 0.75 |
| 220 | GSE14987 | Expression data from ERBB2 over-expression and EGF stimulation in MCF10A cells | 0.64 | 0.66 | 0.64 | 0.63 | 0.60 | 0.61 | -0.83 | 0.68 | 0.67 |
| 221 | GSE5081 | Expression data from Helicobacter positive and negative human gastritis samples | 0.56 | 0.28 | 0.49 | 0.52 | 0.34 | 0.48 | 0.28 | 0.64 | 0.55 |
| 222 | GSE36765 | Gene expression profiling of CD4+ T cells infiltrating human breast cancer (Discovery Set) | 0.59 | 0.54 | 0.53 | 0.39 | 0.11 | 0.38 | 0.41 | 0.62 | 0.64 |
| 223 | GSE49628 | Large-scale hypomethylated blocks associated with Epstein-Barr virus-induced B-cell immortalization [Expression Array] | 0.66 | 0.50 | 0.55 | 0.61 | 0.47 | 0.61 | -0.38 | 0.70 | 0.65 |
| 224 | GSE12079 | Molecular profiling of CD3- CD4+ T-cells from patients with the lymphocytic variant of hypereosinophilic syndrome | 0.55 | -0.16 | 0.53 | 0.43 | 0.60 | 0.49 | 0.47 | 0.65 | 0.63 |
| 225 | GSE12355 | Detection of Notch1-IC, Notch2-IC and EBNA2 target genes in human B cells | 0.51 | 0.55 | 0.26 | 0.61 | 0.57 | 0.24 | 0.15 | 0.60 | 0.58 |
| 226 | GSE50208 | Molecular-guided therapy predictions reveal drug resistance phenotypes and treatment alternatives in malignant peripheral nerve sheath tumors | 0.63 | 0.64 | 0.60 | 0.62 | 0.61 | -0.38 | 0.17 | 0.69 | 0.60 |
| 227 | GSE4406 | Gene expression profiling of CD4+ T-cells and GM6990 lymphoblastoid cell lines | 0.63 | 0.62 | 0.64 | 0.63 | 0.62 | 0.52 | -0.79 | 0.68 | 0.68 |
| 228 | GSE33325 | Gene expression changes in human cardiomyocytes exposed to VX (O-ethyl S-[2-(diisopropylamino)ethyl] methylphosphonothiolate) | 0.60 | 0.55 | 0.60 | 0.51 | 0.32 | 0.59 | -0.32 | 0.64 | 0.62 |
| 229 | GSE17549 | Loss-of-function mutations in REP-1 affect intracellular vesicle transport in fibroblasts and monocytes of CHM patients | 0.53 | 0.58 | 0.60 | 0.61 | 0.43 | -0.31 | 0.42 | 0.57 | 0.57 |
| 230 | GSE19963 | Expression data from hyperplastic polyps and normal colonic mucosa from patients with familial and sporadic HPPS | 0.59 | 0.51 | 0.44 | 0.60 | 0.38 | 0.61 | -0.29 | 0.64 | 0.46 |
| 231 | GSE18934 | Gene expression in fetal mesenchymal stem cells for identification of epitopes suitable for non-invasive isolation | 0.63 | 0.62 | 0.61 | 0.63 | 0.62 | -0.92 | 0.64 | 0.68 | 0.64 |
| 232 | GSE21483 | Regulation of HB-EGF by miR-212 and acquired cetuximab-resistance in head and neck cancer | 0.58 | 0.61 | 0.62 | 0.63 | 0.62 | 0.64 | -0.91 | 0.44 | 0.64 |
| 233 | GSE16464 | Chondrogenic differentiation potential of OA chondrocytes and their use in autologous chondrocyte transplantation | 0.52 | 0.54 | 0.27 | 0.57 | 0.51 | -0.03 | 0.50 | 0.63 | 0.56 |
| 234 | GSE36767 | Gene changes of CD4+ T cells infiltrating human breast cancer in the absence of tumor environment (Confirmation Set 24h) | 0.64 | 0.63 | 0.57 | 0.58 | 0.42 | -0.65 | 0.60 | 0.67 | 0.69 |
| 235 | GSE18931 | The biological and molecular heterogeneity of breast cancers correlate with their cancer stem cell content | 0.60 | 0.58 | 0.58 | 0.45 | -0.53 | 0.43 | 0.62 | 0.67 | 0.64 |
| 236 | GSE37603 | Identification of WISP1 as an important survival factor in human mesenchymal stem cells | 0.62 | 0.58 | 0.57 | -0.46 | 0.60 | 0.47 | 0.49 | 0.48 | 0.62 |
| 237 | GSE15192 | Differences between CD44+/CD24- and CD44-/CD24+ subpopulation of immortalized human mammary epithelial cells | 0.59 | 0.62 | 0.62 | 0.61 | 0.57 | -0.93 | 0.61 | 0.61 | 0.01 |
| 238 | GSE40241 | Expression data from Versican (VCAN) protein treated ovarian cancer cell line OVCA433 | 0.59 | 0.55 | 0.55 | 0.61 | 0.58 | -0.73 | 0.61 | 0.69 | 0.58 |
| 239 | GSE18912 | Expression profiling of breast cancer cell lines MCF-7 and MCF-7R4 | 0.56 | -0.49 | 0.61 | 0.46 | 0.50 | 0.56 | 0.49 | 0.63 | 0.65 |
| 240 | GSE10289 | Cells silenced for SDHB expression and tumor phenotype | 0.60 | 0.62 | -0.87 | 0.55 | 0.59 | 0.62 | 0.59 | 0.30 | 0.45 |
| 241 | GSE15205 | TGF or TNF Time series in ARPE19 | 0.56 | 0.56 | 0.48 | 0.58 | 0.55 | -0.44 | 0.36 | 0.66 | 0.64 |
| 242 | GSE20948 | The Effect of Hepatitis C Virus Infection on Host Gene Expression | 0.57 | 0.58 | 0.46 | -0.13 | 0.56 | 0.44 | 0.15 | 0.61 | 0.57 |
| 243 | GSE11428 | Expression data from LNCaP and abl cells | 0.52 | 0.33 | 0.27 | 0.37 | 0.48 | 0.44 | 0.22 | 0.61 | 0.48 |
| 244 | GSE35659 | A transcriptional map of the impact of endurance exercise training on skeletal muscle phenotype (resting muscle after endurance training) | 0.39 | 0.50 | 0.50 | 0.12 | 0.34 | 0.20 | 0.55 | 0.48 | 0.58 |
| 245 | GSE31912 | Gene expression profile in MCF7 breast cancer cells after 78 functionallly important molecules were knocked down using siRNA. | 0.51 | 0.54 | 0.45 | 0.49 | 0.37 | 0.31 | -0.05 | 0.54 | 0.33 |
| 246 | GSE11919 | Vitamin C-induced gene expression profiling in GM5659 human skin fibroblasts | 0.59 | 0.49 | 0.66 | 0.69 | 0.48 | -0.01 | 0.64 | 0.70 | 0.61 |
| 247 | GSE34628 | Gene expression timecourse from Dengue virus infected human endothelial cells | 0.67 | 0.77 | 0.67 | 0.63 | 0.68 | 0.55 | 0.43 | 0.57 | 0.73 |
| 248 | GSE11510 | Taxonomy of placenta cells | 0.55 | 0.58 | 0.54 | 0.48 | 0.35 | 0.46 | -0.41 | 0.64 | 0.32 |
| 249 | GSE17251 | human | 0.57 | 0.53 | 0.57 | 0.07 | 0.35 | 0.48 | -0.03 | 0.59 | 0.58 |
| 250 | GSE21545 | Biobank of Karolinska Endarterectomy (BiKE) | 0.59 | 0.57 | 0.59 | 0.52 | 0.53 | -0.73 | 0.52 | 0.62 | 0.64 |
| 251 | GSE9055 | Time course gene expression of HUVEC after TNF-alpha treatment | 0.50 | 0.53 | 0.48 | 0.15 | 0.50 | 0.04 | 0.31 | 0.46 | 0.49 |
| 252 | GSE35716 | 4HC] | 0.56 | 0.58 | 0.56 | 0.52 | 0.54 | -0.64 | 0.38 | 0.61 | 0.63 |
| 253 | GSE17385 | Gene expression profiling from MM1.S cells with control or beta-catenin knockdown. | 0.61 | -0.62 | 0.09 | 0.59 | 0.60 | 0.60 | 0.61 | 0.66 | 0.66 |
| 254 | GSE30531 | Expression data of A375 melanoma cells after DMSO or MLN4924 treatment from 1 hour to 24 hour | 0.56 | 0.50 | 0.45 | 0.19 | 0.56 | 0.34 | -0.14 | 0.64 | 0.30 |
| 255 | GSE2964 | Motexafin Gadolinium and Zinc Induce Oxidative Stress Responses and Apoptosis in B-Cell Lymphoma Lines | 0.49 | -0.33 | 0.51 | 0.54 | 0.39 | 0.54 | 0.31 | 0.61 | -0.05 |
| 256 | GSE8192 | The DExH-box RNA helicase RHAU is a Nuclear Protein Involved in Transcription and mRNA Decay | 0.38 | 0.52 | 0.52 | 0.37 | 0.44 | 0.57 | -0.39 | 0.61 | 0.48 |
| 257 | GSE4316 | Genome-wide expression profile of human trabecular meshwork cultured cells, non-glaucomatous and POAG tissue | 0.64 | 0.58 | 0.72 | 0.74 | 0.65 | 0.11 | 0.69 | 0.79 | 0.79 |
| 258 | GSE6281 | Gene expression time-course in the human skin during elicitation of allergic contact dermatitis | 0.54 | 0.53 | 0.54 | 0.47 | 0.37 | 0.36 | -0.25 | 0.60 | 0.57 |
| 259 | GSE18995 | Expression data from donor lungs of cardiac death and brain death donors | 0.37 | 0.38 | 0.44 | 0.20 | 0.25 | 0.28 | 0.47 | 0.41 | 0.25 |
| 260 | GSE11430 | AffymetrixDataset | 0.50 | 0.59 | 0.53 | 0.58 | 0.56 | -0.59 | 0.18 | 0.58 | 0.61 |
| 261 | GSE9927 | Chronic CD4+ T cell Activation & Depletion in HIV-1 Infection: Type I Interferon-Mediated Disruption of T Cell Dynamics | 0.45 | 0.37 | -0.11 | 0.43 | 0.52 | 0.43 | 0.25 | 0.54 | 0.47 |
| 262 | GSE10315 | Multipotent mesenchymal stromal cells: identification of pathways common to TGFŒ≤3/BMP2-induced chondrogenesis | 0.46 | 0.51 | 0.45 | 0.34 | 0.45 | -0.29 | 0.41 | 0.54 | 0.61 |
| 263 | GSE14491 | TGFŒ≤/mutant-p53 jointly controlled genes | 0.55 | 0.39 | 0.55 | 0.53 | 0.45 | -0.03 | -0.16 | 0.59 | 0.61 |
| 264 | GSE6519 | Microarray Analysis of Baboon neonates consuming long-chain polyunsaturated fatty acid formula | 0.59 | -0.09 | 0.05 | 0.55 | 0.46 | 0.61 | 0.50 | 0.66 | 0.21 |
| 265 | GSE4107 | Expression profiling in early onset colorectal cancer | 0.19 | 0.48 | 0.42 | 0.39 | 0.41 | -0.13 | 0.48 | 0.06 | 0.44 |
| 266 | GSE11292 | High-time-resolution dynamic analysis of human regulatory T cell (Treg) / CD4+ T-effector cell (Teff) activation | 0.48 | -0.01 | 0.13 | 0.49 | 0.44 | 0.37 | 0.30 | 0.52 | 0.47 |
| 267 | GSE11959 | Anti-IGF-IR antibody h10H5 induces a unique transcriptional profile in SK-N-AS human neuroblastoma xenograft tumor | 0.65 | 0.41 | 0.60 | 0.47 | 0.58 | -0.01 | 0.38 | 0.71 | 0.63 |
| 268 | GSE27838 | Gene expression of expanded and non-expanded natural killer cells from healthy donor and myeloma patients | 0.51 | 0.44 | -0.55 | 0.50 | 0.52 | 0.48 | 0.16 | 0.60 | 0.59 |
| 269 | GSE41828 | TWEAK-treated time course in U2OS cells. | 0.49 | 0.48 | 0.42 | 0.46 | 0.34 | 0.45 | -0.64 | 0.53 | 0.43 |
| 270 | GSE41663 | Re-analysis by microarray using cDNA target of samples from psoriasis patients enrolled in an etanercept trial | 0.54 | 0.30 | 0.50 | 0.45 | -0.08 | 0.51 | -0.23 | 0.57 | 0.55 |
| 271 | GSE44029 | Expression data from SW480 cells with Gankyrin knockdown | 0.52 | 0.04 | 0.53 | 0.49 | -0.66 | 0.52 | 0.54 | 0.54 | 0.55 |
| 272 | GSE23103 | HeLa SCY1-like 1 esiRNA knockdown | 0.60 | 0.60 | 0.59 | 0.59 | 0.57 | 0.55 | -0.90 | 0.67 | 0.64 |
| 273 | GSE39059 | Changes in microRNA and mRNA expression with differentiation of human bronchial epithelial cells [mRNA] | 0.14 | 0.50 | 0.55 | 0.50 | 0.52 | 0.53 | -0.77 | -0.26 | 0.59 |
| 274 | GSE28005 | Charaterization of the initial molecular events of adipose tissue development and growth during overfeeding in humans | 0.54 | 0.53 | 0.53 | 0.42 | 0.23 | 0.10 | 0.25 | 0.60 | 0.55 |
| 275 | GSE48350 | Alzheimer's Disease Dataset | 0.47 | -0.18 | 0.45 | 0.35 | 0.34 | 0.41 | 0.07 | 0.51 | 0.41 |
| 276 | GSE30188 | Rho transcription inhibitor CCG-1423 effect on PC-3 cells | 0.51 | 0.49 | -0.66 | 0.51 | 0.48 | 0.51 | 0.06 | 0.59 | 0.55 |
| 277 | GSE37364 | Expression data from human colonic biopsy samples (adenoma-carcinoma) | 0.23 | 0.24 | 0.10 | 0.46 | 0.25 | 0.43 | 0.24 | 0.37 | 0.19 |
| 278 | GSE18271 | Analysis of TALE homeobox genes in neuroblastic tumors: ganglioneuroblastoma and ganglioneuroma | 0.48 | -0.53 | 0.50 | 0.41 | 0.39 | 0.41 | 0.19 | 0.56 | 0.38 |
| 279 | GSE47855 | Gene expression analysis for CD56- T, NK, CD56+ T cells, and iNKT cells | 0.35 | 0.35 | -0.01 | 0.32 | 0.34 | 0.39 | 0.10 | 0.54 | 0.56 |
| 280 | GSE10718 | Time course of NHBE cells exposed to whole cigarette smoke (full flavor) | 0.43 | 0.48 | -0.15 | 0.21 | 0.39 | 0.42 | 0.01 | 0.48 | 0.27 |
| 281 | GSE7637 | Expression data from human mesenchymal stem cells (#4F1560) | 0.43 | 0.46 | 0.49 | 0.28 | 0.47 | -0.50 | 0.18 | 0.57 | 0.47 |
| 282 | GSE20297 | The effects of terbutaline or GW9508 on TNF-alpha and IFN gamma (TNF-alpha + IFN gamma) stimulation by HaCaT | 0.49 | 0.47 | 0.45 | 0.47 | 0.11 | 0.48 | -0.71 | 0.57 | 0.56 |
| 283 | GSE10879 | Expression data of hormone-responsive MCF-7 cells versus estrogen-deprived MCF-7:5C and MCF-7:2A breast cancer cells | 0.34 | 0.52 | -0.04 | 0.49 | 0.40 | -0.45 | 0.50 | 0.40 | 0.50 |
| 284 | GSE47685 | Gene silencing of BSK65-MONO1 (RNF185) and of its natural antisense RNA (RNF185-AS) using siRNAs | 0.66 | 0.57 | 0.67 | 0.67 | -0.21 | 0.58 | 0.46 | 0.65 | 0.43 |
| 285 | GSE8671 | Transcriptome profile of human colorectal adenomas. | 0.44 | 0.12 | 0.22 | 0.44 | 0.37 | 0.47 | 0.04 | 0.46 | 0.33 |
| 286 | GSE13070 | Human Insulin Resistance and Thiazolidinedione-Mediated Insulin Sensitization | 0.46 | 0.44 | 0.45 | 0.00 | 0.44 | -0.46 | 0.41 | 0.54 | 0.53 |
| 287 | GSE33950 | SHARP1 suppresses breast cancer metastasis by promoting degradation of hypoxia-inducible factors | 0.40 | 0.50 | 0.49 | 0.37 | 0.25 | -0.80 | 0.49 | 0.34 | 0.22 |
| 288 | GSE17612 | Comparison of post-mortem tissue from brain BA10 region between schizophrenic and control patients. | 0.37 | -0.30 | 0.41 | 0.31 | 0.20 | 0.36 | 0.28 | 0.44 | 0.36 |
| 289 | GSE49910 | An Expression Atlas of Human Primary Cells: Inference of Gene Function from Coexpression Networks | 0.50 | 0.44 | 0.43 | 0.46 | 0.44 | -0.16 | -0.44 | 0.57 | 0.53 |
| 290 | GSE41296 | Characterization of Formaldehyde's Genotoxic Mode of Action by Gene Expression Analysis in TK6 Cells | 0.45 | -0.19 | 0.19 | 0.45 | 0.43 | 0.38 | -0.11 | 0.53 | 0.51 |
| 291 | GSE42046 | TWEAK-treated time course in ACHN cells | 0.49 | 0.45 | 0.32 | 0.40 | 0.42 | 0.27 | -0.78 | 0.57 | 0.45 |
| 292 | GSE10311 | Systematic Assessment of Human Osteoblast Transcriptome in Resting and Induced Primary Cells | 0.62 | 0.61 | 0.60 | 0.08 | 0.64 | 0.08 | 0.61 | 0.69 | 0.61 |
| 293 | GSE15918 | Torcetrapib induces aldosterone and cortisol production in an intracellular calcium-dependent mechanism | 0.45 | 0.42 | 0.47 | -0.11 | -0.47 | 0.46 | 0.32 | 0.53 | 0.14 |
| 294 | GSE11238 | Vaccinia E3L mutant virus infected HeLa cell lines (langl-affy-human-215499) | 0.42 | 0.35 | 0.06 | 0.46 | 0.45 | -0.46 | 0.23 | 0.53 | 0.15 |
| 295 | GSE12548 | EMT Time series in ARPE19 | 0.38 | 0.47 | 0.39 | 0.39 | 0.45 | -0.60 | 0.15 | 0.56 | 0.43 |
| 296 | GSE20318 | YWHAZ is an Invasion and Metastasis promoting genes of Lung cancer | 0.76 | 0.72 | 0.51 | 0.72 | 0.68 | -0.09 | 0.74 | 0.78 | 0.45 |
| 297 | GSE15013 | Expression of HOXB genes is significantly different in acute myeloid leukemia with a partial tandem duplication of MLL vs. a MLL translocation: a cross-laboratory study | 0.39 | -0.11 | 0.39 | 0.40 | 0.31 | 0.19 | -0.18 | 0.47 | 0.51 |
| 298 | GSE3202 | MK886 treatment of H720 non-small cell lung cancer cell line | 0.42 | -0.04 | 0.24 | 0.42 | 0.46 | -0.21 | 0.29 | 0.49 | 0.23 |
| 299 | GSE32473 | Gene expression is differently affected by pimecrolimus and betamethasone in lesional skin of atopic dermatitis. | 0.36 | 0.37 | 0.40 | 0.35 | -0.15 | 0.37 | -0.34 | 0.50 | 0.36 |
| 300 | GSE47751 | Early tissue responses to etanercept in psoriasis lesions | 0.43 | 0.22 | 0.38 | 0.36 | -0.04 | 0.38 | -0.38 | 0.47 | 0.39 |
| 301 | GSE33050 | GlcNAcylation of histone H2B facilitates its monoubiquitination [Affymetrix data] | 0.43 | 0.39 | 0.39 | 0.49 | 0.24 | 0.45 | -0.80 | 0.51 | -0.05 |
| 302 | GSE13564 | Gene expression in the human prefrontal cortex during postnatal development | 0.43 | -0.25 | 0.42 | 0.31 | 0.36 | -0.34 | 0.37 | 0.51 | 0.45 |
| 303 | GSE5563 | Gene expression profile of VIN lesions in comparison to controls | 0.46 | 0.22 | 0.36 | 0.33 | 0.36 | 0.39 | -0.54 | 0.50 | 0.49 |
| 304 | GSE32876 | Inferring transcriptional and microRNA-mediated regulatory programs in glioblastoma | 0.35 | 0.39 | -0.16 | 0.09 | 0.41 | 0.33 | -0.25 | 0.42 | 0.40 |
| 305 | GSE8056 | Gene Expression Profiles in Thermally Injured Human Skin: A Temporal Microarray Analysis | 0.45 | 0.41 | 0.49 | 0.47 | 0.52 | 0.37 | -0.52 | 0.59 | 0.41 |
| 306 | GSE27390 | Human bone marrow-derived mononuclear cells (BMMC): rheumatoid arthritis vs. osteoarthritis | 0.37 | 0.38 | 0.39 | 0.35 | 0.30 | -0.58 | -0.14 | 0.46 | 0.33 |
| 307 | GSE15132 | Riboflavin depletion impairs cell proliferation in intestinal cells: Identification of mechanisms and consequences | 0.38 | 0.45 | 0.22 | 0.37 | 0.35 | -0.20 | -0.27 | 0.50 | -0.02 |
| 308 | GSE44765 | Global profiling of human hair follicle scalp dermal papilla cells using Affymetrix microarrays | 0.75 | 0.69 | 0.69 | 0.42 | 0.76 | 0.70 | 0.41 | 0.80 | 0.82 |
| 309 | GSE51524 | LNCaP prostate cancer cell lines overexpressing wild-type or GARRPR-mutant Bag-1L | 0.38 | -0.56 | 0.37 | 0.40 | 0.39 | -0.36 | 0.35 | 0.47 | 0.47 |
| 310 | GSE32526 | Expression data from breast cancer tumor-initiating cells | 0.40 | 0.40 | 0.28 | 0.40 | 0.40 | -0.28 | -0.66 | 0.43 | 0.17 |
| 311 | GSE8687 | Inhibition of activation of Sez-4 cell line with IL-2 by Jak kinase inhibitors. | 0.32 | 0.29 | 0.28 | 0.36 | -0.17 | 0.30 | -0.47 | 0.33 | 0.13 |
| 312 | GSE7216 | Cytokine treated normal human epidermal keratinocytes | 0.28 | 0.07 | 0.28 | 0.18 | 0.01 | 0.37 | -0.32 | 0.43 | 0.31 |
| 313 | GSE3151 | Oncogene Signature Dataset | 0.16 | 0.22 | 0.25 | 0.06 | -0.02 | 0.07 | 0.12 | 0.29 | 0.28 |
| 314 | GSE12293 | Evolution of neuronal and endothelial transcriptomes in primates | 0.37 | -0.42 | 0.36 | 0.30 | -0.38 | 0.33 | 0.29 | 0.44 | 0.41 |
| 315 | GSE49353 | Evaluating cross-hybridization of murine cDNA to the Affymetrix Human Genome U133 Plus 2.0 chipset | -0.33 | 0.25 | 0.25 | 0.34 | 0.32 | -0.45 | 0.33 | -0.09 | -0.19 |
| 316 | GSE15520 | The Role of Cholesterol Pathways in Norovirus Replication | 0.28 | 0.53 | 0.42 | 0.50 | 0.54 | 0.44 | -0.77 | 0.28 | -0.13 |
| 317 | GSE22779 | Gene expression data of non-leukemic individuals before and during in-vivo glucocorticoid treatment | 0.25 | -0.02 | 0.01 | 0.23 | 0.24 | -0.16 | 0.02 | 0.36 | 0.38 |
| 318 | GSE31681 | Human cumulus cells | 0.30 | 0.28 | 0.23 | 0.06 | 0.00 | -0.38 | 0.23 | 0.40 | 0.27 |
| 319 | GSE34599 | In-transit extremity melanoma III | 0.54 | 0.50 | 0.52 | 0.26 | 0.18 | 0.21 | 0.35 | 0.60 | 0.53 |
| 320 | GSE13987 | Profile of rolipram treated B-CLL, normal B, and normal T cells | 0.28 | 0.25 | 0.30 | -0.37 | -0.54 | 0.28 | 0.26 | 0.39 | 0.37 |
| 321 | GSE24337 | The Human Airway Epithelial Basal Cell Transcriptome | -0.14 | 0.19 | -0.07 | 0.21 | 0.26 | 0.22 | -0.24 | 0.03 | 0.25 |
| 322 | GSE10739 | LPS and PMA response in parental MM6 cells | 0.34 | 0.33 | -0.64 | 0.33 | 0.33 | 0.33 | -0.55 | 0.44 | -0.25 |
| 323 | GSE11864 | Effect of interferon-gamma on macrophage differentiation and response to Toll-like receptor ligands | 0.32 | 0.31 | 0.20 | 0.20 | 0.28 | -0.47 | -0.53 | 0.42 | 0.37 |
| 324 | GSE29368 | CD140a+ human oligodendrocyte progenitor cells | 0.29 | -0.40 | 0.23 | -0.48 | 0.25 | 0.08 | 0.25 | 0.33 | 0.36 |
| 325 | GSE46873 | Dual targeting of MYC and CYCLON by BET bromodomain inhibition optimizes Rituximab response in lymphoma. | 0.45 | 0.38 | -0.58 | 0.26 | 0.41 | 0.43 | 0.31 | 0.37 | 0.21 |
| 326 | GSE15824 | Gene expression profiling of human gliomas and human glioblastoma cell lines | 0.26 | 0.24 | -0.03 | 0.28 | 0.30 | 0.19 | -0.44 | 0.39 | 0.23 |
| 327 | GSE8139 | Expression data from MCF7/HER2-18 xenografts | 0.03 | 0.10 | -0.03 | -0.04 | 0.06 | -0.04 | 0.13 | 0.13 | 0.21 |
| 328 | GSE33585 | Expression data from monocytic cell lines (THP) | 0.25 | 0.27 | -0.39 | 0.25 | 0.05 | 0.26 | -0.58 | 0.38 | 0.35 |
| 329 | GSE19278 | 2] | 0.41 | 0.29 | -0.06 | 0.36 | 0.34 | 0.31 | -0.51 | 0.48 | 0.48 |
| 330 | GSE9517 | Cysteine deprivation in liver cell line | 0.33 | 0.08 | 0.32 | 0.03 | 0.02 | 0.37 | -0.10 | 0.31 | 0.33 |
| 331 | GSE52158 | Dynamic developmental signaling logic underlying lineage bifurcations during human endoderm induction and patterning from pluripotent stem cells [Expression data set] | 0.21 | 0.14 | 0.22 | 0.08 | -0.38 | 0.15 | -0.33 | 0.31 | 0.15 |
| 332 | GSE24468 | Elucidation of the Mechanisms by which the Progesterone Receptor Inhibits Inflammatory Responses in Cellular Models of Breast Cancer | 0.12 | -0.20 | -0.09 | -0.03 | -0.02 | -0.04 | 0.01 | 0.27 | 0.23 |
| 333 | GSE10070 | Gene Expression in MCF10A cells through Differentiation on Transwells | 0.32 | 0.29 | -0.41 | 0.23 | 0.26 | 0.31 | -0.36 | 0.36 | 0.36 |
| 334 | GSE18235 | Effect of 10 Cigarette Smoke Condensates on Primary Human Airway Epithelial Cells | 0.44 | 0.40 | -0.32 | 0.35 | 0.38 | 0.45 | 0.02 | 0.47 | 0.30 |
| 335 | GSE23610 | Gene expression profiles of MCF-7 cells treated with Si-Wu-Tang, estradiol and ferulic acid | 0.08 | -0.32 | -0.32 | -0.02 | 0.04 | -0.18 | 0.06 | 0.17 | 0.17 |
| 336 | GSE36701 | Gene expression analysis of rectal mucosa in chronic irritable bowel syndrome (IBS) compared to healthy volunteers (HV) | 0.39 | 0.52 | 0.54 | 0.57 | 0.18 | 0.60 | 0.53 | 0.65 | 0.62 |
| 337 | GSE17400 | Dynamic Innate Immune Responses of Human Bronchial Epithelial Cells against SARS-CoV and DOHV infection | 0.35 | 0.64 | 0.29 | 0.50 | 0.59 | 0.66 | 0.59 | 0.15 | 0.39 |
| 338 | GSE40266 | Expression data from TGF-beta-treated human ovarian fibroblasts | 0.04 | -0.33 | -0.19 | 0.03 | 0.08 | -0.37 | 0.09 | 0.15 | 0.02 |
| 339 | GSE11941 | Topoisomerase II inhibition involves characteristic chromosomal expression patterns: Trovafloxacin study | 0.65 | 0.73 | 0.57 | 0.52 | 0.74 | 0.70 | 0.47 | 0.79 | 0.81 |
| 340 | GSE53603 | Expression data from SKOV3 cells treated with SAHA or vehicle control | 0.61 | 0.51 | 0.63 | 0.58 | 0.43 | 0.29 | -0.09 | 0.59 | 0.49 |
| 341 | GSE10281 | Letrozole (Femara) early response to treatment | 0.50 | 0.48 | 0.44 | 0.07 | 0.37 | 0.50 | 0.42 | 0.42 | 0.52 |
| 342 | GSE8784 | Plasmodium Circumsporozoite Protein Promotes the Development of the Liver Stages of the Parasite | 0.28 | -0.29 | 0.19 | 0.30 | 0.18 | 0.29 | -0.43 | 0.42 | 0.36 |
| 343 | GSE32967 | Modeling lethal prostate cancer variant with small cell carcinoma features [expression profile] | 0.65 | 0.32 | 0.28 | 0.61 | 0.47 | 0.54 | 0.63 | 0.71 | 0.72 |
| 344 | GSE25619 | Gene expression profiles of granulin- and control-treated normal human mammary fibroblasts | 0.72 | 0.66 | 0.66 | 0.26 | 0.59 | 0.53 | 0.41 | 0.72 | 0.74 |
| 345 | GSE16480 | Inactivation of CDK2 is synthetic lethal to MYCN-overexpressing cancer cells | 0.46 | 0.56 | 0.44 | 0.55 | 0.53 | 0.54 | -0.05 | 0.63 | 0.41 |
| 346 | GSE9984 | Profiling Gene Expression in Human Placentae of Different Gestational Ages: an OPRU Network and UW SCOR Study | 0.64 | 0.64 | 0.36 | 0.64 | 0.65 | 0.16 | 0.44 | 0.63 | 0.69 |
| 347 | GSE33495 | Disrupted transcripitonal network in ŒîNp63 AEC tissue model [gene expression] | 0.67 | 0.60 | 0.28 | 0.61 | 0.07 | 0.64 | 0.24 | 0.67 | 0.66 |
| 348 | GSE12963 | Gene expression in human CD4+ T-lymphocytes infected with VSVG-pseudotyped HIV-1 viruses lacking Env, Vpr, and Nef | 0.62 | 0.56 | -0.14 | 0.53 | 0.45 | 0.55 | 0.62 | 0.63 | 0.49 |
| 349 | GSE9250 | Genomic profiling in CLL and subtypes of del13q14 | 0.63 | 0.21 | 0.46 | 0.50 | 0.51 | 0.35 | 0.56 | 0.63 | 0.59 |
| 350 | GSE31215 | Gene expression analysis of human pediatric mesenchymal stem cells (hpMSCs) upon expression of EWS-FLI-1 | 0.45 | 0.52 | 0.56 | 0.49 | 0.56 | 0.26 | 0.44 | 0.51 | 0.43 |
| 351 | GSE4737 | HCaRG vs NEO | 0.69 | 0.70 | 0.66 | 0.19 | 0.54 | 0.51 | 0.63 | 0.71 | 0.63 |
| 352 | GSE30439 | Exposure of cystic fibrosis bronchial epithelial cells (CFBE 41 o-) to Pseudomonas aeruginosa (PA01) biofilms | 0.63 | 0.54 | 0.27 | 0.60 | 0.55 | 0.57 | 0.09 | 0.64 | 0.60 |
| 353 | GSE40986 | Gene expression profiles induced by overexpression of PDEF in MCF10A mammary epithelial cell line | 0.56 | -0.39 | 0.15 | 0.62 | 0.58 | 0.62 | 0.59 | 0.52 | 0.48 |
| 354 | GSE38718 | Sex and aging effect on skeletal muscle transcriptome in humans | 0.38 | 0.39 | 0.37 | -0.08 | 0.40 | -0.16 | 0.30 | 0.40 | 0.40 |
| 355 | GSE33112 | Gene expression in colon cancer stem cells (CSC) cultures identified by Wnt signaling levels | 0.62 | 0.59 | 0.67 | 0.62 | 0.65 | 0.27 | 0.36 | 0.71 | 0.48 |
| 356 | GSE8640 | TFAP2C regulates multiple pathways of estrogen signaling | 0.45 | 0.56 | 0.37 | 0.35 | 0.35 | 0.41 | -0.17 | 0.60 | 0.57 |
| 357 | GSE22148 | Induced Sputum Genes Associated With Spirometroc and Radiological Disease Severity in COPD Ex-smokers | 0.43 | 0.38 | 0.40 | 0.43 | 0.36 | -0.22 | 0.06 | 0.50 | 0.50 |
| 358 | GSE57552 | ZFX silencing introduced differential gene expression in leukemia cells | 0.47 | 0.48 | 0.45 | 0.30 | -0.01 | 0.48 | -0.80 | 0.53 | 0.54 |
| 359 | GSE24530 | Identification and Characterization of Subpopulations within Human Embryonic Stem Cell Lines | 0.65 | 0.68 | 0.70 | 0.52 | 0.37 | 0.30 | 0.59 | 0.75 | 0.61 |
| 360 | GSE2817 | Wavelet modelling of microarray data provides chromosomal pattern of expression which predicts survival in gliomas | 0.43 | 0.27 | 0.27 | 0.38 | 0.16 | 0.10 | 0.43 | 0.57 | 0.45 |
| 361 | GSE29330 | Identification of GNG7 as An Epigenetically Silenced Gene in Head and Neck Cancer by Gene Expression Profiling | 0.59 | 0.57 | 0.60 | 0.26 | 0.25 | 0.53 | 0.23 | 0.54 | 0.56 |
| 362 | GSE33424 | Expression data from human cord blood CD161++/CD161+/CD161- CD8+ T cell subsets | 0.45 | 0.13 | 0.45 | 0.09 | -0.51 | 0.42 | 0.40 | 0.54 | 0.47 |
| 363 | GSE41035 | FGFR3-shRNA induced transcriptional changes in RT112 bladder cancer cells | 0.51 | -0.11 | 0.25 | 0.49 | 0.48 | 0.54 | 0.15 | 0.60 | 0.59 |
| 364 | GSE31782 | Knock-down and Over-expression of JMJD6 in MCF-7 and/or MDA-MB231 | 0.22 | 0.52 | 0.52 | 0.47 | 0.47 | 0.44 | -0.38 | 0.29 | -0.08 |
| 365 | GSE13274 | Ad-HER-wt and Ad-HER2-ki infected HMECs | 0.51 | 0.41 | 0.50 | 0.12 | 0.45 | 0.40 | -0.07 | 0.54 | 0.57 |
| 366 | GSE36287 | Expression data from primary human keratinocytes exposed to cytokines in vitro (IL-4, IL-13, IL-17A, IFN-alpha, IFN-gamma, TNF) | 0.47 | 0.60 | 0.52 | 0.53 | 0.22 | 0.60 | 0.32 | 0.64 | 0.21 |
| 367 | GSE16237 | Expression data of human neuroblastoma tissue samples | 0.51 | 0.11 | 0.47 | 0.48 | 0.52 | 0.19 | 0.44 | 0.63 | 0.47 |
| 368 | GSE21979 | Transcriptional and post-transcriptional regulation of VEGF by the unfolded protein response | 0.63 | 0.60 | 0.43 | 0.56 | 0.56 | 0.60 | -0.23 | 0.61 | 0.53 |
| 369 | GSE39902 | Role of TAZ as mediator of Wnt signaling (MII) | 0.55 | 0.52 | 0.59 | 0.24 | 0.54 | 0.49 | -0.13 | 0.48 | 0.55 |
| 370 | GSE8685 | Activation of Sez-4 cell line with IL-2, IL-15 or IL-21. | 0.20 | 0.31 | 0.26 | 0.28 | -0.21 | 0.34 | -0.45 | 0.43 | 0.40 |
| 371 | GSE6140 | Cross platform microarray analysis for robust identification of differentially expressed genes | 0.01 | 0.73 | 0.74 | 0.73 | 0.70 | 0.66 | 0.70 | -0.41 | -0.34 |
| 372 | GSE14668 | B-Cell Gene Signature with Massive Intrahepatic Production of Antibodies to Hepatitis B Core Antigen in HBV-Associated Acute Liver Failure | 0.55 | 0.41 | 0.43 | 0.60 | 0.43 | 0.57 | 0.39 | 0.63 | 0.65 |
| 373 | GSE11367 | Effect of IL-17 on human vascular smooth muscle cells | 0.62 | 0.56 | 0.54 | 0.12 | 0.42 | 0.58 | 0.32 | 0.66 | 0.72 |
| 374 | GSE21668 | Expression data from undifferentiated human embryonic stem cells (hESC) and Day 3.5 mesodermal progenitor (CD326neg CD56+) population | 0.31 | 0.72 | 0.61 | 0.76 | 0.70 | 0.55 | 0.68 | 0.79 | 0.75 |
| 375 | GSE24422 | Effect of insulin on the stromal and adipocyte cells within hMSC derived adipocytes | 0.60 | 0.43 | 0.42 | 0.18 | 0.31 | 0.56 | 0.04 | 0.63 | 0.56 |
| 376 | GSE41802 | Isocitrata Dehydrogenase (IDH) Mutations Promote a Reversible ZEB1/mir-200-Dependent Epithelial Mesenchymal Transition (EMT) | 0.42 | 0.60 | 0.47 | 0.50 | 0.35 | 0.44 | 0.45 | 0.63 | 0.42 |
| 377 | GSE5486 | Using GINI2 to identify novel mutations in candidate tumor suppressor genes in colon cancer cells | 0.60 | 0.50 | 0.38 | 0.07 | 0.44 | 0.41 | 0.49 | 0.42 | 0.23 |
| 378 | GSE19735 | Comparison of human embroynic stem cell derived vascular cells to mature human vascular and hematopoietic cells | 0.55 | 0.48 | 0.37 | 0.54 | -0.05 | -0.25 | 0.55 | 0.59 | 0.53 |
| 379 | GSE7874 | Effects of EPO and EST on erythroid maturation | -0.26 | 0.16 | 0.11 | 0.17 | 0.20 | -0.49 | 0.17 | 0.15 | 0.25 |
| 380 | GSE10046 | Breast cancer-associated fibroblasts confer AKT1-mediated epigenetic silencing of Cystatin M in epithelial cells. | 0.77 | 0.82 | 0.65 | 0.59 | 0.57 | 0.76 | 0.60 | 0.83 | 0.75 |
| 381 | GSE30127 | Establishment of human trophoblast progenitor cell lines from the chorion | 0.62 | 0.06 | 0.31 | 0.59 | 0.64 | 0.60 | 0.31 | 0.56 | 0.61 |
| 382 | GSE40873 | a prospective study | 0.54 | 0.08 | 0.55 | 0.32 | 0.48 | 0.48 | 0.15 | 0.59 | 0.60 |
| 383 | GSE11618 | Stable XIAP knockdown in HCT116 colon cancer cells | 0.57 | 0.46 | 0.27 | 0.58 | 0.59 | 0.50 | 0.11 | 0.65 | 0.66 |
| 384 | GSE30660 | The Effect of Repeated Whole Cigarette Smoke Challenge on Human Air-Liquid Interface Lung Epithelial Cultures | -0.40 | 0.26 | 0.22 | 0.21 | 0.32 | 0.33 | -0.53 | -0.24 | -0.04 |
| 385 | GSE9169 | Gene expression during neuronal differentiation in two subtypes of SH-SY5Y | 0.51 | 0.26 | 0.41 | 0.52 | 0.43 | 0.39 | 0.25 | 0.64 | 0.54 |
| 386 | GSE44807 | Gene expression data from primary human bronchial epithelial cells expressing EGFP or DN-GRHL2 | 0.61 | 0.50 | 0.60 | -0.30 | 0.58 | 0.04 | 0.61 | 0.18 | 0.65 |
| 387 | GSE7586 | Genome wide analysis of placental malaria | 0.38 | 0.52 | 0.47 | 0.06 | 0.45 | -0.20 | 0.53 | 0.46 | 0.56 |
| 388 | GSE53552 | Gene expression profiling in psoriatic lesional and non-lesional skin [brodalumab treatment] | 0.50 | 0.22 | 0.48 | 0.34 | 0.14 | 0.44 | -0.09 | 0.55 | 0.51 |
| 389 | GSE40281 | Signaling pathways of HPAIV | 0.55 | 0.61 | 0.61 | 0.55 | 0.31 | 0.61 | 0.39 | 0.64 | 0.21 |
| 390 | GSE15773 | Expression data from human adipose tissue | 0.52 | 0.42 | 0.46 | 0.44 | 0.11 | -0.24 | 0.39 | 0.58 | 0.44 |
| 391 | GSE39454 | Genomic signatures characterize leukocyte infiltration in myositis muscles | 0.48 | 0.42 | 0.47 | -0.01 | 0.27 | -0.28 | 0.37 | 0.44 | 0.55 |
| 392 | GSE41386 | Role of REST in the pathogenesis of uterine fibroids | 0.59 | 0.69 | 0.40 | 0.67 | 0.45 | 0.63 | 0.45 | 0.63 | 0.63 |
| 393 | GSE55529 | EcadEGFP expression in MDA-MB-134 and IPH-926 | 0.68 | 0.54 | 0.34 | 0.61 | 0.46 | 0.61 | 0.59 | 0.58 | 0.77 |
| 394 | GSE40730 | Genome-wide analysis of RNAs translationally regulated upon BRCA1 depletion in human mammary epithelial cells | 0.66 | 0.67 | 0.62 | 0.12 | 0.31 | 0.63 | 0.53 | 0.66 | -0.27 |
| 395 | GSE27128 | Expression levels in strained vs. non-strained Calu-3 lung epithelial cells | 0.57 | 0.61 | 0.60 | 0.49 | 0.53 | 0.54 | -0.14 | 0.52 | 0.13 |
| 396 | GSE32100 | Glioma cells oxygen response | 0.06 | -0.22 | -0.07 | -0.13 | 0.05 | -0.21 | -0.09 | 0.10 | 0.10 |
| 397 | GSE4600 | Identifying targets of MeCP2 during neuronal maturational differentiation | 0.48 | -0.38 | 0.22 | 0.41 | 0.49 | 0.47 | 0.53 | 0.54 | 0.49 |
| 398 | GSE10595 | Interaction of bone marrow stroma and monocytes: bone marrow stromal cell lines cultured with monocytes | 0.69 | 0.63 | 0.64 | 0.67 | 0.61 | -0.47 | 0.51 | 0.73 | 0.01 |
| 399 | GSE25087 | Human Fetal and Adult Peripheral Na√Øve CD4+ T cells and CD4+CD25+ Treg cells | 0.57 | 0.47 | 0.31 | 0.44 | 0.51 | -0.45 | 0.45 | 0.63 | 0.61 |
| 400 | GSE39843 | Expression data of cystic fibrosis and non-cystic fibrosis airway cell lines under oxidative stress | 0.64 | 0.58 | 0.45 | 0.29 | 0.46 | 0.20 | 0.45 | 0.66 | 0.68 |
| 401 | GSE10575 | Migratory chondrogenic progenitor cells from repair tissue during the later stages of human osteoarthritis | 0.54 | 0.52 | 0.37 | 0.24 | 0.50 | 0.20 | -0.19 | 0.62 | 0.46 |
| 402 | GSE5675 | Pilocytic astrocytoma | 0.42 | 0.35 | 0.45 | 0.38 | 0.40 | 0.20 | 0.45 | 0.61 | 0.56 |
| 403 | GSE23640 | Gene-expression profile of breast cancer cell lines and sorted breast cancer epithelial cells | 0.50 | 0.55 | 0.29 | 0.30 | 0.52 | 0.54 | 0.20 | 0.48 | 0.61 |
| 404 | GSE4975 | Expression data from p63 siRNA in squamous cell lines | 0.59 | 0.58 | 0.62 | 0.36 | -0.45 | 0.63 | 0.38 | 0.68 | 0.28 |
| 405 | GSE40968 | The effect of ACSL4 expression on overall gene expression in breast cancer cell lines | 0.68 | 0.61 | 0.62 | 0.55 | 0.31 | 0.48 | 0.50 | 0.70 | 0.66 |
| 406 | GSE10580 | Genes regulated by PRDM5 in U2OS cells. | 0.53 | 0.50 | -0.24 | 0.57 | 0.42 | 0.30 | 0.57 | 0.56 | 0.26 |
| 407 | GSE34828 | Expression data from fibroblast growth factor receptor 4 (FGFR4) knock down ovarian cancer cell lines | 0.66 | 0.70 | 0.67 | 0.24 | 0.35 | 0.34 | 0.65 | 0.61 | 0.32 |
| 408 | GSE20086 | Heterogeneity of gene expression in stromal fibroblasts of human breast carcinomas and normal breast | 0.63 | 0.30 | 0.52 | 0.36 | 0.39 | 0.51 | 0.53 | 0.67 | 0.64 |
| 409 | GSE11729 | H1299 EGF and Iressa stimulation | 0.49 | 0.55 | 0.47 | 0.53 | 0.39 | 0.19 | 0.05 | 0.49 | 0.11 |
| 410 | GSE50175 | Expression data from human Th1 and Th1Th17 cells | 0.63 | 0.06 | 0.40 | 0.64 | 0.55 | 0.61 | 0.33 | 0.66 | 0.69 |
| 411 | GSE43177 | MicroRNA regulate immunological pathways in T-cells in immune thrombocytopenia (ITP) [mRNA] | 0.68 | 0.16 | 0.47 | 0.66 | 0.32 | 0.67 | 0.66 | 0.71 | 0.71 |
| 412 | GSE41485 | Expression data of A939572 SCD1 inhibitor treated ccRCC cells | 0.54 | 0.48 | 0.33 | 0.42 | 0.69 | 0.52 | 0.54 | 0.74 | 0.37 |
| 413 | GSE35006 | Profiling of p53-responsive genes in human breast cancer cells harboring endogenous ts-p53 E285K | 0.73 | 0.53 | 0.20 | 0.65 | 0.66 | 0.58 | 0.63 | 0.70 | 0.70 |
| 414 | GSE14474 | The Effects of Static Magnetic Fields on Human Embryonic Cells | 0.33 | 0.54 | 0.60 | 0.19 | 0.62 | 0.28 | 0.51 | 0.62 | 0.54 |
| 415 | GSE8507 | Neutrophil and PBMC gene expression data from Job's Syndrome | 0.38 | 0.38 | 0.39 | 0.27 | 0.33 | 0.00 | -0.15 | 0.52 | 0.25 |
| 416 | GSE9101 | Expression data in native lipoprotein-stimulated human THP-1 macrophages | 0.29 | 0.36 | 0.28 | -0.31 | -0.43 | 0.36 | 0.32 | 0.44 | 0.38 |
| 417 | GSE40215 | shRNA knockdown of the transcription factor NF-YA (NFYA) | 0.52 | 0.51 | 0.54 | 0.51 | 0.35 | 0.54 | -0.82 | 0.59 | 0.56 |
